# Full ribosomal operon sequencing of anaerobic gut fungi (phylum *Neocallimastigomycota*): insights on its markers and phylogenetic resolution

**DOI:** 10.3897/imafungus.17.195921

**Published:** 2026-08-26

**Authors:** Diana Young, Katrin Stüer-Patowsky, Liren Huang, Mostafa S. Elshahed, Noha H. Youssef, Radwa Hanafy, Yanfen Cheng, Christina D. Moon, Priya Soni, Akshay Joshi, Marcus Stabel, Katrin Ochsenreither, Sumit Singh Dagar, Ethan Hillman, Kevin V. Solomon, Katerina O. Fliegerová, Gareth W. Griffith, Tony M. Callaghan, Sabine M. Podmirseg, Alexander Sczyrba, Veronika Flad, Michael Lebuhn, Christian Wurzbacher

**Affiliations:** 1 Department for Laboratory Analytics, Micro and Molecular Biology, Bavarian State Research Center for Agriculture, Freising, Germany Department of Internal Medicine, University of Michigan Ann Arbor United States of America; 2 Chair of Urban Water Systems Engineering, Technical University of Munich, Garching, Germany Department of Life Sciences, Aberystwyth University Aberystwyth United Kingdom; 3 Faculty of Technology and Center for Biotechnology (CeBiTec), Bielefeld University, Bielefeld, Germany Department of Microbiology and Molecular Genetics, Oklahoma State University Stillwater United States of America; 4 Department of Microbiology and Molecular Genetics, Oklahoma State University, Stillwater, Oklahoma, USA Department for Laboratory Analytics, Micro and Molecular Biology, Bavarian State Research Center for Agriculture Freising Germany; 5 Laboratory of Gastrointestinal Microbiology, College of Animal Science and Technology, Nanjing Agricultural University, Nanjing, Jiangsu, China Department of Chemical and Biomolecular Engineering, University of Delaware Newark United States of America; 6 AgResearch Group, Bioeconomy Science Institute, Palmerston North, New Zealand Faculty of Technology and Center for Biotechnology (CeBiTec), Bielefeld University Bielefeld Germany; 7 Center of Environmental Biotechnology, Institute for Chemistry and Biotechnology, Zürich University of Applied Sciences, Wädenswil, Switzerland IBG-5: Computational Metagenomics, Institute for Bio- and Geosciences, Forschungszentrum Jülich Jülich Germany; 8 Process Engineering in Life Sciences 2: Technical Biology, Karlsruhe Institute of Technology, Karlsruhe, Germany Process Engineering in Life Sciences 2: Technical Biology, Karlsruhe Institute of Technology Karlsruhe Germany; 9 Applied Logistics and Polymer Sciences: Pharmaceutical Biotechnology, University of Applied Sciences Kaiserslautern, Pirmasens, Germany Institute of Animal Physiology and Genetics, Czech Academy of Sciences Prague Czech Republic; 10 Bioenergy Group, Agharkar Research Institute, Gopal Ganesh Agarkar Road, Pune, India Department of Microbiology, University of Innsbruck Innsbruck Austria; 11 Department of Internal Medicine, University of Michigan, Ann Arbor, Michigan, USA Bioenergy Group, Agharkar Research Institute Pune India; 12 Department of Chemical and Biomolecular Engineering, University of Delaware, Newark, Delaware, USA Laboratory of Gastrointestinal Microbiology, College of Animal Science and Technology, Nanjing Agricultural University Nanjing China; 13 Institute of Animal Physiology and Genetics, Czech Academy of Sciences, Prague, Czech Republic Chair of Urban Water Systems Engineering, Technical University of Munich Garching Germany; 14 Department of Life Sciences, Aberystwyth University, Aberystwyth, Ceredigion SY23 3DD, Wales, UK AgResearch Group, Bioeconomy Science Institute Palmerston North New Zealand; 15 SomaTech Ltd. Ballybay Food Hub, Ballybay, County Monaghan, Ireland Center of Environmental Biotechnology, Institute for Chemistry and Biotechnology, Zürich University of Applied Sciences Wädenswil Switzerland; 16 Department of Microbiology, University of Innsbruck, Innsbruck, Austria Applied Logistics and Polymer Sciences: Pharmaceutical Biotechnology, University of Applied Sciences Kaiserslautern Pirmasens Germany; 17 IBG-5: Computational Metagenomics, Institute for Bio- and Geosciences, Forschungszentrum Jülich, Jülich, Germany SomaTech Ltd. Ballybay Food Hub Ballybay Ireland; 18 bioBit GmbH, Munich, Germany bioBit GmbH Munich Germany

**Keywords:** High-throughput sequencing, Nanopore and PacBio, phylogeny, *rrn* markers

## Abstract

The phylogenetic affiliations of anaerobic gut fungi (*Neocallimastigomycota*) are typically evaluated using single-gene markers. However, this approach often fails to resolve relationships between closely related lineages. To address this issue and identify alternative markers, we created a curated database comprising the complete ribosomal operon sequences of 156 isolates, representing 20 of the 22 recognized genera and two new genus-level clades. Using long-read sequencing, we obtained ~9 kbp operon sequences and developed a robust analysis pipeline. Incorporating both coding genes and non-coding regions (excluding IGS1) improved phylogenetic resolution. This phylogenetic approach successfully resolved the *Cyllamyces* and *Caecomyces* clades (hard-to-distinguish genetically), as well as seven analysed *Piromyces* species. We also scanned the operon for markers that are suitable for short-read sequencing platforms, with the aim of enhancing biodiversity and phylogenetic studies. Notably, the ETS1 genetic region also enabled the distinction between these lineages, indicating its phylogenetic value within the ribosomal operon. The resulting database is a valuable resource for expanding and strengthening phylogenetic frameworks.

## Introduction

Anaerobic gut fungi (AGF), or members of the phylum *Neocallimastigomycota*, are a distinctive group of fungi that inhabit the oxygen-deprived digestive tracts of larger herbivores. Many AGF genera appear to have evolved alongside grasses and the dietary transition of animals from insectivory to herbivory (Wang et al. 2019), although more recent evidence suggests that older lineages exist in cold-blooded reptiles (Pratt et al. 2023). AGF form syntrophic relationship with methanogenic archaea, influencing methane emissions from their animal hosts (Li et al. 2021), and play a crucial role in the degradation of fibrous plant materials in the gut (Xue et al. 2022; Liang et al. 2023). Although AGF share several morphological features with *Chytridiomycota*, including flagellated zoospores and zoosporangia (Wang et al. 2019), phylogenetic analyses support their classification as a distinct fungal phylum that diverged early from chytrid ancestors (James et al. 2006a; Hibbett et al. 2007; Powell and Letcher 2014).

To date, 22 genera have been formally described (Hanafy et al. 2022; Pratt et al. 2024) and recently three tortoise-affiliated new genera have been discovered (Morris et al. 2026). Several morphological features are considered taxonomically informative, including thallus morphology (filamentous or bulbous), the number of flagella per zoospore (mono- or polyflagellate) and the rhizoidal growth pattern (mono- or polycentric) (Hanafy et al. 2022). However, species identification using only morphology is often challenging and can be misleading due to the high degree of morphological similarity among species and even across genera. Furthermore, morphological characteristics such as cell and sporangium size may vary, and distinctive structures can be lost during repeated subculturing under laboratory conditions (Hanafy et al. 2023).

To overcome these limitations, recent studies have attempted to use phenotypic analysis to identify substrate utilization patterns that may aid in the description and differentiation of novel AGF species and genera (Joshi et al. 2018; Pratt et al. 2025). However, unlike bacteria, AGF lack standardized and comprehensive biochemical assays for characterizing metabolic diversity. Further research in this area could facilitate trait-based classification and enable the discrimination of closely related taxa. Nonetheless, our understanding of the metabolic behavior underlying phenotypic variation in AGF remains limited.

Current phylogenetic approaches also face limitations in resolving relationships among AGF taxa. A notable example is the branching pattern of the genera *Caecomyces* and *Cyllamyces*. These taxa are distinguished primarily by morphological features such as thallus development. Both genera are characterized by bulbous thalli with spherical holdfasts and can appear highly similar under microscopic examination. Ecologically, they also show substantial overlap, having been isolated from the same host species, including cattle, sheep, and water buffalo (Suppl. material 1: supplementary data 1). The primary morphological distinction is that *Cyllamyces* exhibits polycentric thalli (Ozkose et al. 2001), while *Caecomyces* presents monocentric thalli (Gold et al. 1988). Despite this clear morphological distinction, commonly used genetic regions have not consistently resolved the two genera as distinct lineages (Callaghan et al. 2015; Edwards et al. 2019; Hanafy et al. 2023).

DNA sequence data currently serves as the primary basis for species-level classification and the description of new species. Various regions within the ribosomal operon, which encode ribosomal RNA domains, are typically used for phylogenetic delimitation and identification. The ribosomal RNA operon (*rrn*) contains useful spliced and functional regions in its repeat units, which have evolved at different rates (Hillis and Dixon 1991). Moreover, the *rrn* has multiple tandem repeats (approximately 200 copies per AGF genome; Edwards et al. 2017), facilitating the detection of low-abundance species in a sample. This is one reason why the fungal *rrn* operon is the most prominent tool for taxonomy and phylogenetics, serving as a universal fungal genetic marker (Schoch et al. 2012).

**Figure 1.**
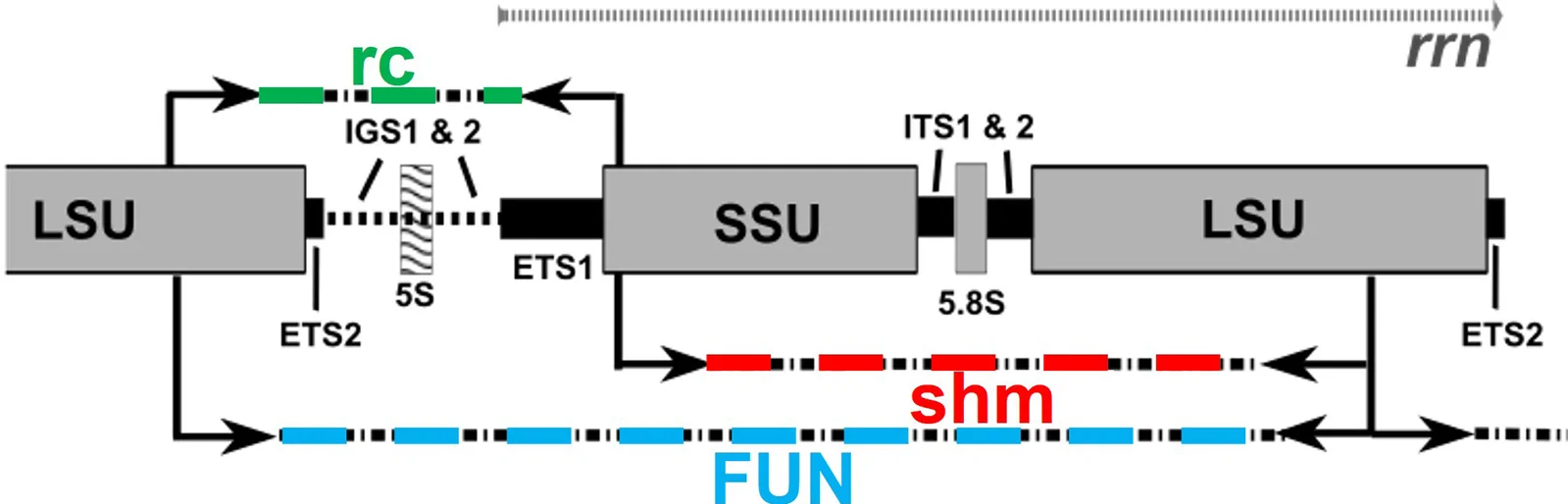
Schema of the whole nuclear ribosomal operon organized in tandem repeats. Comprises the small subunit (SSU), internal transcribed spacer 1 and 2 (ITS1, ITS2), 5.8S, large subunit (LSU), 5S rDNA unit, and intergenic spacers 1 and 2 (IGS1, IGS2), flanked by external transcribed spacers (ETS1, ETS2). The primer abbreviations (rc, shm, and FUN) are indicated for each amplicon. The grey blocks and the 5S are the coding regions. Black blocks and IGS are the non-coding regions. Figure modified from Wurzbacher et al. (2019).

The transcribed *rrn* operon comprises five regions: the gene for the small subunit (SSU); the internal transcribed spacer (ITS) regions; the 5.8S gene, which is located between the ITS1 and ITS2 regions; the large subunit (LSU) gene; and the external transcribed spacer (ETS) regions, located at both ends of the operon (ETS1 and ETS2 regions, also known as 5’ETS and 3’ETS, respectively). The ETS and ITS regions are spliced during the maturation of the rRNA (Henras et al. 2015). In fungi, the *rrn* copies are present as adjacent tandem repeats arranged in a single fungal chromosome. An intergenic (non-transcribed) spacer (IGS) is located between each of the *rrn* copies, separated into IGS1 and IGS2 by the ribosomal 5S subunit gene (Fig. 1; Wurzbacher et al. 2019).

The most commonly used genetic markers for AGF are: 1) the internal transcribed spacer regions (ITS1 and ITS2), considered the “universal” barcodes for *Fungi* (Schoch et al. 2012); and 2) domains of the 28S large subunit (LSU) gene, which is now preferred for AGF analysis (Hibbett et al. 2007; Dagar et al. 2011; Edwards et al. 2017, 2019). In particular, regions D1 and D2 have been useful for phylogenetic species separation (Dollhofer et al. 2016; Edwards et al. 2017; Hanafy et al. 2020a; Young et al. 2022).

In general, the SSU gene in *Fungi* is highly conserved, making it useful only for phylogenetic analyses aiming to resolve higher ranks (Boucher et al. 2004; Banos et al. 2018), except in a few groups (e.g. *Chytridiomycota*; Ishii et al. 2015). Earlier studies have also documented high levels of sequence similarity between SSU-AGF sequences, which limits the utility of SSU for accurate phylogenetic resolution (Dore and Stahl 1991). Similarly, the short 5.8S gene is highly conserved, with a limited number of phylogenetically informative sites. Nevertheless, it can be used to anchor for diverse ITS sequences and define affiliation at higher ranks (Heeger et al. 2019). Earlier studies on AGF phylogeny have used the ITS1 region for phylogenetic resolution. However, a key disadvantage of using the ITS region for the phylogenetic analysis of *Neocallimastigomycota* is that the multiple copies within the genome can vary by up to 13%, as observed in a Sanger-sequenced ITS1 clone library from a single strain (Callaghan et al. 2015). In addition, high length variability has been observed in the ITS sequence data across various AGF genera.

Recently, the ~700 bp fragment encompassing the D1 and D2 divergence regions of the 3.5 kbp large ribosomal subunit (D1/D2 LSU) was shown to provide better phylogenetic resolution than the ITS1 region. This is due to its consistent length across taxa and near-identical sequence similarity across copies in a single AGF strain (Dollhofer et al. 2016; Hanafy et al. 2020a; Young et al. 2022). However, the D1/D2 divergence region in the LSU gene often fails to distinguish between closely related species and provides uncertain resolution regarding the phylogenetic topology of some genera (e.g. *Joblinomyces*) (Hanafy et al. 2023).

The ITS and D1/D2 LSU are the most explored regions in AGF taxonomy and diversity studies. Sequences of both have been deposited and curated in public databases, such as UNITE for ITS and PR2/SILVA for D1/D2 LSU (Nilsson et al. 2019; Quast et al. 2013). However, other regions have received little attention thus far. The remaining regions, such as the ETS, the relatively short 5S (Hillis and Dixon 1991), and the IGS have not yet been thoroughly investigated as potential markers for resolving phylogenetic relationships within AGF or *Fungi* in general. Nevertheless, there are indications that these regions may be useful for investigating strain diversity. For example, Hausner et al. (2000) reported considerable IGS variability between *Piromyces* strains.

The suitability of using multiple genetic markers to resolve relationships between AGF was recently tested. The phylum *Neocallimastigomycota* was divided into four families, resulting in the prioritization of the D1/D2 LSU along with the protein-coding gene rpb1 for phylogenetic inference (Hanafy et al. 2023). As demonstrated by Hanafy et al. (2023), using multiple genes increased the phylogenetic resolution in *Neocallimastigomycota*. Therefore, developing new markers and curating their reference sequences provides valuable additional information for use in culture-independent surveys. Such studies could characterize community structure and diversity by screening a wide range of hosts and environments for new uncultured AGF genera (Hanafy et al. 2020b; Young et al. 2022), as well as tracing back the AGF coevolution in animal guts (Murphy et al. 2019).

Third‐generation sequencing technologies, such as MinION (Oxford Nanopore Technologies) and PacBio Sequel II (Pacific Biosciences), allow the analysis of long sequences, such as the complete *rrn* (Wurzbacher et al. 2019). This is particularly advantageous for deciphering complex genomic regions such as long repetitive elements or AT-rich segments of the AGF genetic regions (78 to 84%; Wang et al. 2019), the assembly of which using short reads (e.g., Illumina) can be highly problematic or even impossible.

In this study, we used MinION and PacBio sequencing to generate a comprehensive reference database of whole ribosomal operons and the adjacent intergenic region. This covered frequently used barcodes and previously unexplored genetic regions of the *Neocallimastigomycota*. Our aim was to examine the utility of the coding (SSU, 5.8S, LSU, and 5S) and non-coding (ITS1, ITS2, IGS, and ETS) regions for resolving AGF taxonomy, as well as identification of new potential markers within the *rrn* for culture-independent surveys. Additionally, with this comprehensive data, we aim to gather more information to elucidate the *Neocallimastigomycota* phylogeny, particularly with regard to the discussed *Cyllamyces* and *Caecomyces* genera.

## Materials and methods

### Culture and DNA collection

This analysis included pure cultures of AGF from twelve research institutions. The collection included at least one isolate from each *Neocallimastigomycota* genus that had been formally described by the end of 2022 (see Table 1, Suppl. material 1: supplementary data 1).

**Table 1.** Collection of *Neocallimastigomycota* isolates used for *rrn* barcoding. *Number of isolates used in this study. ** named according to Young et al. 2022. See Suppl. material 1: supplementary data 1 for the complete list of isolates and more details. Isolates in Bold indicate ex-type strains.

**Genus**	**Species**	**Isolate**	Amount*
* Aestipascuomyces *	*A. dupliciliberans*	**A252**	1
* Agriosomyces *	*A. longus*	**MS2**, MS4	2
* Aklioshbomyces *	*A. papillarum*	WT1, WT3, WT4	3
* Anaeromyces *	*A. contortus*	BRP041, G3C, G3E	3
	*A. mucronatus*	YoDo1, YoDo3, YoDo6	3
* Buwchfawromyces *	*B. eastonii*	**GE09**	1
* Caecomyces *	C. communis var. churrovis	Brit4, NZ17-B7, PP313, ViSuPo, YoDo26	5
	C. communis var. communis	BRP07182, OF1, TSB4	3
* Capellomyces *	*C. foraminis*	BGB12, BGS12, BGS2	3
* Cyllamyces *	*C. aberensis*	B32, TSB2	2
* Feramyces *	*F. austinii*	DF1, F2b, **F3a**, R4A, NZ11-SL-2-SI-20	5
* Ghazallomyces *	*G. constrictus*	ADS11, ADS12, AXS32	3
* Joblinomyces *	*J. apicalis*	GFH682	1
* Khoyollomyces *	*K. ramosus*	H1WF70NRF6, X2152, ZC41, ZS32, etc.	9
* Liebetanzomyces *	*L. polymorphus*	G6SC, ORC37	2
NC3	new genus-clade NC3	CF6e	1
* Neocallimastigales *	new genus-clade YL2 (Mara)	Mara4, Mara5	2
* Neocallimastix *	*N. cameroonii*	CaDo3a, YoDo11, YoDo12, YoDo13, etc.	5
	*N. frontalis*	DWFs31, EKC11, GFMa3.1, TSS3, YoRe34, NZ1-SF-4-CB-2, etc.	36
* Oontomyces *	*O. anksri*	B2, L1(YC), L2, L5, X1, X19, X23, X29, etc.	35
* Orpinomyces *	*O. joyonii*	D3B, D4C, L1(ME), W212	4
	new species-clade YL-X	NZ2-CF-4-CB-3	1
* Paucimyces *	*P. polynucleatus*	**BB3**	1
* Pecoramyces *	*P. ruminantium*	OS2, YC1, A8, **C1A**, SA222, YoRe27, etc.	9
* Piromyces *	*P. communis*	Jen.1, L10, **P**, UH3.1	4
	*P. cryptodigmaticus*	NZ30-BF2, NZ5-CF-3-CB-11, NZ9-CF-3-CB-17	3
	*P. finnis*	DonB21, H1	2
	*P. rhizinflatus*	CaDo1c, KiDo3e, NZ28-BF6	3
	*P.* sp. 6**	NZ10-CF-3-CB-19	1
	*P.* sp. 7**	A1	1
Cand. *Piromyces*	Cand. *P. potentiae*	B5	1
* Tahromyces *	*T. munnarensis*	BRP051	1
**Total**			156

### DNA extraction

DNA samples were extracted from pure and live cultures for most isolates. Frozen DNA samples were used for a few taxa for which no live culture is currently available (see Suppl. material 1: supplementary data 1: ‘Isolates table_Batch 1–5’). DNA extraction was performed in the laboratory in which the isolates were cultivated. Seven different DNA extraction methods were employed: the CTAB-method (Sirohi et al. 2013; Stabel et al. 2020); the DNeasy Plant Pro Kit (Qiagen, Hanafy et al. 2020b); the FastDNA SPIN kit for soil (MP Biomedicals, Young et al. 2018, 2022); the NucleoSpin Soil Kit (Macherey-Nagel, Düren, Germany); the repeated bead beating plus column method (RBB+C; Yu and Morrison 2004); the Plant/fungi DNA isolation kit (Norgen Biotek, Tophinke et al. 2022); and the PowerFecal DNA Isolation kit (MO BIO; Hooker et al. 2018). DNA extractions were performed in accordance with the respective suppliers' recommendations, unless otherwise noted in the above references. The extracted DNA samples were shipped to the Department of Laboratory Analytics at the Bavarian State Research Center for Agriculture (LfL) in Freising, Germany, where they were stored at –20 °C until further analysis.

### PCR amplification

The entire ribosomal operon (*rrn*) of the anaerobic fungal DNA extracts was barcoded in the first PCR using the adapter-linked primer pairs described by Wurzbacher et al. (2019). This generated three amplicons per isolate (Fig. 1): 1) The entire *rrn* operon (labelled FUN); 2) An amplicon encompassing most of the ribosomal genes (SSU, 5.8S and LSU), along with their respective non-coding regions (ITS 1 and ITS 2) (labelled shm); and 3) An amplicon of the IGS, including the 3’- end of the LSU, the 5’- end of the SSU and the 5S gene, along with the non-coding regions (ETS1, IGS1, IGS2, and ETS2) (labelled rc). The primers used were Fun-rOP-fwd (5'-CTGACTGTCTAATTAAAACAT-3') and Fun-rOP-rev (5'-TCAGATTCCCCTTGTCCGTA-3') for the whole ribosomal operon (FUN amplicon). To amplify the IGS amplicon the primers NS1rc (5'-GACAAGCATATGACTACTG-3') and RCA95rc (5'-CTGACTGTCTAATTAAAACATAG-3') were used (rc amplicon); as well as NS1short (5'-CAGTAGTCATATGCTTGTC-3') and RCA95m (5'-CTATGTTTTAATTAGACAGTCAG-3') for the shm amplicon (see detailed primer list in our Suppl. material 1: supplementary data 2_Index Primers).

We thawed the DNA samples slowly at 4 °C before proceeding with the PCR. The initial PCR reactions comprised 6 µL of PrimeSTAR GXL 5x buffer (Takara), 3 µL of dNTPs (2.5 mM for each nucleotide), 1 µL of each forward and reverse primer at a concentration of 10 pmol/µL, 1.2 µL of PrimeSTAR GLX polymerase (Takara), and 1 µL of DNA template at a concentration of > 0.1 ng/µL, with a total reaction volume of 30 µL made up with nuclease-free water. The PCR program for the rc and the shm amplicons consisted of an initial denaturation/activation step at 98 °C for 1 min, followed by 35 cycles of denaturation at 98 °C for 10 sec, annealing at 55 °C for 15 sec, and elongation at 68 °C for 2.5 min. The run ended with a hold phase at 4 °C. For the PCR of the whole *rrn* amplicon (FUN, up to 10 kbp), the elongation step was extended to 4 min. *Feramyces* isolate DF1 and nuclease-free water served as positive and negative amplification controls, respectively.

The three PCR products per isolate were diluted 1:100 and stored at 4 °C until required for the second PCR. In this PCR, the amplicons were barcoded using dual indexing (see Suppl. material 1: supplementary data 2: Index Primers). The second PCR program was identical to the first PCR, but comprised only 12 cycles and a 6-minute elongation step to barcode the amplicons individually using a dual index system (Mariz et al. 2025). To prevent carryover, the PCR cabinet was disinfected with 70% ethanol and ultraviolet light; furthermore, new sterile filter tips were used to prepare the reactions prior to each PCR. Gel electrophoresis was performed to evaluate the success of the PCR after each run. Amplicons from the second PCR were purified with 0.8% (v/v) AMPure magnetic beads (Beckman) following the manufacturer’s instructions. To create an equimolar pool, the concentration of each amplicon was determined using a Tecan Infinite 200 Pro M Plex Microplate Reader (Tecan Trading AG, Switzerland) and a Quant-iT™ PicoGreen™ dsDNA Assay Kit (Invitrogen by Thermo Fisher Scientific Inc.).

### Amplicon sequencing

The DNA extracts were divided into five library batches for long-read sequencing, primarily based on their arrival dates or putative PCR repeats. Each batch consisted of an equimolar pool of the three amplicons (FUN, shm, and rc) per isolate and was processed as an independent library (see Suppl. material 1: supplementary data 1: ‘Isolates table_Batch 1–5’). Each library was again purified using the AMPure magnetic beads protocol (Beckman) and concentrated to 50 µL. To optimize the sequencing quality, we used the newest technology available from Oxford Nanopore Technologies (ONT, nanoporetech.com) as soon as it was released. Thus, different flow cells and library preparation kits were used throughout the project (Table 2).

**Table 2.** Library preparation kits and flow cells used for each Batch.

**Batch Nr**.	**Sequencing technology**	**Flow cell**		**Library preparation kit**	**Basecaller**
1	ONT MinION	FLO-MIN106	R9.4	LSK 109	guppy, hac model
2	ONT MinION	FLO-MIN106	R9.4	LSK 109	guppy, hac model
3	PacBio RS II			SMRTbell Express	CCS
4	ONT MinION	FLO-MIN112	R10.4	Q20-EA	guppy, duplex model
5	ONT MinION	FLO-MIN114	R10.4	LSK 114	dorado, sup

Sequencing was performed using a MinION (ONT, nanoporetech.com). Batch 3 was sequenced using the PacBio RSII system (Pacific Biosciences). Briefly, Batch 3 was generated using the same procedure outlined above and was then used in the SMRTbell Express Template Prep 2.0 (PacBio). The library was quality-checked using a Fragment Analyzer (Agilent Technologies, high-sensitivity Large Fragment 50 kb Analysis Kit) and was subsequently sequenced on a PacBio Sequel II system.

### Data processing

**Figure 2.**
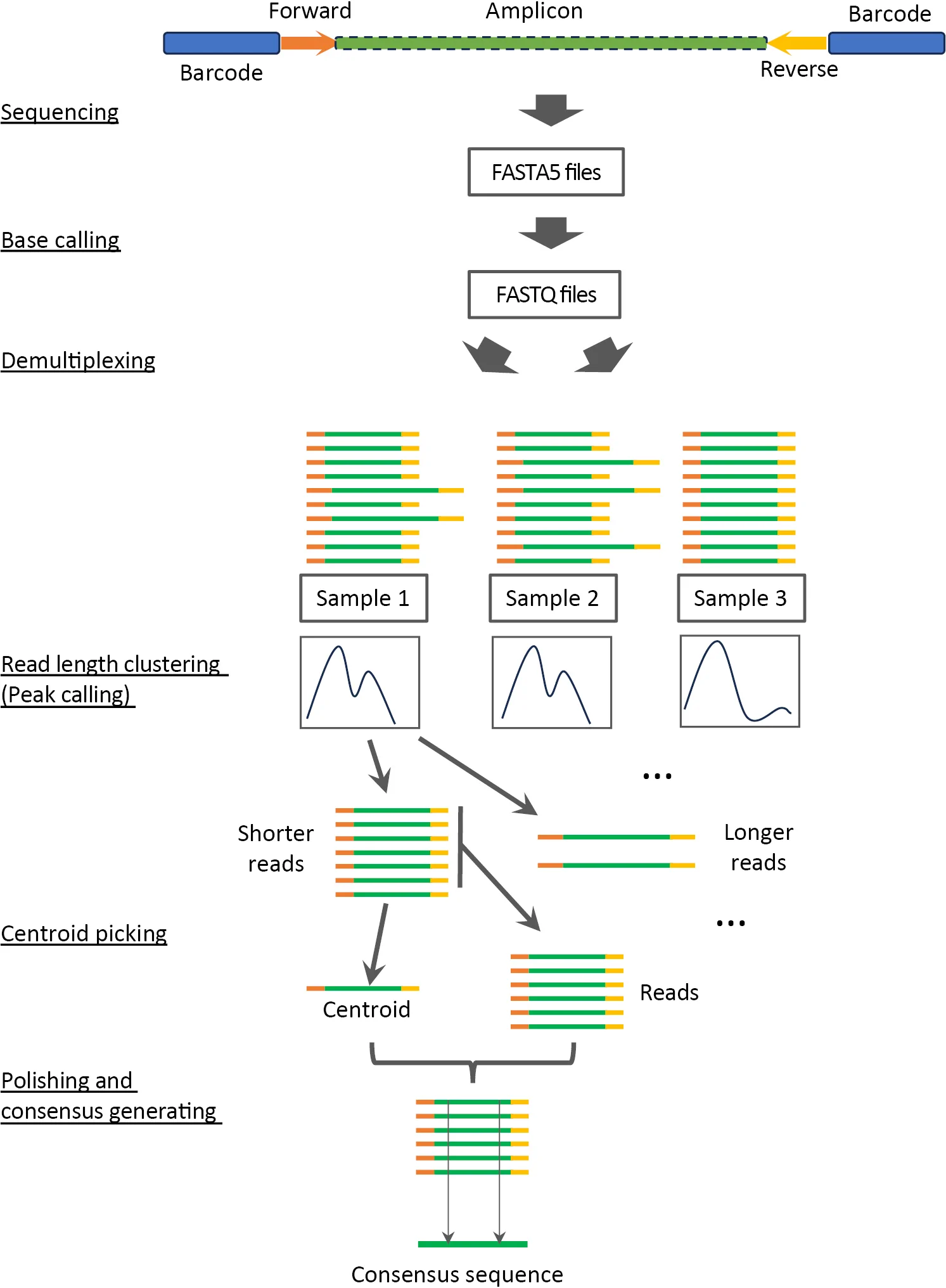
Customized workflow (longread_umi-AF-LSU, v 0.3.2) for the consensus generation of the *rrn* barcodes using reads from third-generation sequencers (ONT and PacBio).

The data processing pipeline was developed based on a customized version of the UMI pipeline from Karst et al. (2021) and the method used by Wurzbacher et al. (2019) for tandem repeat barcoding to generate reliable long-read consensus sequences (Fig. 2). The UMI pipeline was extended in our longread_umi pipeline (v. 0.3.2) with two new steps: 1) long-read pre-clustering (i.e. peak detection to discern length variability) and 2) a denoising step to achieve high-confidence consensus sequences. The pipeline and all scripts necessary to reproduce the analysis are available on GitHub (github.com/rhinempi/longread_umi-AF-LSU).

#### 1. Long-read processing

All five batches of long-read sequences from the Oxford Nanopore MinION and PacBio sequencers were first demultiplexed using the barcode and primer designated to each sample. Minimap2 v. 2.18 (Li 2018) was then used to map each long-read sequence to the barcode-primer strings. The following input parameters were used: -t 20, -k7, -A1, -m42, -w1, and -Nx. The -Nx parameter was set based on the number of samples that shared the same barcode-primer string. The -k parameter was set to 7 because the primer-barcode strings were relatively short (47–51 bp). After demultiplexing, two 18 bp sequences (forward and reverse) were extracted from the primer-barcode strings of each sample. These sequences served as placeholders for the unique molecular identifiers (UMIs) in the pipeline. The UMI sequences then served as anchors to align and cluster the long-read sequences further, identifying minor misclassified reads from the previous demultiplexing step, and served as the basis for denoising the sequencing data using data filtering steps (Karst et al. 2021).

#### 2. Peak detection

If not accounted for, variations in amplicon length will introduce challenges in consensus calling due to compromises made between long and short amplicons, such as insertions and deletions. Thus, the pipeline distinguishes intragenomic *rrn* variants of different lengths by identifying density maxima (see Fig. 2, Suppl. material 1: supplementary data 3: Peak detection). The length cutoffs are set based on the expected length of three different amplicons: FUN amplicons are set to a length cutoff between 6000 nt to 10000 nt, whereas shm and rc amplicons are set to 1000 to 6000 nt. Peak calling was carried out using R. The density distribution of the reads was calculated using the “density” package. The resulting distribution was then used to detect peaks and their range with the “peaks” function in R. The sequences comprising the density maxima (i.e. the peaks) were subsequently processed in parallel.

#### 3. Denoising

All reads were clustered by pairwise identities with a 75% threshold (Karst et al. 2021), which accounts for the higher error rates in long-read sequencing reads (up to a 15% error rate for the Oxford Nanopore MinION sequencer; Delahaye and Nicolas 2021). The centroid sequence of each cluster was selected based on abundance and used as a reference template. All other sequences within each cluster were then used to polish the centroid with Racon v1.4.10 (github.com/isovic/racon), producing the final consensus sequences for the PacBio datasets. For the Oxford Nanopore MinION sequences, Medaka polishing v.1.4.3 (github.com/nanoporetech/medaka) was applied after Racon polishing to produce high-quality consensus sequences. The minimum cutoff for retaining a consensus sequence was set to 10. Clustering was verified by comparing each resulting variant to Sanger sequencing data (spanning ITS and LSU) generated from the respective isolates. We compared our customized pipeline v0.3.2 to other tools to verify its clustering accuracy.

#### 4. Variant & SNP detection

We used Longshot v1.0.0 (Edge and Bansal 2019) to detect variants and single-nucleotide polymorphisms (SNPs) after generating consensus sequences. Longshot is designed to address the higher error rates of long-read sequencing technologies by utilizing haplotype information in long-read sequences to accurately detect SNPs. To ensure high-confidence SNP calling, the occurrence of SNPs generated by Longshot in samples from the same species was manually verified.

### Multiple sequence alignment

The identity of the obtained shm, rc, and FUN consensus sequences was confirmed individually by a nucleotide BLAST search against public databases of the International Nucleotide Sequence Database Collaboration (www.insdc.org/) and the JGI MycoCosm database (mycocosm.jgi.doe.gov/mycocosm/home). Close relatives were downloaded and used as reference sequences/outgroups to generate a multiple sequence alignment file for downstream analysis. We aligned all consensus sequences and close relatives in SEED 2 (Větrovský et al. 2018) using MAFFT v. 6.240 with the default settings. This resulted in a preliminary global alignment. We further refined the alignment for the highly variable segments IGS, ETS, and ITS segments by manually co-arranging semi-conserved motifs (20–80% conserved), as identified by internal BLAST searches. No positions were excluded during this procedure. The final *rrn* alignment included the FUN consensus sequences and the concatenated shm and rc consensus sequences, which cover all ribosomal coding and non-coding genetic regions (Fig. 1). The multiple sequence alignment is available in Suppl. material 1: supplementary data 8 (Full Alignment) and at the Anaerobic Fungi Network website (https://anaerobicfungi.org).

### Phylogenetic analysis

The consensus sequences from each of the amplicons (FUN, rc, and shm) were analyzed to identify their respective variability and phylogeny. Multiple sequence alignments were used to construct genetic region-specific phylogenetic trees with FastTree 2.1.11 (Price et al. 2010), applying cat 20 as input parameter in Geneious Prime (v. 2024.0.5). The phylogenetic trees were generated using maximum likelihood and the Jukes–Cantor model. FastTree computed 1,000 bootstrap replicates and tested alternative tree topologies using the Shimodaira–Hasegawa (SH) test, as described in Price et al. (2010). The outgroups consisted of *Malassezia globosa* (a yeast-like *Basidiomycota* species) and *Powellomyces hirtus* (a *Chytridiomycota* species). Species names were first assigned according to the nomenclature provided by the strain owners, then according to Young et al. (2022), especially for the *Piromyces* clade. We applied format modifications to the phylogenetic trees using the ARB_NT v. 7.0 software (http://www.arb-home.de/home.html; Ludwig et al. 2004).

For the phylogenetic tree of the entire ribosomal region *rrn* and the IGS, we applied an alignment filter using trimAI (v. 1.5) (Capella-Gutierrez et al. 2009) with gap threshold of 0.3 and a similarity threshold of 0.001 (as previously applied for long ribosomal reads (Radek et al. 2023). The tree was constructed with IQ-TREE (v. 3.1.3) (Wong et al. 2026) using 1000 ultrafast bootstrap replicates with bnni and 1000 aLRT replicates on a region partitioned alignment using ModelFinder (Kalyaanamoorthy et al. 2017). A sensitivity analysis was done to analyze the effect of the IGS regions and IGS1 was removed from the alignment subsequentially (see Supplemental Data 14).

### Quality control

The automatic MAFFT alignment was locally ambiguous and implausible, so it was manually refined. Through visual inspection, the incomplete consensus sequences were discarded first. Then, phylogenetic trees were generated with the remaining sequences and references to identify spurious sequences, where possible. Sequences located between clades were scrutinized through pairwise sectoral alignments and BLASTn-based analyses of suspect regions. Implausible sequences identified as chimeras were discarded (see Suppl. material 1: supplementary data 4: Pipeline sorting). Finally, 120 consensus variants that were incomplete or unreliably arranged and for which no phylogenetic plausibility was found were discarded. Removing these sequences reduced the risk of including artifacts. The remaining reliable consensus sequences were submitted to Genbank through NCBI under the accession numbers: PZ357937–PZ358123, PZ358188–PZ358439, PZ358440–PZ358523, PX908483–PX908697, and PX897217–PX897271. The concatenated *rrn* sequences of the most abundant corresponding shm and rc consensus amplicons can be found in the multiple alignment file (Suppl. material 1: supplementary data 8-Full Alignment).

### Ribosomal operon variants

For some species and even some strains, multiple consensus variants were obtained. The consensus sequence with the highest count, or the dominant genomic variant, served as the species or strain reference. The consensus variants were examined separately for their phylogenetic position. Spurious variants with a considerably lower abundance than the dominant variant of a given strain (fewer than 10 reads) were excluded if they were affiliated with another taxonomic rank.

### Statistics with ribosomal coding and non-coding regions

We defined the start and end points of each region using the respective databank references for SSU, LSU, ITS, 5.8S, and 5S (Suppl. material 1: supplementary data 14). However, since there are no close references available for the ETS region, we searched for conserved start and stop codons in the alignment. For the start of ETS1, we identified several putative transcription start sites at the following positions with reference to the FUN-concatenated alignment: E1 (alignment position 6433), E2 (alignment position 6618), E3 (alignment position 6712). For the results presented below, we used E1 as the putative start of the ETS1 (Suppl. material 1: supplementary data 14). Geneious Prime (version 2024.0.5) was used to calculate the distance matrix (Jukes-Cantor distance), the coefficient of variation (CV), and the standard deviation (SD) of the three amplicons (FUN, shm, and rc) and the genetic markers inside the *rrn* operon analysed in this study, according to the number of reads for each isolate, species, and genus.

The distance matrix was used to examine closely related clades in more detail, such as *Cyllamyces* and *Caecomyces*. We used a two-tailed Pearson correlation test with a 95% confidence level using the R package stats v. 3.6.2 to assess the relationship between sequence variation and AT content. A standard deviation analysis evaluated the similarity within operon markers and genera/species. The CV showed the relative measure of variability, indicating the size of a standard deviation relative to its mean and representing the percentage of samples that fall within one standard deviation. Low standard deviation (<0.35) indicates low sequence variance. A high coefficient of variation (≥99.5) showed low sequence similarity. A two-way ANOVA analysis was performed using GraphPad Prism version 10.2.3 for Windows (GraphPad Prism Software, Boston, MA, USA). This analysis considered the taxonomic level and the genetic region as factors and the sequence variance as the dependent variable. Tukey's multiple comparison test was then used to identify statistical differences (applying an alpha of 0.05; degrees of freedom 8060; F values of interaction 217.3, row factor of 3233 and column factor of 1298) between the genetic regions. The measurements were taken from distinct samples.

## Results and discussion

### A comprehensive database for the *rrn* operon in the *Neocallimastigomycota*

Through an international collaboration with researchers from Germany, the USA, New Zealand, India, China, the Czech Republic, the UK, and Austria, we obtained a comprehensive collection of anaerobic gut fungal DNA from 156 isolates (Table 1, Suppl. material 1: supplementary data 1). These isolates belonged to 31 species, with at least one isolate of each of the 20 genera described by 2022. Two genera described by Pratt et al. (2023) and Morris et al. (2026) were not accessible at the time of sequencing. The collection also included two newly discovered genus-level clades (YL2 and NC3, Young et al. 2022).

Long-read sequencing of the complete ribosomal (*rrn*) operon (FUN) and its two complementary amplicons (shm and rc; Fig. 1) from 156 isolates yielded 770 consensus sequences (see Suppl. material 1: supplementary data 4: Pipeline sorting). At least one high-quality full-length *rrn* consensus sequence (either FUN or concatenated shm and rc) per genus was generated (as examined against previously used consensus generation tools and Sanger sequences, see Suppl. material 1: supplementary data 5a, b). The only exception was *Tahromyces*, for which only the rc amplicon could be successfully sequenced. In addition to the 156 isolates, 39 additional isolates were not amplifiable, most likely due to a low DNA concentration (as low as < 0.2 ng/µL), old DNA stock (from as early as 1994), prolonged thawing during international transport, and/or high DNA fragmentation. This was not critical except for *Tahromyces*, since those extra 39 isolates belonged to species for which alternative isolates or DNA samples were available in the study. Amplicon length was one crucial factor for a successful amplification most likely due to their susceptibility to DNA quality and fragmentation during extraction and further manipulation.

### Variability within the *rrn* operon

The mean length of a single *rrn* operon copy of the *Neocallimastigomycota* was ~9 kbp, averaged over 187 consensus sequences (79 isolates, accounting for 51% of the total isolates sampled; Table 3). The shm fragment, which contains nearly the entire SSU, the ITS1, 5.8S, ITS2, and two-thirds of the LSU length (spanning the divergent regions D1 to D8), had an average length of 4.7 kbp, as determined from 259 consensus sequences (151 isolates, accounting for 97% of the total isolates sampled). The rc amplicon (3’ end of the LSU, ETS2, IGS1, 5S, IGS2, and ETS1) had an average length of 4.5 kbp from 324 consensus sequences (136 isolates, 88% of the total isolates sampled). The rc amplicon exhibited the greatest length variability and nucleotide heterogeneity (Table 3).

**Table 3.** Characterization of each amplicon. Resulting from the analysis of 770 reliable consensus sequences. bp: base pairs without gaps. CS: Consensus sequence(s).

**Amplicons**	**FUN**	**Shm**	**rc**
**Shortest CS**	8,140 bp	4,650 bp	3,540 bp
**Longest CS**	9,950 bp	4,750 bp	5,440 bp
**Average length CS (±SD)**	9,045 bp (±908)	4,700 bp (±50)	4,490 bp (±950)
**Isolates successfully sequenced**	79	151	136
**Successful CS obtained (n)**	187	259	324

In addition to comparing these three amplicons, we explored the genetic regions they included (Table 4). These regions exhibited distinct lengths and sequence variabilities. The IGS region exhibited the longest extent with the entire length differing by at least one-third. The most conserved length was observed for the 5.8S with 158 ± 1 bp (Table 4). The length variabilities of ITS2, ITS1, and ETS2 reached 14.3%, 17.5%, and 18.9%, respectively. This poses challenges for alignments, and consequently may lead to inconsistencies in phylogenetic analysis, as previously reported by e.g., Edwards et al. (2017). The other genetic markers (SSU, LSU, 5S, and the non-coding ETS1) exhibited low length variability, ranging from 10 to 90 bp (0.6% to 6.5% length variation). The transcribed loci of all AGF contained more conserved fragments, facilitating the creation of unambiguous multiple sequence alignments.

**Table 4.** Length variability for each genetic marker in the whole ribosomal operon. All values are in bp (base pairs). CS: Consensus sequence.

**Genetic marker**	**ETS1**	**SSU**	**ITS1**	**5.8S**	**ITS2**	**LSU**	**ETS2**	**IGS1**	**5S**	**IGS2**
**Shortest CS**	814	1,790	193	157	180	3,390	47	1,030	111	104
**Longest CS**	929	1,810	279	159	240	3,570	70	2,150	120	1,335
**Average length CS**	871	1,800	246	158	210	3,480	58	1,590	115	719
**(±SD)**	(±57)	(±10)	(±43)	(±1)	(±30)	(±90)	(±11)	(±560)	(±5)	(±616)

### Number of *rrn* operon sequence variants

**Figure 3.**
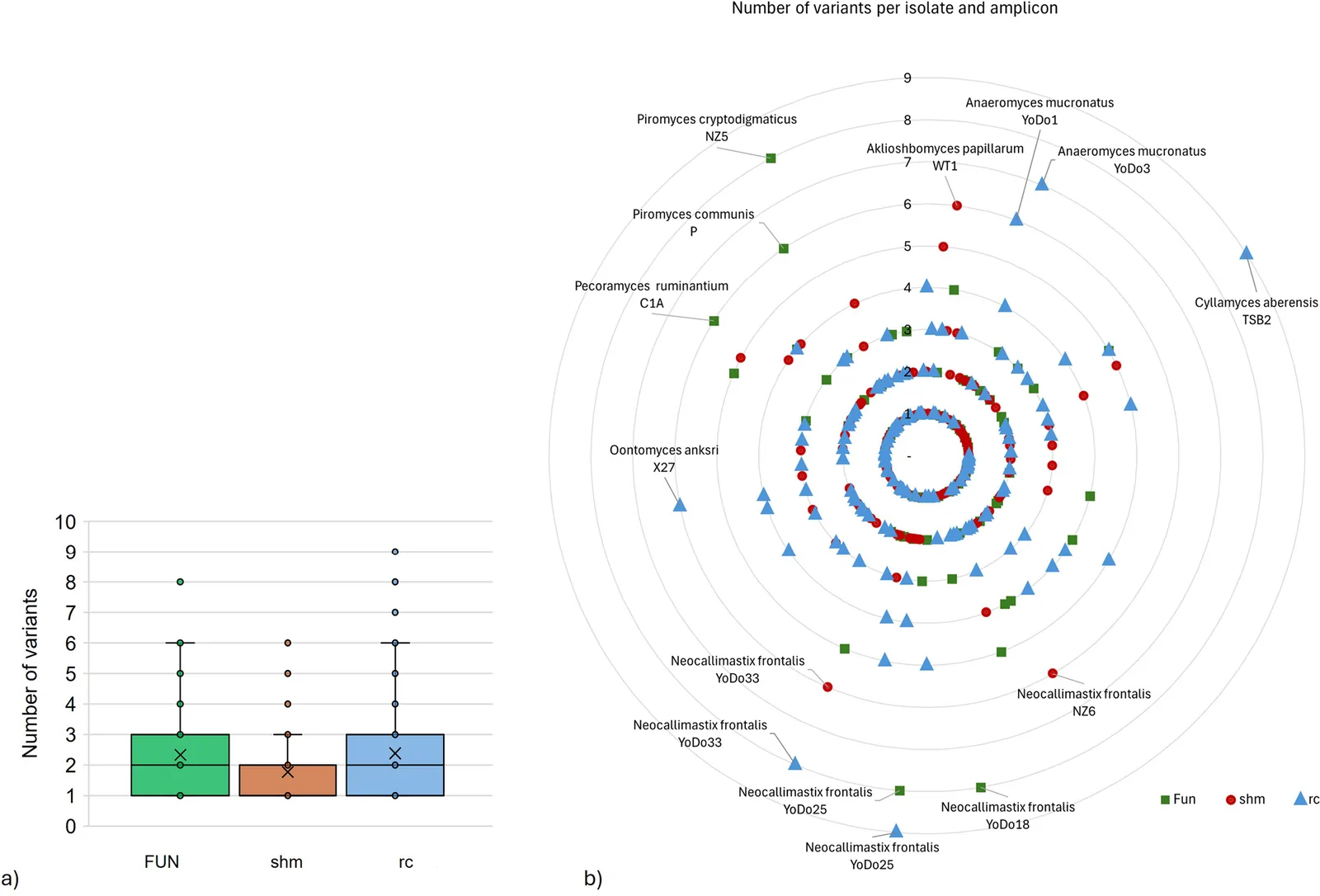
Distribution of the number of consensus sequence variants per isolate and amplicon: FUN, shm, rc. **a**. Boxplot summarizing the number of variants per amplicon with standard deviation and mean (represented by an “x”). **b**. Radial plot detailing which isolates had the highest number (≥ 6) of consensus variants per amplicon. The total number of consensus sequences is as follows: n = 187 for FUN, n = 259 for shm and n = 324 for rc. Green squares: FUN, red circles: shm, and blue triangles: rc. For more details, see Suppl. material 1: supplementary data 6: Ribosomal operon variants per isolate.

Sequencing with MinION and PacBio allowed us to accurately determine the number of *rrn* variants per AGF isolate. Generally, most isolates presented only one or two *rrn* sequence variants (Fig. 3a). We observed the presence of one or two consensus sequence (CS) variants per isolate in 65% (88/136 CS) of the rc, 69% (55/80 CS) of the FUN, and 84% (123/146 CS) of the shm amplicons. However, some outliers with six to nine CS variants were observed (Fig. 3a, Suppl. material 1: supplementary data 6: Ribosomal operon variants per isolate). Only 6.3% of the isolates (5/80 CS) presented a high number of variants (≥ 6 CS) in the FUN amplicon, 4.4% in the rc amplicon (6/136 CS), and only 2.1% in the shm amplicon (3/146 CS). The mean number of sequence variants was higher in the rc amplicon and consequently in the whole tandem repeat (FUN) compared to the shm amplicon. This pattern stems from the fact that most of the variations originate from the intergenic region (IGS) in the rc amplicon. This is due to its variable length (Table 4), as a result to their numerous repetitive AT motifs (see section below).

AGF isolates with more than six *rrn* consensus variants belonged to the following species: *Neocallimastix frontalis*, *Anaeromyces mucronatus*, *Cyllamyces aberensis*, *Aklioshbomyces papillarum*, *Pecoramyces ruminantium*, *Piromyces communis*, *Piromyces cryptodigmaticus*, and *Oontomyces anksri* (Fig. 3b). There was no clear association between the number of consensus sequence variants per isolate and AGF anatomy, including morphological characteristics such as monocentric versus polycentric, bulbous versus rhizoidal, and monoflagellated versus polyflagellated types (see Suppl. material 1: supplementary data 7: Summary AGF morphology and *rrn*). Interestingly, it seems that the number of *rrn* sequence variants can differ between strains of the same species. For example, four out of 36 examined *N. frontalis* isolates presented > 6 consensus variants; however, all other *N. frontalis* strains exhibited a statistical mode of 1–2 consensus variants. The same case was observed for *A. mucronatus*, the genus *Piromyces*, and the other outliers in Fig. 3b, where only a few of their isolates had more than six consensus variants.

### Quantifying sequence variability in the *rrn* operon

**Figure 4.**
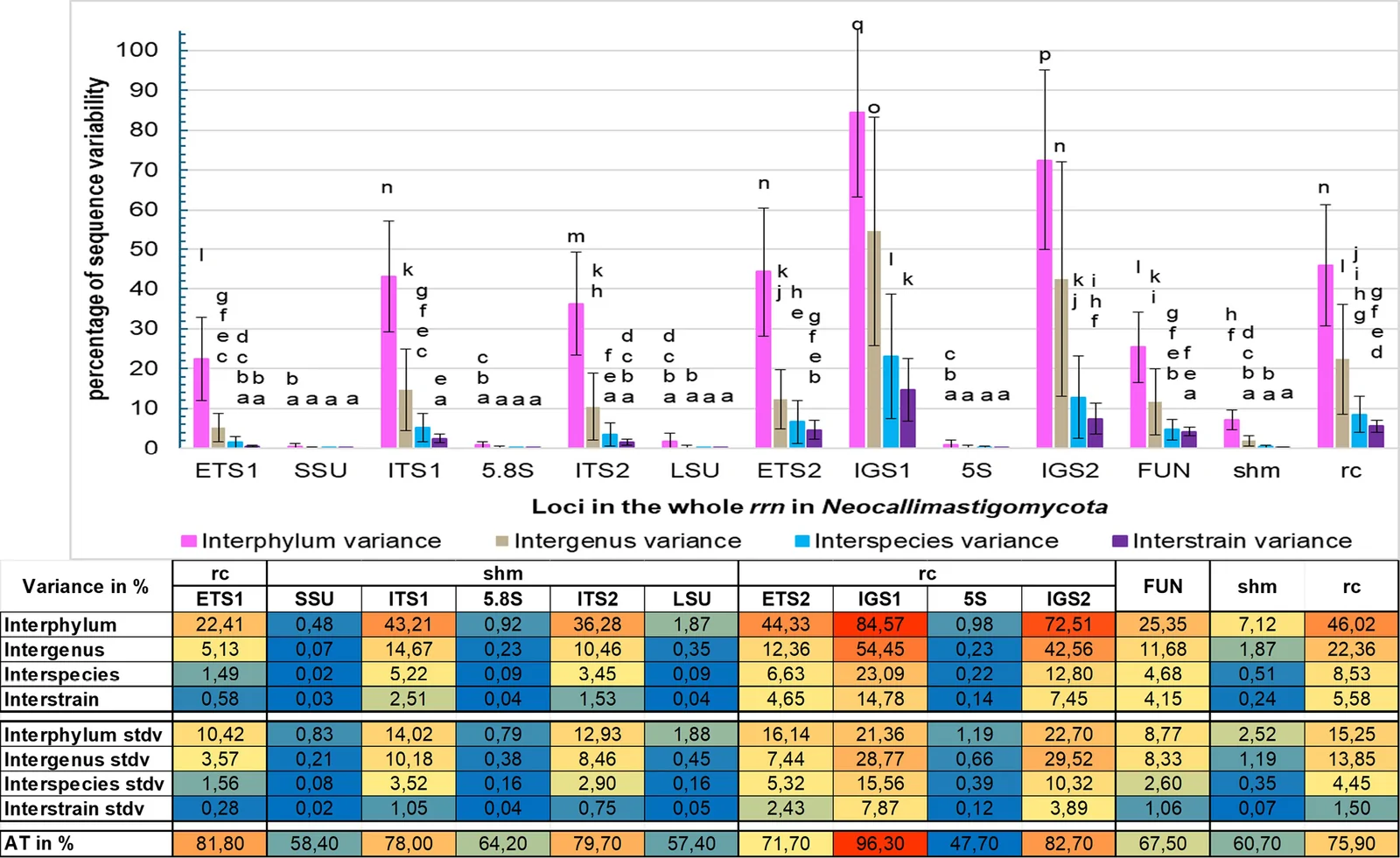
Significance of the sequence variance within the phylum, genera, species, and strains (bars appear from left to right in that order) in each *rrn* region, the whole *rrn* operon (FUN), and the shm and rc amplicons. Identical letters depict no significant difference. The heatmap highlights low variance and standard deviation (SD) values in blue, and high values in red. A representation of the whole *rrn* operon can be seen in the Suppl. material 1: supplementary data 9. n = 511 for ETS2, IGS1, 5S, IGS2 and ETS1; n = 762 for SSU and LSU; n = 434 for ITS1, 5.8S, and ITS2.

Analyzing sequence divergence per genetic region at the different taxonomic levels (phylum, genus, species, and strain) within *Neocallimastigomycota* revealed that the IGS1 exhibited the highest variability, followed in decreasing order by IGS2, ETS2, ITS1, ITS2, ETS1, LSU, 5S, 5.8S, and SSU (Fig. 4).

The entire *rrn* operon exhibited a sequence variability of 25.4% (SD 8.8) across the whole phylum, within genera of 11.7% (SD 8.33), within species of 4.7% (SD 2.6) and within variants 4.2% (SD 1.1). These *rrn* operon values derive from the high variability of the rc, while lower variability was present in the shm amplicon (Fig. 4). The high variability in the rc amplicon, and consequently in the whole *rrn*, was due to the high sequence variability of the IGS1 and IGS2 (Fig. 4), with extremely high variabilities within the phylum of 84.6% and 72.5% (SD 21.4 and 22.1), respectively.

The lowest sequence variability was observed in the SSU with 0.48% (SD 0.83) across the entire phylum, 0.07% (SD 0.21) across genera, 0.02% (SD 0.08) across species and 0.03% (SD 0.02) across strains (Fig. 4). This indicates that the SSU is an extremely conserved gene region. The genetic regions most commonly used as phylogenetic markers (LSU and ITS) exhibited different variances. The LSU had low values of 1.87% (SD 1.88) within the phylum, 0.35% (SD 0.45) within genera, 0.09% (SD 0.16) within species and 0.04% (SD 0.05) within strains. Although there were no significant differences between LSU and SSU, the LSU is much more phylogenetically informative. ITS1 and ITS2 have much higher sequence variability than LSU, with an average of 40% within the phylum, 13% within genera, 4% within species, and 2% within strains. These variabilities are also found in the transcribed non-coding ETS regions (Fig. 4, see Suppl. material 1: supplementary data 8 for more details on the variation of all genetic regions).

On average, the ITS1 of all our samples showed 5.22% interspecies variance. However, there were key exceptions. The highest level of ITS1 variability in consensus variants from a single strain was observed in the Caecomyces communis var. communis isolate TSB4 isolate, with a mean intragenomic variation of 24.9%. This value was much higher than the mean for all other strains combined (2.28%), excluding the TSB4 isolate. C. communis var. communis isolate TSB4 also showed the highest strain intragenomic mean variation of 11.9% in the ITS2 locus. Other isolates with high intragenomic strain variation in ITS1 were *Anaeromyces contortus* BRP041 (16.5%) and *Orpinomyces joyonii* D3B (11.3%), both of which had three sequence variants. Callaghan et al. (2015) also reported high intragenomic variation between clones of *Buwchfawromyces eastonii*, with up to 13% variation within 200 bp of the ITS1 region. In our analysis, DNA from the same isolate (GE09) only exhibited a 6.94% ITS1 strain intragenomic variation in the two shm consensus variants generated.

Both 5.8S and 5S exhibited low sequence and length variability (Fig. 4, Table 4), indicating that they are highly conserved genetic regions. However, the 5S displayed interesting structural characteristics. With two exceptions of low read-count (11 and 16 reads), for which no 5S was present, the 5S was found in the sense or antisense direction. It occurred in inverted orientations in 27.9% of all the examined consensus sequences. The inverted 5S gene was observed in seven out of the 20 genera and two new genus-level clades analyzed in this study: *Buwchfawromyces*, *Oontomyces*, *Orpinomyces*, *Piromyces*, and *Tahromyces*, as well as the new genus-level clades NC3 and YL2. Inversion of the 5S gene has also been detected in several other eukaryotic organisms, including the tilapia fish *Oreochromis niloticus* (Martins et al. 2002) and fungal genera such as *Saccharomyces* and *Coprinopsis* (Cassidy and Pukkila 1987) as well as various genera from the phylum *Oomycota* (Belkhiri et al. 1992). Our observations indicate that the morphological characteristics of the analyzed AGF isolates are not related to the inversion of the 5S gene. However, in the case of *Piromyces*, this characteristic could be used to distinguish *Piromyces* from other AGF with the similar morphological traits (see Suppl. material 1: supplementary data 10 for more details). Conversely, we observed both variants (inverted and sense-oriented 5S) in two of the four *Orpinomyces joyonii* strains, indicating intergenic variation between the ribosomal repeats of the same organism. To evaluate whether either would be transcribed, we examined the presence and location of TATA-like boxes and found that both orientations might be transcribed. The TATA-like boxes were mainly located downstream in 13 genera and at both ends in six genera (see Suppl. material 1: supplementary data 10 for more details).

Contrasting variability between the genetic regions is directly affected by the AT/GC proportion in each sequence (Fig. 4). The AT content of the ribosomal operons varied moderately among the functional genes (SSU, 5.8S, and LSU,), ranging from 58.4% to 64.2%. Conversely, the non-coding transcribed regions (ITS and ETS) exhibited a higher AT content ranging from 71.7% to 81.8%. The high AT percentage in the transcribed spacer regions is mainly represented by multiple repetitions of ~25 bp AT motifs. Nicholson et al. (2005) and Wilken et al. (2020) also noted the high AT-rich genomes of AGF. Youssef et al. (2013) reported an even higher AT content of 83% in the complete genome of *Pecoramyces ruminantium* C1A isolate. The average AT content of the whole *rrn* operon was 65.6% in the six C1A consensus variants sequenced in our study. Pearson’s test revealed a significant correlation between the sequence variability and AT content throughout the entire *rrn* operon (r = 0.85, p < 0.001). However, no significant correlation was observed between AT content and sequence variability for ETS2, 5S and 5.8S. The non-transcribed and non-functional loci (IGS1 and IGS2) exhibited the greatest sequence variability, primarily consisting of AT-motifs with an average AT content of 90%. Therefore, it is evident that these non-functional/non-transcribed regions exhibit a low sequence conservation because they are not under selective pressure of conserving a function linked to gene transcription.

### Phylogenetic analysis of the *Neocallimastigomycota* using the *rrn* operon

**Figure 5.**
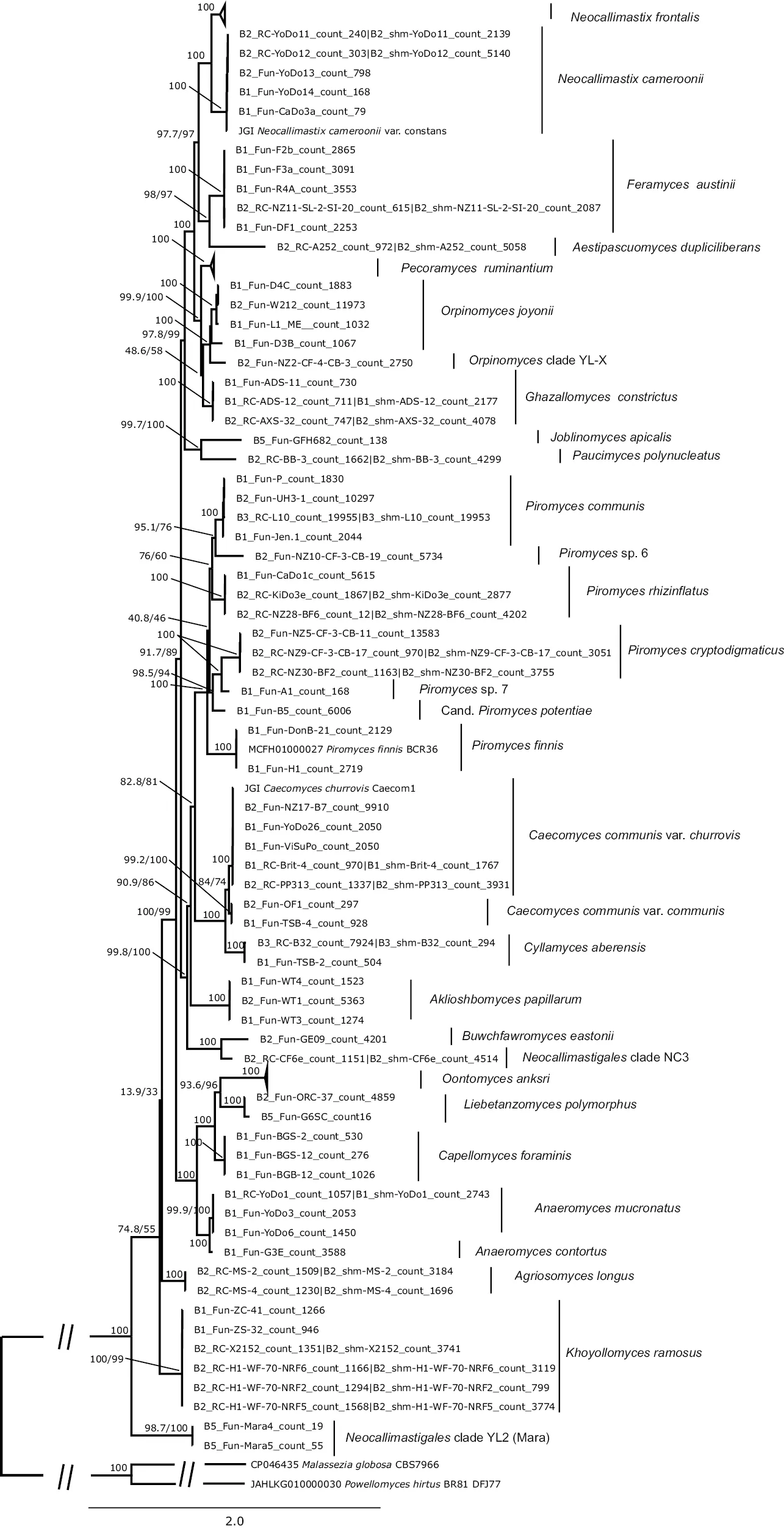
Phylogenetic tree inferred with the *rrn* (FUN amplicon) consensus sequences and the concatenated *rrn* (rc and shm amplicons joint) without IGS1. The percentages at the nodes represent support values for the SH-like approximate likelihood ratio test and the Ultrafast bootstrap, respectively (100 indicates full support for both values). Intraspecies bootstrap values have been removed and the branch leading to the outgroup has been shortened for visualization purposes; otherwise, branch lengths are proportional to substitutions per site. A high-resolution phylogenetic tree can be found in Suppl. material 1: supplementary data 11a.

To evaluate the entire ribosomal operon for AGF phylogenetics, we constructed a phylogenetic tree with all full-length consensus sequences (FUN amplicons and concatenated rc and shm variants filtering out the IGS1 as described in the methods section). All the genera examined, including *Piromyces* with seven species included in this study, as well as *Cyllamyces* and *Caecomyces*, and its varieties C. communis var. churrovis and C. communis var. communis, formed well-supported clades with high bootstrap values; 100 within the species-level clades of the genus *Piromyces*, 100 for the *Cyllamyces*/*Caecomyces* clade, and 84 for the *Caecomyces* varieties (Fig. 5, Suppl. material 1: supplementary data 11a). Even when some clades were less supported, e.g., 48.6% for the *Orpinomyces*/*Ghazallomyces* clade, the clear phylogenetic segregation of all the analysed species asserts that the whole ribosomal region (excluding IGS1) is a highly informative marker to delineate AGF.

The phylogenetic resolution provided by each amplicon was further assessed by constructing phylogenetic trees for the individual shm and rc amplicons (Suppl. material 1: supplementary data 11c–e: Phylogenetic trees). All examined genera were clearly differentiated with substantial support in those trees. For the *Cyllamyces*/*Caecomyces* clade, the shm amplicon correctly separated these genera. However, *Cyllamyces* and *Caecomyces* sequences were intermingled on the rc phylogenetic tree. Surprisingly, though, the rc amplicon presented an outstanding separation of all other genera, despite its high sequence variability due to its non-coding loci (IGS1, IGS 2 and ETS2), as well as its high AT content of approximately 76% (Fig. 4). Thus, the rc phylogenetic resolution is mainly attributed to the ETS1 locus (see Section Identification of new markers in the *Neocallimastigomycota rrn* operon). ETS1 provided an unambiguous resolution of *Cyllamyces* and *Caecomyces* (see Suppl. material 1: supplementary data 11f).

To evaluate the influence of the intergenic spacer regions on phylogenetic inference, sensitivity analyses were performed by constructing phylogenetic trees using the entire ribosomal operon alignment (Suppl. material 1: supplementary data 11b) and alignments excluding IGS1, IGS2, or both regions (see methods and Suppl. material 1: supplementary data 14 for more details). Excluding IGS1 after filtering had only a minor effect on the inferred topology, whereas exclusion of IGS2 or of both regions resulted in larger topological changes. Despite pronounced rate and compositional heterogeneity associated with IGS2, the principal backbone remained stable across all phylogenetic trees, while branch support decreased with the removal of IGS2 sites, and thus provided substantial phylogenetic resolution for the phylogenetic tree covering the entire ribosomal region (Fig. 5) despite its heterogeneous evolutionary behavior.

The evaluation of individual phylogenetic trees showed the utility of each genetic region in resolving the overall phylogenetic relationships within the *Neocallimastigomycota* and determining their phylogenetic resolution. The LSU gene provided the highest phylogenetic resolution among all the *rrn* regions (see Suppl. material 1: supplementary data 11h), showing the best clade structure without unexpected paraphyletic or polyphyletic groupings. Caecomyces communis var. churrovis forms a distinct clade; however, *Cyllamyces* and Caecomyces communis var. communis are intermixed within a single monophyletic clade. The LSU gene also displayed strong resolution among the seven *Piromyces* species included in this analysis, which was generally supported by high bootstrap values.

The lowest phylogenetic resolution was observed in the coding genes 5S and 5.8S, which lacked clear genus-level separation and formed only six and eight clades, respectively (see Suppl. material 1: supplementary data 11i, j). For instance, the genus *Piromyces* was scattered across several clades. The ETS2 phylogeny also showed very low resolution, with *Neocallimastix*, *Piromyces*, and *Pecoramyces* distributed polyphyletically (see Suppl. material 1: supplementary data 11k). IGS1 and IGS2 displayed nearly identical phylogenetic resolution, with mainly *Orpinomyces*, *Neocallimastix*, and *Piromyces* scattered across different clades (see Suppl. material 1: supplementary data 11l, m). Hausner et al. (2000) reported considerable IGS variability among *Piromyces* strains, suggesting that IGS could serve as a phylogenetic marker for this genus. In our study, however, IGS1 and IGS2 showed *Piromyces* to be polyphyletic, assigning *Piromyces* sp. 6 (isolate NZ-10-CF-3-CB-19) to the *Aestipascuomyces* clade. In contrast, ETS1 and LSU provided better resolution for the genus *Piromyces*, with all species forming a monophyletic clade. Our results demonstrate that the phylogenetic trees inferred using 5S, 5.8S, ETS2, or IGS regions individually, do not provide reliable resolution.

Even when the SSU was phylogenetically informative at the genus level, it did not provide enough resolution to delineate species within the *Orpinomyces*, *Piromyces*, and *Caecomyces* genera (see Suppl. material 1: supplementary data 11q). ITS is a universal fungal barcode and has also been used for AGF phylogeny in the past, but the phylogenetic inference generated by ITS is disputable for AGF (Suppl. material 1: supplementary data 11p). ITS placed *Neocallimastix* species in paraphyletic clades, separating *N. frontalis* from *N. cameroonii*, as seen in the ITS1 phylogenetic tree in Hanafy et al. (2023). Additionally, neither ITS1 nor ITS2 provided good inference in the *Piromyces* clade or for *Cyllamyces* and *Caecomyces* (see Suppl. material 1: supplementary data 11n, 11o). ITS2 even presented *N. frontalis* as a paraphyletic clade and the new *Orpinomyces* clade YL-X as polyphyletic. However, ITS2 could separate C. communis var. communis, but not *Cyllamyces* and C. communis var. churrovis. After analysing each genetic region separately, we can confirm that the phylogenetic resolution of the LSU and ETS1 might be the regions that improve the whole *rrn* operon use as a molecular marker.

### The case of *Caecomyces* and *Cyllamyces*

The observation that amplicons from *Caecomyces* and *Cyllamyces* appeared intermixed in multiple phylogenetic analyses in our study and in previous research (Dollhofer et al. 2016; Hanafy et al. 2022, 2023; Young et al. 2022) suggests that *rrn* sequences may not always align with species or genus thresholds. James et al. (2006b) used the primers SR1R and LR12 from Vilgalys and Hester (1990) to obtain most of the ribosomal RNA operon (18S, 5.8S, and 28S subunits), which is 4.1 kbp long. Their resulting phylogenetic tree shows six anaerobic fungal isolates as sister taxa to the *Chytridiomycota*. *Cyllamyces* and *Caecomyces* shared a clade despite their morphological differences with polycentric thalli in *Cyllamyces* and monocentric thalli in *Caecomyces* (Gold et al. 1988; Ozkose et al. 2001).

Almost 20 years later, our data confirms that no single marker region is sufficient to distinguish the two genera, *Cyllamyces* and *Caecomyces*. Only when all the ribosomal genetic regions were combined was a separation observed (Fig. 5, Suppl. material 1: supplementary data 11a: Phylogenetic tree concatenated FUN), even showing a distinction among the *Caecomyces* varieties. *Cyllamyces* and *Caecomyces* were also separated in the shm tree (see Suppl. material 1: supplementary data 11c_shm_all and Suppl. material 1: supplementary data 6d_shm_highest), though the *Caecomyces* varieties were not clearly distinguished. After analysing individual *rrn* genetic regions, it seems that these genera share much of their ribosomal operon, including IGS1, 5S, IGS2, ETS2, ITS1, 5.8S and ITS2 (see Suppl. material 1: supplementary data 11h–q). These make them too similar to consistently segregate them as two genera. The genetic regions that mark a difference between *Caecomyces* and *Cyllamyces* are ETS1 and SSU, though SSU did not differentiate between *Caecomyces* variants (Suppl. material 1: supplementary data 11q).

Moreover, on a variety level, C. communis var. communis and C. communis var. churrovis were distinguishable using the ETS1, ITS1, and LSU genetic markers. However, *Cyllamyces* appeared in a monophyletic clade with C. communis var. communis in the LSU tree and with C. communis var. churrovis in the ITS1 tree. The ETS1 phylogeny largely resolved the *Cyllamyces* and *Caecomyces* lineages, however, two of the 13 *Cyllamyces* consensus sequences clustered within the Caecomyces communis var. communis clade, while two of the four C. communis var. communis consensus sequences clustered within the *Cyllamyces* clade (Suppl. material 1: supplementary data 11f), indicating that these taxa share ETS1 consensus variants. These facts demonstrate the difficulty of basing the differentiation of these genera solely on genomic percentage differences.

The lack of consistent phylogenetic separation between *Cyllamyces* and *Caecomyces* across *rrn* genetic regions suggests that these genera may be more closely related than expected. This proximity could reflect a shared evolutionary history with a recent divergence, as the morphological trait (mono- or polycentric) is clear. Whether *Cyllamyces* and *Caecomyces* are two genera or different morphotypes of one genus remains unresolved. Nevertheless, in the *Neocallimastigomycota* phylum, it has not been observed for two morphotypes to be present within the same genus. This is more commonly seen at the family level, as in the cases of the *Neocallimastigaceae* and *Anaeromycetaceae* families, which contain genera with these two morphotypes (Suppl. material 1: supplementary data 7). Improving reference data, including more isolates (especially for *Cyllamyces*), and concatenating additional coding genes may further clarify this clade separation.

### Identification of new barcodes in the *Neocallimastigomycota rrn* operon

Mapping of previously developed primers across the *rrn* operon clearly shows overlapping sequencing efforts, particularly targeting ITS1 and LSU (e.g. Edwards et al. 2017), while overlooking other potentially informative genetic regions within the operon (see Suppl. material 1: supplementary data 12, *rrn* primers map, and Suppl. material 1: supplementary data 13, list of mapped primers).

**Figure 6.**
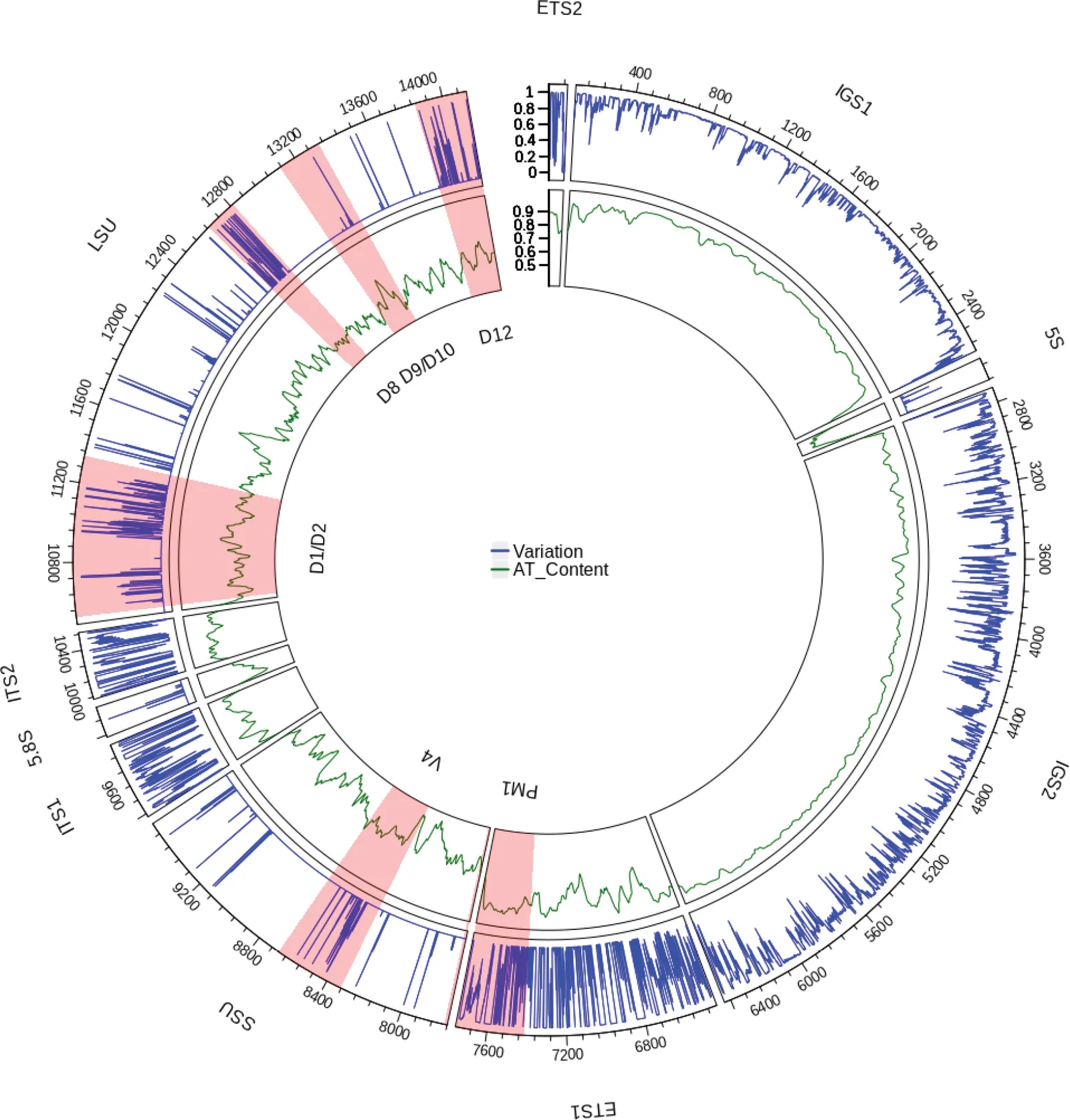
This Circos plot shows the variation and AT content of the rrn. The blue outer graph depicts sequence variation, and the green inner graph illustrates the AT content per region of the entire ribosomal operon (FUN and concatenated FUN), as defined by a running average every 20 base pairs. The interval between the measurement lines is 80 base pairs. The red-marked segments represent the evaluated regions. PM: potential marker. n = 143 consensus sequences.

Closer examination of the other less studied regions of the ribosomal operon in AGF allowed us to identify both conserved and variable regions, as well as easily alignable segments. We identified regions within the *rrn* with a comparable level of sequence divergence to that observed in the D1/D2 of the LSU in AGF because that region has already been established as a useful marker (Hanafy et al. 2020a; Hanafy et al. 2023). Additionally, the chosen regions should contain segments with high variability between conserved parts to allow for phylogenetic resolution at different taxonomic levels. Our analysis led to the identification of five regions: Potential Marker 1 (PM1), located at the end part of the ETS1; V4 in the SSU; and the LSU regions D8, D9/D10, and D12 (Fig. 6). The V4-SSU and D8-LSU regions have previously been used as phylogenetic markers for the sister fungal lineage *Chytridiomycota* before (Kilias et al. 2020 and Wurzbacher et al. 2014, respectively). In our study, these regions resolved relationships between various AGF families and genera but failed to do so at the species level or below. V4 resolved the *Cyllamyces* and *Caecomyces* clades, but it could not segregate the *Caecomyces* varieties. This was also observed within the species clades of the *Piromyces* genus (see Suppl. material 1: supplementary data 11s). D8 and D9/D10 exhibited poor resolution of multiple genera (see Suppl. material 1: supplementary data 11t, u). D12 analysis showed better genus-level resolution than the other LSU regions analyzed, but the *Neocallimastigomycota* topology was not properly represented (see Suppl. material 1: supplementary data 11v). The only informative region of these potential markers was situated in the PM1 region, which was mainly located at the end of the ETS1 (the last 230 base pairs) and at the beginning of the SSU. The variability values of the PM1 region were closest to those of D1/D2. This suggests that the high phylogenetic resolution of the ETS1 region may be due to the PM1 region.

D1/D2 showed low variability at each taxonomic level analysed: 7.22% (SD 3.46) within the phylum, 1.13% (SD 0.94) within genera, 0.34% (SD 0.25) within species, and 0.10% (SD 0.06) between strains. This variability is relatively low, yet it still provides stable phylogenetic resolution. PM1 showed higher variability within the phylum (27.08%, SD 10.75) and between genera (5.46%, SD 4.38), species (2.56%, SD 2.58), and strains (1.25%, SD 0.95). For a deeper evaluation of PM1, a phylogenetic tree was generated, showing a reliable *Cyllamyces*/*Caecomyces* clade separation, as well as for the *Caecomyces* varieties, and the genus *Piromyces* was divided into the seven species analysed. However, paraphyletic clades were observed for *Pecoramyces* and *Neocallimastix* (Suppl. material 1: supplementary data 11g). Therefore, the PM1 marker could be used only to differentiate *Cyllamyces* from *Caecomyces* and its varieties, and *Piromyces* species from one another. The short length of this amplicon (approx. 230 bp) makes it suitable for analysis with other technologies, such as Illumina sequencing and quantification by qPCR. Further primer design and optimization are required to enable using this genetic marker, PM1, as part of a multiple-marker approach for phylogenetic analysis and for screening environmental samples.

### Family-level resolution of the *Neocallimastigomycota rrn* operon

In 2023, Hanafy et al. proposed the creation of four families within the *Neocallimastigomycota* phylum: the *Anaeromycetaceae*, *Caecomycetaceae*, *Piromycetaceae*, and *Neocallimastigaceae* families. The phylogenetic tree generated with the ribosomal region without IGS1 (Fig. 5, Suppl. material 1: supplementary data 11a), was consistent with the topology of the four families: *Anaeromycetaceae*, a family that includes the genera *Anaeromyces*, *Capellomyces*, *Liebetanzomyces*, and *Oontomyces*, the *Caecomycetaceae*, which comprises *Caecomyces* and *Cyllamyces*, *Neocallimastigaceae* consisting of *Aestipascuomyces*, *Feramyces*, *Ghazallomyces*, *Neocallimastix*, *Orpinomyces* and *Pecoramyces*, as well as the *Piromycetaceae*, which comprises only *Piromyces*. The clades containing *Agriosomyces*, *Aklioshbomyces*, *Buwchfawromyces* and *Joblinomyces* (all assigned *incertae sedis* in Hanafy et al. 2023), as for *Paucimyces*, *Khoyollomyces*, and the new NC3 and YL2 clades, resulted in alternative topologies when comparing the phylogenetic trees generated with the multiple genetic markers. The position of *Tahromyces* remains unclear because only two rc consensus sequences could be generated from strain BRP051 (Suppl. material 1: supplementary data 11e). These were most closely related to *Buwchfawromyces eastonii* and *Joblinomyces apicalis*; however, *Tahromyces* could not be clearly assigned to either family. Thus, more isolates need to be examined to obtain a clearer picture. Further analysis with a larger dataset with more isolates and concatenated orthologous housekeeping/non-ribosomal genes, such as rpb1, may justify defining new family-level clades.

## Conclusions

In this study, we assembled the most comprehensive collection of the ribosomal operon sequences from anaerobic gut fungi to date. This collection is a significant milestone because it thoroughly maps the metadata of 156 anaerobic gut fungal isolates. This robust inventory facilitates referencing existing *Neocallimastigomycota* isolates and aids in classifying novel AGFs. Using high-throughput sequencing, we created the first full-length ribosomal operon database for all described *Neocallimastigomycota* up to 2022. This database enables the generation of a reference backbone for phylogenetic analysis.

We offer insights into how analyzing the *rrn* region can refine the taxonomy of the *Neocallimastigomycota* and clarify sequence variability in individual ribosomal regions. While commonly used single genetic regions partly lead to divergent tree topologies, this study achieved phylogenetic resolution by incorporating genetic information provided from each genetic region into the entire ribosomal operon of *Neocallimastigomycota*. Analyzing the entire ribosomal operon provided insight into less-studied regions, such as the ETS1 and its subregion, PM1. These regions can be used as alternative markers in multi-marker studies to differentiate the *Cyllamyces* and *Caecomyces* clades, as well as separate species within the *Piromyces* genus. The high sequence variability of the IGS reveals its potential for differentiation below species level, however, the difficulty of aligning the IGS hampers its use.

The availability of this *rrn* database allows us to apply longer sequences as markers and cross-reference the (meta) information of shorter sequences, which are often used to study AGF. Combining all informative ribosomal regions, possibly with orthologous genes, and integrating more isolates may further help annotate closely related species and variants. The resulting database, which contains consensus sequences of the entire ribosomal operon, will hopefully accelerate the exploration of anaerobic gut fungal genomics and phylogeny in *Neocallimastigomycota*, answering questions about phylogeny, diversity, taxonomy, and evolution.

## Additional information

### Artificial Intelligence (AI) use

The authors accept full responsibility for the content of the manuscript, including the disclosure of any use of AI.

No AI tools were used in the preparation of this manuscript.

### Adherence to national and international regulations

All the fungal strains used in this study have been legally obtained, respecting the Convention on Biological Diversity (Rio Convention).

### Author ORCIDs

D. Young https://orcid.org/0000-0001-6880-955X

K. Stüer-Patowsky https://orcid.org/0009-0001-1470-3095

L. Huang https://orcid.org/0000-0003-4729-837X

M.S. Elshahed https://orcid.org/0000-0002-1067-1647

N.H. Youssef https://orcid.org/0000-0001-9159-3785

R. Hanafy https://orcid.org/0000-0002-2712-9942

Y. Cheng https://orcid.org/0000-0001-8935-9551

C.D. Moon https://orcid.org/0000-0001-9692-9559

M. Stabel https://orcid.org/0000-0002-9694-2590

K. Ochsenreither https://orcid.org/0000-0002-5797-2789

S.S. Dagar https://orcid.org/0000-0003-2640-0793

E. Hillman https://orcid.org/0000-0002-3846-1692

K.V. Solomon https://orcid.org/0000-0003-2904-9118

K.O. Fliegerová https://orcid.org/0000-0001-9439-8168

G.W. Griffith https://orcid.org/0000-0001-6914-3745

S.M. Podmirseg https://orcid.org/0000-0003-1775-2412

A. Sczyrba https://orcid.org/0000-0002-4405-3847

V. Flad https://orcid.org/0000-0003-3371-2023

M. Lebuhn https://orcid.org/0000-0001-9889-8688

C. Wurzbacher https://orcid.org/0000-0001-7418-0831

### Data availability

The consensus sequences generated and analysed during the current study are available at NCBI under the Accession Numbers for FUN full-length sequenced with MinION: PZ357937–PZ358123 (BankIt3078311, n = 187), for RC sequenced with MinION: PZ358188–PZ358439 (BankIt3078746, n = 252), for RC sequenced with PacBio: PZ358440–PZ358523 (BankIt3078958, n = 84), for shm sequenced with MinION: PX908483–PX908697 (GenBank SUB15739953, n = 215), and for shm sequenced with PacBio: PX897217–PX897271 (GenBank SUB15739988, n = 55). The whole ribosomal operon alignment including the concatenated sequences (shm & RC) and uniformly oriented 5S regions is found in the supplementary data 8-Full Alignment and available on https://anaerobicfungi.org. The underlying code (pipeline and scripts) to reproduce the raw data analysis for this study is available in Github and can be accessed via github.com/rhinempi/longread_umi-AF-LSU.

## Supplementary materials

Supplementary material 1 Supplementary Information Data type zip Explanation note **supplementary data 1**. Isolates Table Batches 1-5. **supplementary data 2**. Index Primers. **supplementary data 3**. Peak detection. **supplementary data 4**. Pipeline sorting. **supplementary data 5a**. Pipeline evaluation. **supplementary data 5b**. Variants evaluation. **supplementary data 6**. Ribosomal operon variants per isolate. **supplementary data 7**. Summary AGF morphology and rrn. **supplementary data 8**. CVSD per genetic marker, multiple alignment, Newick trees. **supplementary data 9**. Alignment sketch. **supplementary data 10**. 5S discussion. **supplementary data 11**. a to v: Phylogenetic trees. **supplementary data 12**. rrn primers map. **supplementary data 13**. List of mapped primers and references. **supplementary data 14**. Sensitivity analysis of main phylogenetic tree (Fig. 5). https://binary.pensoft.net/file/1754537This dataset is made available under the Open Database License (http://opendatacommons.org/licenses/odbl/1.0/). The Open Database License (ODbL) is a license agreement intended to allow users to freely share, modify, and use this Dataset while maintaining this same freedom for others, provided that the original source and author(s) are credited. Diana Young, Katrin Stüer-Patowsky, Liren Huang, Mostafa S. Elshahed, Noha H. Youssef, Radwa Hanafy, Yanfen Cheng, Christina D. Moon, Priya Soni, Akshay Joshi, Marcus Stabel, Katrin Ochsenreither, Sumit Singh Dagar, Ethan Hillman, Kevin V. Solomon, Katerina O. Fliegerová, Gareth W. Griffith, Tony M. Callaghan, Sabine M. Podmirseg, Alexander Sczyrba, Veronika Flad, Michael Lebuhn, Christian Wurzbacher
