# Supplementary material for: Full ribosomal operon sequencing of anaerobic gut fungi (phylum Neocallimastigomycota): insights on its markers and phylogenetic resolution
Source: IMA Fungus. 2026 Aug 26;17:e195921. doi: 10.3897/imafungus.17.195921 (PMC13539303; doi:10.3897/imafungus.17.195921)
Supplement: Supplementary file 1 [file imafungus-17-e195921-s001.zip › Supplementary_Data_1 to 14/Supplementary Data 13_List of mapped primers and references.pdf]

Primer name	Sequence 5´- 3´	Reference	Marker for
ETS/SSU F	TTAATTAATAGTAAATAAAGAT	Defined in this study	PM1
ETS/SSU R	AGTTACCTGGTTGATCCTGCCA	Defined in this study	PM1
NL1_F_D1/D2	GCATATCAATAAGCGGAGGAAAAG	Fliegerova_2006	D1/D2
NL4 R D1/D2	CCGTCTTGAAACACGGACC	Fliegerova_2006	D1/D2
GA20 F D8	GTAACTTCGGGAWAAGGATTGGCT	Machida_2012	D8
RM5 R D8	GGGAATCYRACTGTHTAATTAAA	Machida_2012	D8
FD8	GGATTGGCTCTGAGGGTTGGG	Chinain_1999	
RB	GATAGGAAGAGCCGACATCGA	Chinain_1999	
GLD8_421F	ACAGCCAAGGGAACGGGCTT	Nishimura_2013	
GLD8_677R	TGTGCCGCCCCAGCCAAACT	Nishimura_2013	
D9 F	AAGACCCTGTTGAGYTTGACTC	Machida_2012	D9/D10
D10 R	GGGAGTTTGGCTGGGGCGG	Machida_2012	D9/D10
515F V4	GTGCCAGCMGCCGCGGTAA	Caporaso 2011	
806R V4	GGACTACHVGGGTWTCTAAT	Caporaso 2011	
515F-Y V4/V5	GTGYCAGCMGCCGCGGTAA	Parada 2016	V4
926R V4/V5	CCGYCAATTYMTTTRAGTTT	Parada 2016	V4
EnvS F D2	GCGTTTRRCACCASTGTTGTT	Young et al., 2022	
EnvS R D2	GTCAACATCCTAAGYGTAGGTA	Young et al., 2022	
309e_18S	TCAGGCTCCCTCTCCGG	Dore and Stahl 1991	
519_18S	GWATTACCGCGGCKGCTG	Dore and Stahl 1991	
686e_18S	AGAATTTCACCTCTG	Dore and Stahl 1991	
926e_18S	CCGTCAATTC(AC)TTT(AG)AGTTT	Dore and Stahl 1991	
18J.CPM_18S	CAGACACTACGGGAATCT	Dore and Stahl 1991	
1400_18S	ACGGGCGGTGTGT(GA)C	Dore and Stahl 1991	
915_18S	GCCCCCG(TC)CAATTCCT	Dore and Stahl 1991	
920_18S	ATTCCTTT(GA)AGTTT	Dore and Stahl 1991	
956_18S	GGCGTTGTGTC(CG)AATTAA	Dore and Stahl 1991	
926e_18S_no brackets	CCGTCAATTC	Dore and Stahl 1991	
18J.CPM_18S_no brackets	CAGACACTACGGGAATCT	Dore and Stahl 1991	
1400_18S_no brackets	ACGGGCGGTGTGT	Dore and Stahl 1991	
915_18S_no brackets	CAATTCCT	Dore and Stahl 1991	
920_18S_no brackets	ATTCCTTT	Dore and Stahl 1991	
956_18S_no brackets	GGCGTTGTGTC	Dore and Stahl 1991	
1100_18S	AGGGTTGCGCTCGTT	Dore and Stahl 1991	
1100a_18S	TGGGTCTCGCTCGTTG	Dore and Stahl 1991	
1511e_18S	C(CT)GCAGGTTCACCTAC	Dore and Stahl 1991	
1511e_18S_no brackets	GCAGGTTCACCTAC	Dore and Stahl 1991	
SL2l (R)_18S	CCGAATTCGTAGTCATATGCTTGTCT	Bowman et al., 1992	
SL27 (F)_18S	CCAAGCTTAAACCTTGTTACGACTT	Bowman et al., 1992	
NS1 (F)_18S	GTAGTCATATGCTTGTCTC	Fliegerova et al., 2006	
NS2 (R)_18S	GGCTGCTGGCACCAGACTTGC	Fliegerova et al., 2006	
AF-SSU forward_18S	CTAGGGATCGGACGACGTTT	Dollhofer et al., 2016	
AF-SSU reverse_18S	GGACCTYCCGATCAAGGATG	Dollhofer et al., 2016	
AF-SSU probe_18S	ATTCGCGTAACTATTTAGCAGGTTAAGGT	Dollhofer et al., 2016	
(F)_ITS1	TGTACACACCGCCCGTC	Li and Heath, 1992, Brookman et al., 2000, Hausner et al., 2000	
(R)_ITS1	CTGCGTTCTTCATCGAT	Li and Heath, 1992, Brookman et al., 2000, Hausner et al., 2000	
ITS 1 (F)_ITS1	TCCGTAGGTGAACCTGCGG	Fliegerova et al., 2002	
ITS 2 (R)_ITS1	GCTGCGTTCTTCATCGATGC	Fliegerova et al., 2002	
MN100 (F)_ITS1	TCCTACCCTTTGTGAATTTG	Tuckwell et al., 2005	
MNGM2 (R)_ITS1	CTGCGTTCTTCATCGTTGCG	Tuckwell et al., 2005	
Neo 18S (F)	6FAM-AATCCTTCGGATTGGCT	Edwards et al., 2008	
Neo 5.8S (R)	CGAGAACCAAGAGATCCA	Edwards et al., 2008	
(F)_ITS1before	GCCTCCCTCGCGCCATCAG	before barcode Liggenstoffer et al., 2010	
(F)_ITS1after	TCCTACCCTTTGTGAATTTG	after barcode Liggenstoffer et al., 2010	
(R)_ITS1	GCCTTGCCAGCCCGCTCAGCTGCGTTCTTCATCGTTGCG	Liggenstoffer et al., 2010	
ITS1F_ITS1	CTTGGTCATTTAGAGGAAGTAA	Fliegerova et al., 2010	
Neo QPCR (R)_ITS1	GTGCAATATGCGTTCGAAGATT	Fliegerova et al., 2010	
qRT-PCR-fungi (F)_ITS1	GAGGAAGTAAAAGTCGTAACAAGGTTTC	Lwin et al., 2011	
qRT-PCR-fungi (R)_ITS1	CAAATTCACAAAGGGTAGGATGATT	Lwin et al., 2011	
ITS1F (F)	TCCGTAGGTGAACCTGCGG	Kittelmann et al., 2012	
ITS400Rw (R)	ATTGTCAAAAGTTGTTTTTAWATTAT	Kittelmann et al., 2012	
Neo QPCR (F)_5.8S	TTGACAATGGATCTCTTGGTTCTC	Edwards et al., 2008	
Neo QPCR (R)_5.8S	GTGCAATATGCGTTCGAAGATT	Edwards et al., 2008	
(F)_SSU and ITS1	GAGGAAGTAAAAGTCGTAACAAGGTTTC	Denman and Mcsweeney, 2006	
(R)_SSU and ITS1	CAAATTCACAAAGGGTAGGATGATT	Denman and Mcsweeney, 2006	
ITS 1 (F)	TCCGTAGGTGAACCTGCGG	Fliegerova et al., 2002, 2006	
ITS 4 (R)	TCCTCCGCTTATTGATATGC	Fliegerova et al., 2002, 2006	
JB206 (F)_ITS	GGAAGTAAAAGTCGTAACAAGG	Tuckwell et al., 2005	
JB205 (R)_ITS	TCCTCCGCTTATTAATATGC	Tuckwell et al., 2005	
GM1 (F)_ITS	TGTACACACCGCCCGTC	Nicholson et al., 2010	
JB205 (R)_ITS	TCCTCCGCTTATTAATATGC	Nicholson et al., 2010	
ITS1F (F)	CTTGGTCATTTAGAGGAAGTAA	Griffith et al., 2009	
EminITS4 (R)	GTTCAGCGGGTACTCTTATCTG	Griffith et al., 2009	
JB206 (F)_ITS	GGAAGTAAAAGTCGTAACAAGG	Solomon et al., 2016a	
JB205 (R)_ITS	TCCTCCGCTTATTAATATGC	Solomon et al., 2016a	
(F)_LSU	GCCTTAGTAACGGCGAGTG	Hausner et al., 2000	
(R)_LSU	GGAACCTTTCCCCACTTC	Hausner et al., 2000	
AF-LSU (F)	GCTCAAAYTTGAAATCTTMAAG	Dollhofer et al., 2016	
AF-LSU (R)	CTTGTTAAMYRAAAAGTGCATT	Dollhofer et al., 2016	
ITS5 (F)	GGAAGTAAAAGTCGTAACAAGG	Wang et al., 2017	
(F)_IGS	GAGACAAGCATATGACTAC	Hausner et al., 2000	
(R)_IGS	ACGCCTCTAAGTCAGAAT	Hausner et al., 2000	
AF-Endo (F)_GH5	CGTATTCCAACYACTTGGWSYGG	Dollhofer et al., 2016	
AF-Endo (R)_GH5	CCRKTRTTTAAGGCAAARTTRTAYGGA	Dollhofer et al., 2016	
NS1sh_rRNA shm	CAGTAGTCATATGCTTGTC	Wurzbacher et al., 2019	shm
RCA95m_rRNA shm	CTATGTTTTAATTAGACAGTCAG	Wurzbacher et al., 2019	shm
F-rOP-F_FUN	CTGACTGTCTAATTAAAACAT	Wurzbacher et al., 2019	FUN
F-rOP-R_FUN	TCAGATTCCCCTTGTCCGTA	Wurzbacher et al., 2019	FUN
NS1rc_IGS RC	GACAAGCATATGACTACTG	Wurzbacher et al., 2019	RC
RCA95rc_IGS RC	CTGACTGTCTAATTAAAACATAG	Wurzbacher et al., 2019	RC

Gene or region	Organism	NCBI Reference Sequence	Marker for
5S ribosomal RNA (RDN5-6)	Saccharomyces cerevisiae S288C	NR_132228.1	5S
18S ribosomal RNA (RDN18-2)	Saccharomyces cerevisiae S288C	NR_132222.1	SSU
5.8S ribosomal RNA (RDN58-1)	Saccharomyces cerevisiae S288C	NR_132211.1	5.8
25S ribosomal RNA (RDN25-2)	Saccharomyces cerevisiae S288C	NR_132218.1	LSU
