# Supplementary material for: Full ribosomal operon sequencing of anaerobic gut fungi (phylum Neocallimastigomycota): insights on its markers and phylogenetic resolution
Source: IMA Fungus. 2026 Aug 26;17:e195921. doi: 10.3897/imafungus.17.195921 (PMC13539303; doi:10.3897/imafungus.17.195921)
Supplement: Supplementary file 1 [file imafungus-17-e195921-s001.zip › Supplementary_Data_1 to 14/Supplementary Data 4_Pipeline sorting.pdf]

Supplementary Data 4: Pipeline sorting

Number of sequences resulting from each Pipeline step until consensus sequences were obtained. *PacBio sequences were already demultiplexed.

	Pipeline step 1	Pipeline step 2	Pipeline step 3	Pipeline step 4	Pipeline step 5	Pipeline step 6	Pipeline step 7	Total reliable consensus sequences
Batch	Input / base calling	Filtered/ Demultiplexed	Filtered input	Denoised / clustering	Consensus Sequences	Manual polish (deleted consensus sequences)	Chimeras (deleted consensus sequences)	
**Batch 1**	1,326,343	376,283	28%	312,643	383	45	2	336
**Batch 2**	1,524,641	412,117	27%	349,779	321	51	-	270
**Batch 3**	2,495,914	2,495,914	100% *	1,487,140	153	10	1	142
**Batch 4**	6,051	306	5%	181	8	2	-	6
**Batch 5**	75,041	73,626	98%	39,285	28	12	-	16
**Total**	**5,427,990**	**3,358,246**		**2,189,028**	**893**	**120**	**3**	**770**
