# Supplementary material for: Full ribosomal operon sequencing of anaerobic gut fungi (phylum Neocallimastigomycota): insights on its markers and phylogenetic resolution
Source: IMA Fungus. 2026 Aug 26;17:e195921. doi: 10.3897/imafungus.17.195921 (PMC13539303; doi:10.3897/imafungus.17.195921)
Supplement: Supplementary file 1 [file imafungus-17-e195921-s001.zip › Supplementary_Data_1 to 14/Supplementary Data 2_Index Primers.pdf]

Supplementary Data 2. Index Primers

	Barcode	Adapter	Spacer	RC, sh-m, or FUN	Primer name	Amplicon name
1st PCR		**TCGTCGGCAGCGTC**	AA	GACAAGCATATGACTACTG	NS1rc-Ad	IGS (rc)
		**GTCTCGTGGGCTCGG**	AA	CTGACTGTCTAATTAAAACATAG	RCA95rc-Ad	
		**TCGTCGGCAGCGTC**	TT	CAGTAGTCATATGCTTGTC	NS1sh-Ad	rRNA (shm)
		**GTCTCGTGGGCTCGG**	AA	CTATGTTTTAATTAGACAGTCAG	RCA95m-Ad	
		**TCGTCGGCAGCGTC**	TA	CTGACTGTCTAATTAAAACAT	F-rOP-F-Ad	Complete operon tandem repeat (FUN)
		**GTCTCGTGGGCTCGG**	TT	TCAGATTCCCCTTGTCCGTA	F-rOP-R-Ad	

2nd PCR	rev-bc-1	ATGCTTGTAGGCA	**GTCTCGTGGGCTCGG**
	rev-bc-2	TAATTGTGTCGGA	**GTCTCGTGGGCTCGG**
	rev-bc-3	AAGCGGAGGTTAG	**GTCTCGTGGGCTCGG**
	rev-bc-4	ATGCAGTCCTCGA	**GTCTCGTGGGCTCGG**
	rev-bc-5	ATGGAGGTTCTCA	**GTCTCGTGGGCTCGG**
	rev-bc-6	ATTGTACTCACTC	**GTCTCGTGGGCTCGG**
	rev-bc-7	ATTGCGTTAGCAG	**GTCTCGTGGGCTCGG**
	rev-bc-8	AGCAACACCATCC	**GTCTCGTGGGCTCGG**
	rev-bc-9	AGCTGTACGGATT	**GTCTCGTGGGCTCGG**
	rev-bc-10	AACCATAGCTCCG	**GTCTCGTGGGCTCGG**
	rev-bc-11	AGCATATGCACTG	**GTCTCGTGGGCTCGG**
	rev-bc-12	ATCGACATCTCTT	**GTCTCGTGGGCTCGG**
	fwd-bc-A	AATCGCACAGTAA	**TCGTCGGCAGCGTC**
	fwd-bc-B	ATGGTAGTCTGAA	**TCGTCGGCAGCGTC**
	fwd-bc-C	AGTCGTGTAGCCT	**TCGTCGGCAGCGTC**
	fwd-bc-D	ACCTATGCACGGT	**TCGTCGGCAGCGTC**
	fwd-bc-E	AGCGTGGTCATTA	**TCGTCGGCAGCGTC**
	fwd-bc-F	AAGTCACATCCGC	**TCGTCGGCAGCGTC**
	fwd-bc-G	AAGCGTCTGAACT	**TCGTCGGCAGCGTC**
	fwd-bc-H	AAGATTGACCAAC	**TCGTCGGCAGCGTC**

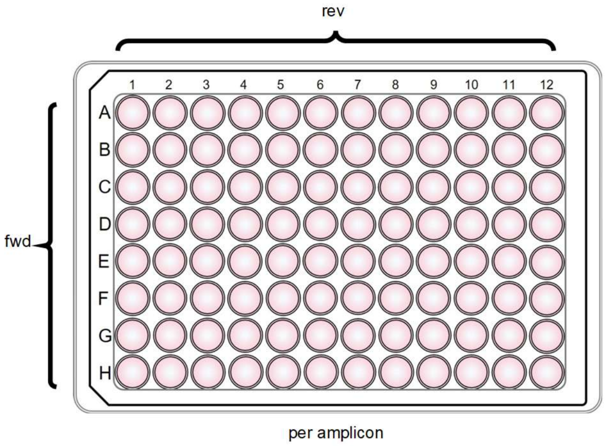

Supplementary Data 2. The primers for the first PCR are composed of an adapter, a two nucleotide spacer, and a forward or reverse primer for each of the three amplicons (rDNA: “shm”, IGS: “rc” and for the whole operon: “FUN”). The second PCR used the previously added adapter to bind the barcoding (bc) primer to each amplicon. A matrix of bc primers marked each isolate and amplicon with a unique combination distributed on a 96-well plate. All primers are direction 5´to 3´ from left to right.
