# Supplementary material for: Full ribosomal operon sequencing of anaerobic gut fungi (phylum Neocallimastigomycota): insights on its markers and phylogenetic resolution
Source: IMA Fungus. 2026 Aug 26;17:e195921. doi: 10.3897/imafungus.17.195921 (PMC13539303; doi:10.3897/imafungus.17.195921)
Supplement: Supplementary file 1 [file imafungus-17-e195921-s001.zip › Supplementary_Data_1 to 14/Supplementary_Data_14_Sensitivity_analysis.pdf]

Full ribosomal operon sequencing of anaerobic gut fungi (phylum *Neocallimastigomycota*): insights on its markers and phylogenetic resolution

Supplementary Data 14

Sensitivity analysis of phylogenetic inference with and without intergenic spacer regions

For the sensitivity analysis and phylogenetic tree of the entire ribosomal operon construction, the alignment was split into partitions (genetic regions) using the following reference positions (Table SM1).

Table SM1. Reference positions in the entire ribosomal operon alignment. The alignment can be found in the Supplementary Data 8 and is available on https://anaerobicfungi.org.

Alignment position	ETS2	IGS1	5S	IGS2	ETS1	SSU	ITS1	5.8S	ITS2	LSU
Start [position]	1	96	2,605	2,730	6,433	7,758	9,582	9,988	10,148	10,487
End [position]	95	2,604	2,729	6,432	7,757	9,581	9,987	10,147	10,486	14,123

Assuming that these regions may have differential evolutionary rates, we subsequentially filtered the alignment to remove highly variable sites using trimAl (v. 1.5.1). The gap- and similarity-filtered complete ribosomal-operon alignment comprised 149 sequences and 9,757 nucleotide positions, of which 4,131 were parsimony informative. To assess the influence of the two intergenic spacer regions, three additional alignments were generated by excluding IGS1, IGS2, or both IGS regions.

Table SM2. Effect of alignment filtering of the entire ribosomal region using trimAl with either gap filtering (-gt 0.3 -st 0.001) or an heuristic automated trimming (-automated1).

**Partition**	**Original length**	**Gap filtering retained**	**Gap filtering removed (%)**	**automated1 retained**	**automated1 removed (%)**
ETS2	95	65	31.6%	48	49.5%
IGS1	2,509	453	81.9%	80	96.8%
5S	125	125	0.0%	123	1.6%
IGS2	3,703	2,097	43.4%	280	92.4%
ETS1	1,325	938	29.2%	748	43.5%
SSU	1,824	1,807	0.9%	1,795	1.6%
ITS1	406	293	27.8%	227	44.1%
5.8S	160	159	0.6%	157	1.9%
ITS2	339	252	25.7%	116	65.8%
LSU	3,637	3,568	1.9%	3,519	3.2%
**Total**	**14,123**	**9,757**	**30.9%**	**7,093**	**49.8%**

Removal of IGS1 reduced the alignment to 9,304 positions and removed 437 parsimony-informative sites, whereas removal of IGS2 reduced it to 7,660 positions and removed 2,059 informative sites (Table SM2). The alignment lacking both spacers contained 7,207 positions and 1,635 parsimony-informative sites. The trimming of the alignment was mainly removing IGS1 sequences, and the diagnostic -automatic1 mode of trimAI that was optimized for maximum likelihood analysis confirmed that IGS1 is not informative or ambiguously in its alignment.

Overall, IGS2 was the principal source of alignment heterogeneity. Mean gap and ambiguity content similarly declined from 13.51% in the complete alignment to 11.56% after removal of IGS1, 5.59% after removal of IGS2, and 2.58% after removal of both spacers.

During the phylogenetic tree construction with IQ-TREE, the implemented ModelFinder (v. 3.1.3) identified six merged partitions for the complete alignment (Table SM3). IGS1 and IGS2 were retained as separate partitions, while ETS2, ITS1, and ITS2 were merged, as were 5S, 5.8S, and LSU. Removal of IGS1 produced the same partition structure for all remaining regions. Both spacer partitions exhibited high estimated relative evolutionary rates, whereas SSU and the merged 5S–5.8S–LSU partition evolved substantially more slowly.

Table SM3. Substitution rates of partitions using ModelFinder for the entire alignment with all regions.

**Merged partition**	**Selected model**	**Final relative rate**
ETS2 + ITS1 + ITS2	HKY+F+G4	1.592
IGS1	GTR+F+R3	5.170
5S + 5.8S + LSU	GTR+F+I+R3	0.059
IGS2	TVM+F+R4	2.717
ETS1	TVM+F+R4	0.521
SSU	TPM3u+F+I+R2	0.018

The maximum-likelihood topology was highly robust to remove IGS1. The complete and no-IGS1 trees shared 134 of 146 internal splits and had a normalized Robinson–Foulds distance of 0.082. In contrast, removal of IGS2 caused substantially greater topological change: the complete and no-IGS2 trees shared 89 internal splits and had a normalized Robinson–Foulds distance of 0.390. The tree lacking both spacer regions differed similarly from the complete tree, with a normalized distance of 0.452. The no-IGS2 and no-IGS1/no-IGS2 trees were more like each other than either was to the IGS2-containing trees, indicating that IGS2 was the primary determinant of topological differences among analyses.

Branch support declined with the progressive removal of informative sites (i.e. regions). Median ultrafast bootstrap support was 89 for the complete alignment, 88 after removal of IGS1, 83 after removal of IGS2, and 77 after removal of both spacers. Together, these analyses indicate that IGS1 has little effect on the inferred global topology, whereas IGS2 contributes substantial phylogenetic resolution but is also associated with pronounced compositional and evolutionary-rate heterogeneity. We therefore used the alignment lacking IGS1 as a conservative primary analysis and treated the remaining trees as sensitivity analyses.
