# Supplementary material for: Full ribosomal operon sequencing of anaerobic gut fungi (phylum Neocallimastigomycota): insights on its markers and phylogenetic resolution
Source: IMA Fungus. 2026 Aug 26;17:e195921. doi: 10.3897/imafungus.17.195921 (PMC13539303; doi:10.3897/imafungus.17.195921)
Supplement: Supplementary file 1 [file imafungus-17-e195921-s001.zip › Supplementary_Data_1 to 14/Supplementary Data 10_5S.pdf]

Supplementary Data 10. Further discussion about the 5S gene in the whole ribosomal operon.

The observations regarding the 5S structure were so interesting that we decided to add a supplement to our results.

A) Direction of the 5S

Another *rrn* component worth mentioning is the 5S gene, which seems to be essential for organisms to survive (Huang et al., 2020). Nevertheless, we detected a few consensus sequences with low read counts (11 to 16 reads) where no 5S sequence was found between the IGS1 and IGS2 regions. This could mean that these variants may be rare, or that some 5S genes may be located somewhere else in the respective genomes. However, sequencing artifacts cannot be fully excluded. In all other high-read sequences, 5S was found in sense or antisense direction (FUN and rc) and occurred in inverted orientations in 27.88% of all the examined consensus sequences. The inverted 5S gene was observed in seven out of the 20 genera and 3 new clades analyzed in this study, namely: *Buwchfawromyces*, *Oontomyces*, *Orpinomyces*, *Piromyces*, *Tahromyces*, and the new clades NC3 and YL2. The inversion of the 5S gene has also been detected in several eukaryotic organisms, from the tilapia fish *Oreochromis niloticus* (Martins *et al*., 2002) to fungal genera like *Saccharomyces*. Cassidy and Pukkila (1987) demonstrated that one species from the genus *Coprinopsis* (*Basidiomycota* division) presented an inverted 5S gene, different from other species in the same genus. Other fungal-like genera from the *Oomycota* division (*Pythium*, *Lagenidium*, *Pachymetra*, and *Verrucalvus*) have also been described to have an inverted 5S gene (Belkhiri *et al*., 1992). Beilhiri and Klassen (1996) detected that the presence and position of the 5S gene are related to evolutionary events and found out that the 5S gene may be linked to tandem arrays in filamentous zoosporangia fungi of the genus *Pythium* or unlinked as seen in the globose zoosporangia species of the same genus. In our observations, the morphological characteristics of the analyzed AGF isolates do not relate to the inversion of the 5S gene. The genus *Piromyces* is a monocentric, uniflagellate, and filamentous AGF and all twelve *Piromyces* isolates analyzed in our study contained an inverted 5S. These morphological characteristics are shared with other species where no 5S inversion was observed. The inversion of the 5S cannot be explained by their origin or treatment of the sample, since all the *Piromyces* strains analyzed were isolated from different hosts, maintained under different culture conditions, the DNA extraction was performed in various institutes, and further amplification and sequencing were done in independent runs (Supplementary Data 1_Isolates Table_Batches 1-5). These different observations seem to indicate that the 5S inversion works differently depending on the fungal group. In the case of the *Neocallimastigomycota*, the search for 5S inversion could help to distinguish *Piromyces* from other genera that did not present an inverted 5S, such as *Neocallimastix, Oontomyces, Pecoramyces* and *Khoyollomoyces* which also share all three mentioned morphological features but did not present inversion of the 5S gene, making it an additional attribute potentially useful for taxonomy and evolutionary studies.

We noted the special cases of one isolate (*Orpinomyces joyonii*) where 2 out of the 4 strains analyzed (L1(ME) and W212) presented the 5S gene in both, sense and antisense orientations. The more represented orientation was in the sense orientation (approx. 15,000 reads) compared to the antisense variation with not more than 200 reads in both isolates. We cannot deny the possibility of finding more 5S copies in other parts of the genome oriented in both senses for other isolates. A future screening in AGF genomes will help to reveal the importance of the 5S orientation and to determine if this feature can indeed be used as a classification tool.

B) TATA-like box for the 5S

Another possible taxonomic feature became evident by screening for the TATA-like boxes (promoters for RNA polymerase) along the 5S sequences suggesting that they might also be transcriptionally active. The motif search was optimized to WTATW, considering at least one complete TATA-motif and around 30 bases upstream or downstream of the 5S gene. Interestingly, the location of the TATA-like box in all isolates from *Capellomyces foraminis* and *Liebetanzomyces polymorphus* was upstream of the 5S gene. The isolates from *Khyollomyces ramosus*, *Ghazallomyces constrictus*, *Joblinomyces apicalis, Buwchfawromyces eastonii*, *Anaeromyces* species (*A. muctronatus* and *A. contortus*), and *Neocallimastix* (*N. cameroonii* and *N. frontalis*) presented TATA-like boxes in both flanks of the 5S gene. The genera *Orpinomyces* and *Piromyces* presented the motif in different positions (upstream or downstream in the same genus). In the case of *Orpinomyces*, even the same isolate presented one consensus sequence with TATA-like boxes downstream and another consensus sequence on both flanks. The other 13 genera presented the TATA-like boxes downstream.

These two interesting trails of the 5S raise further research questions about its transcription and importance for the ribosomal operon.

References:

Belkhiri, A., Buchko, J., and Klassen, G.R. (1992) The 5S ribosomal RNA gene in Pythium species: two different genomic locations. Mol. Biol. Evol. 9(6):1089-1102. DOI: 10.1093/oxfordjournals.molbev.a040780

Belkhiri, A., Klassen, G.R. (1996) Diverged 5s rRNA sequences adjacent to 5s rRNA genes in the rDNA of Pythium pachycaule. Curr Genet 29, 287–292. https://doi.org/10.1007/BF02221560

Cassidy, J.R. and Pukkila, P.J (1987). Inversion of 5S ribosomal RNA genes within the genus Coprinus. Curr Genet. 12(1), 33–36. doi:10.1007/bf00420724

Huang, S., Aleksashin, N.A., Loveland, A.B. et al., (2020). Ribosome engineering reveals the importance of 5S rRNA autonomy for ribosome assembly. Nat Commun 11, 2900

Martins, C.; Wasko, A.P.; Oliveira, C.; Porto-Foresti, F.; Parise-Maltempi, P.P.; Wright, J.M.; Foresti, F. (2002). Dynamics of 5S rDNA in the tilapia (Oreochromis niloticus) genome: repeat units, inverted sequences, pseudogenes and chromosome loci. Cytogenetic and Genome Research, 98(1), 78–85. doi:10.1159/000068542
