# Supplementary material for: Full ribosomal operon sequencing of anaerobic gut fungi (phylum Neocallimastigomycota): insights on its markers and phylogenetic resolution
Source: IMA Fungus. 2026 Aug 26;17:e195921. doi: 10.3897/imafungus.17.195921 (PMC13539303; doi:10.3897/imafungus.17.195921)
Supplement: Supplementary file 1 [file imafungus-17-e195921-s001.zip › Supplementary_Data_1 to 14/Supplementary Data 11_Phylogenetic trees.pdf]

Full ribosomal operon sequencing of anaerobic gut fungi (phylum *Neocallimastigomycota*): insights on its markers and phylogenetic resolution

Supplementary Data 11

Phylogenetic trees inferred with the *rrn* consensus sequences. The percentages at the nodes represent support values for the SH-like approximate likelihood ratio test (100 indicates full support for both values). The branch lengths are proportional to substitutions per site.

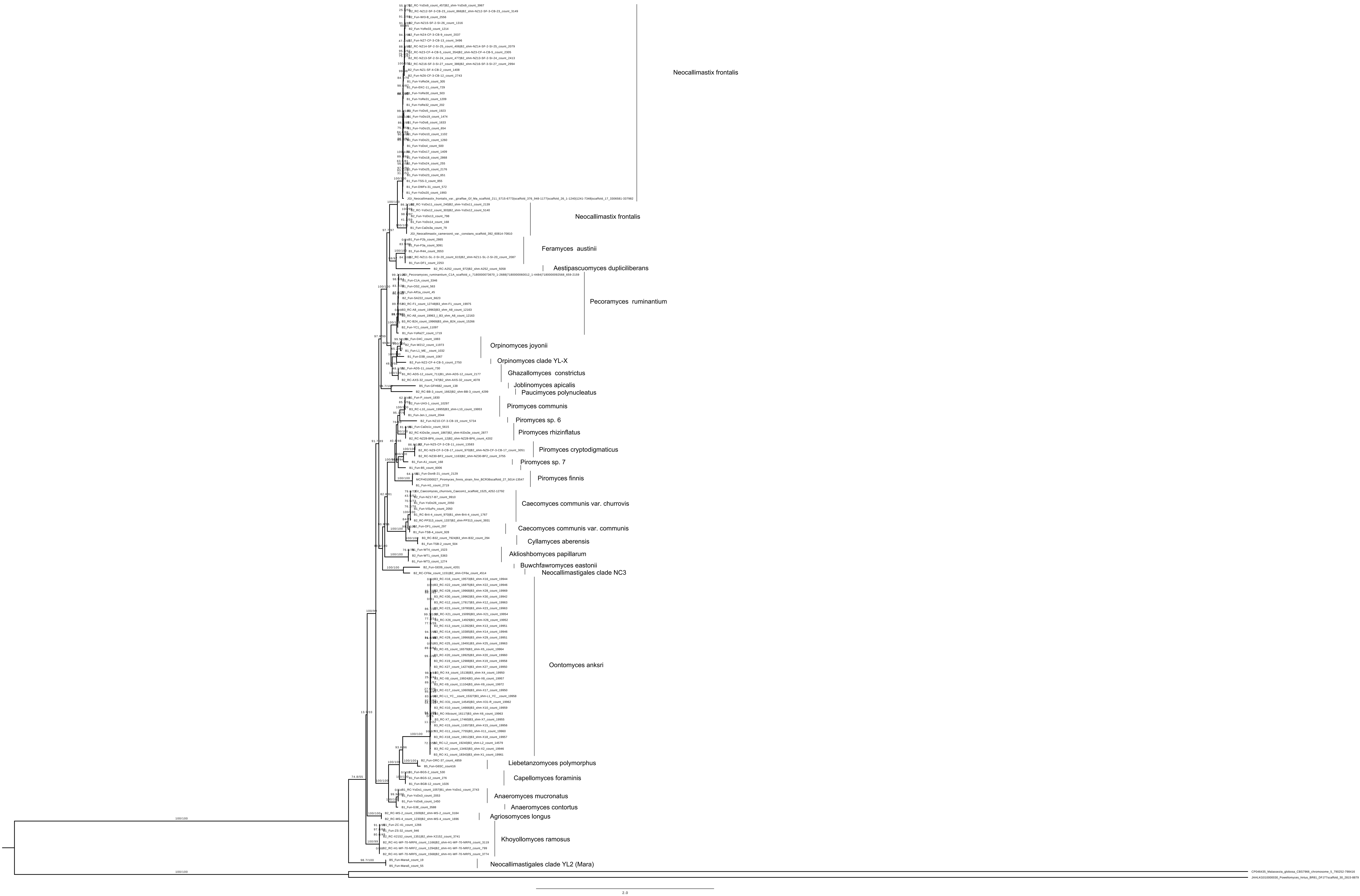

Supplementary Data 11a
concatenated FUN and all FUN
with filtered IGS1

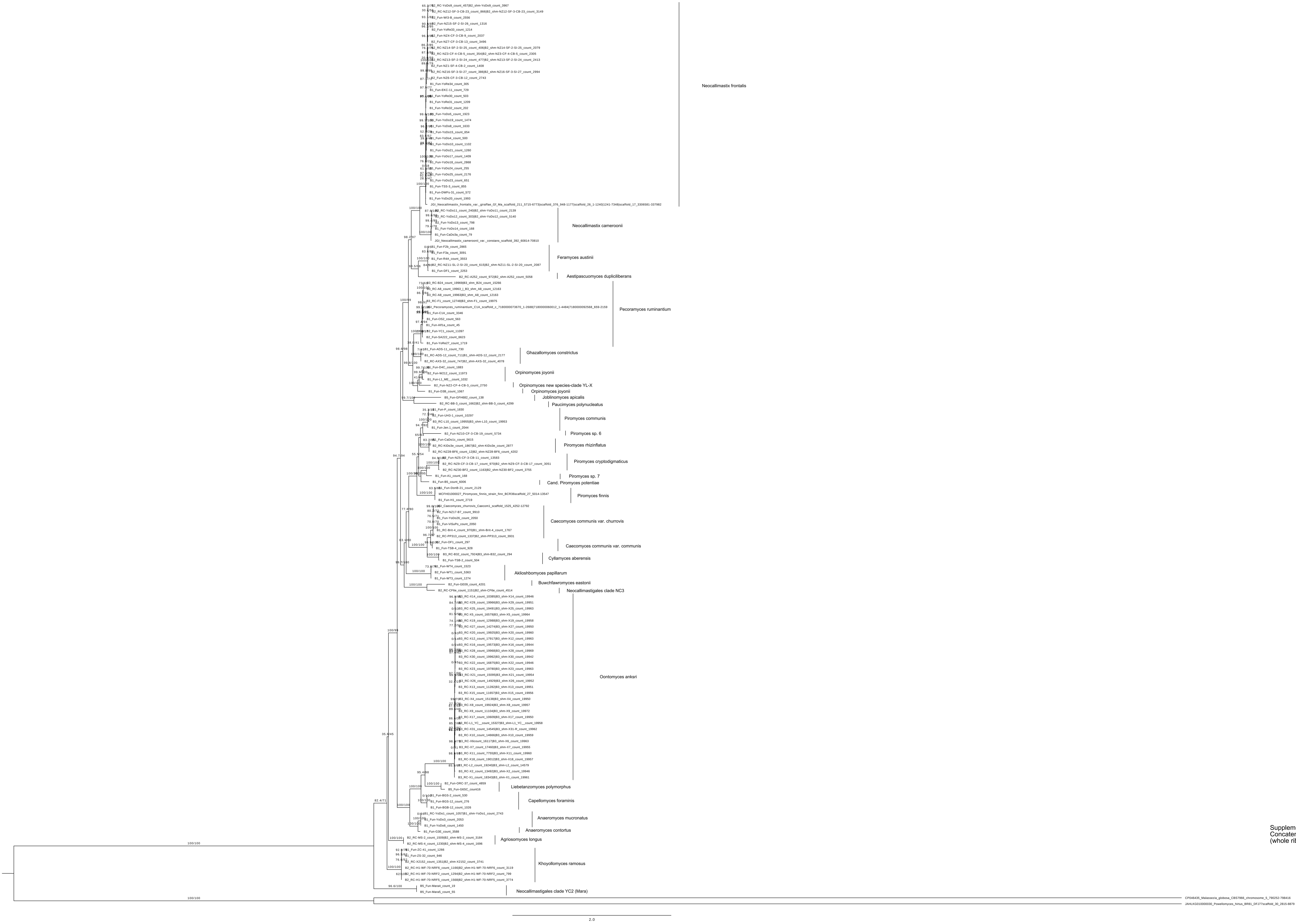

Supplementary Data 11b
Concatenated_FUN_all
(whole ribosomal operon with gap-filtering)

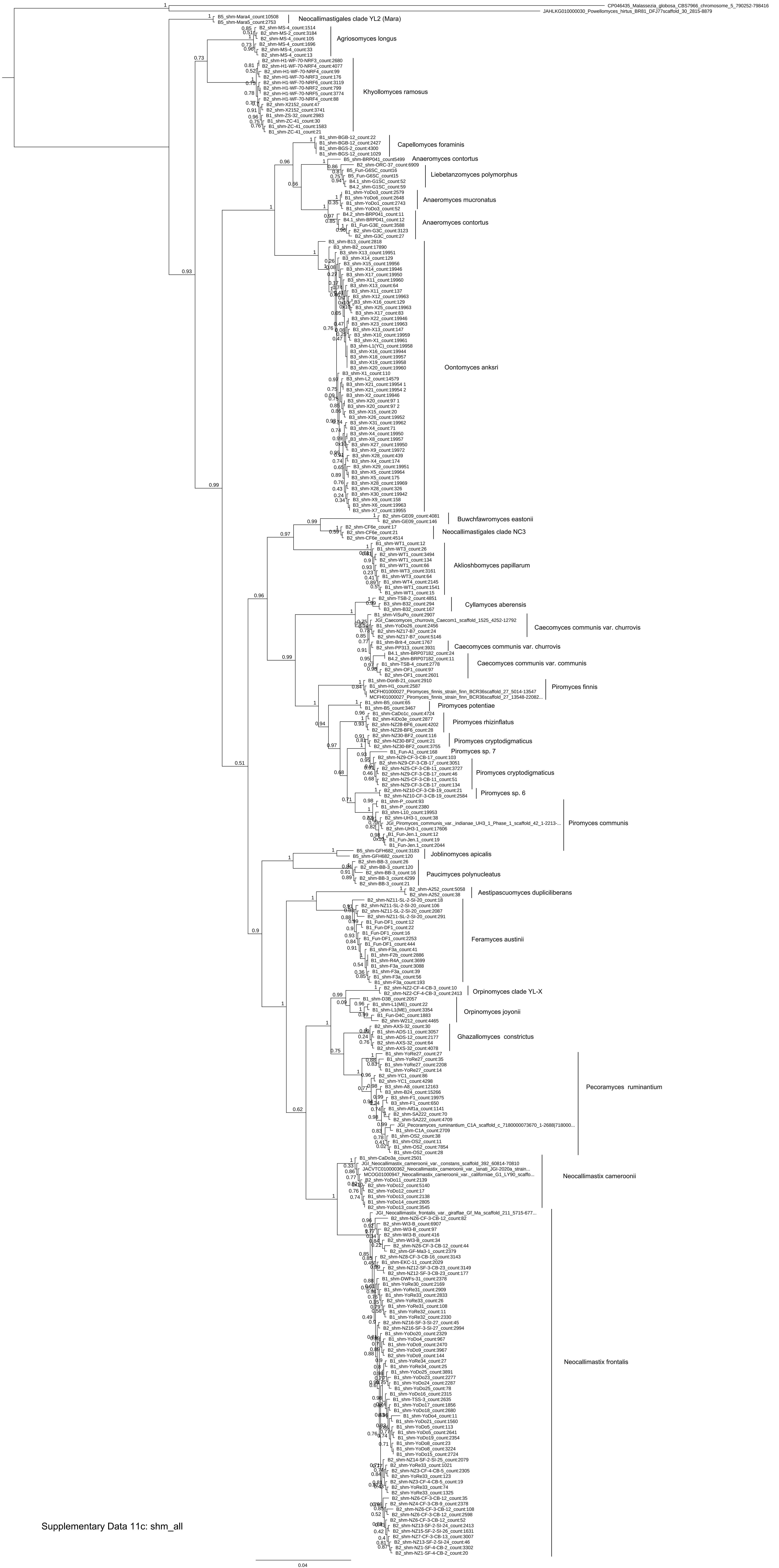

Supplementary Data 11c: shm_all

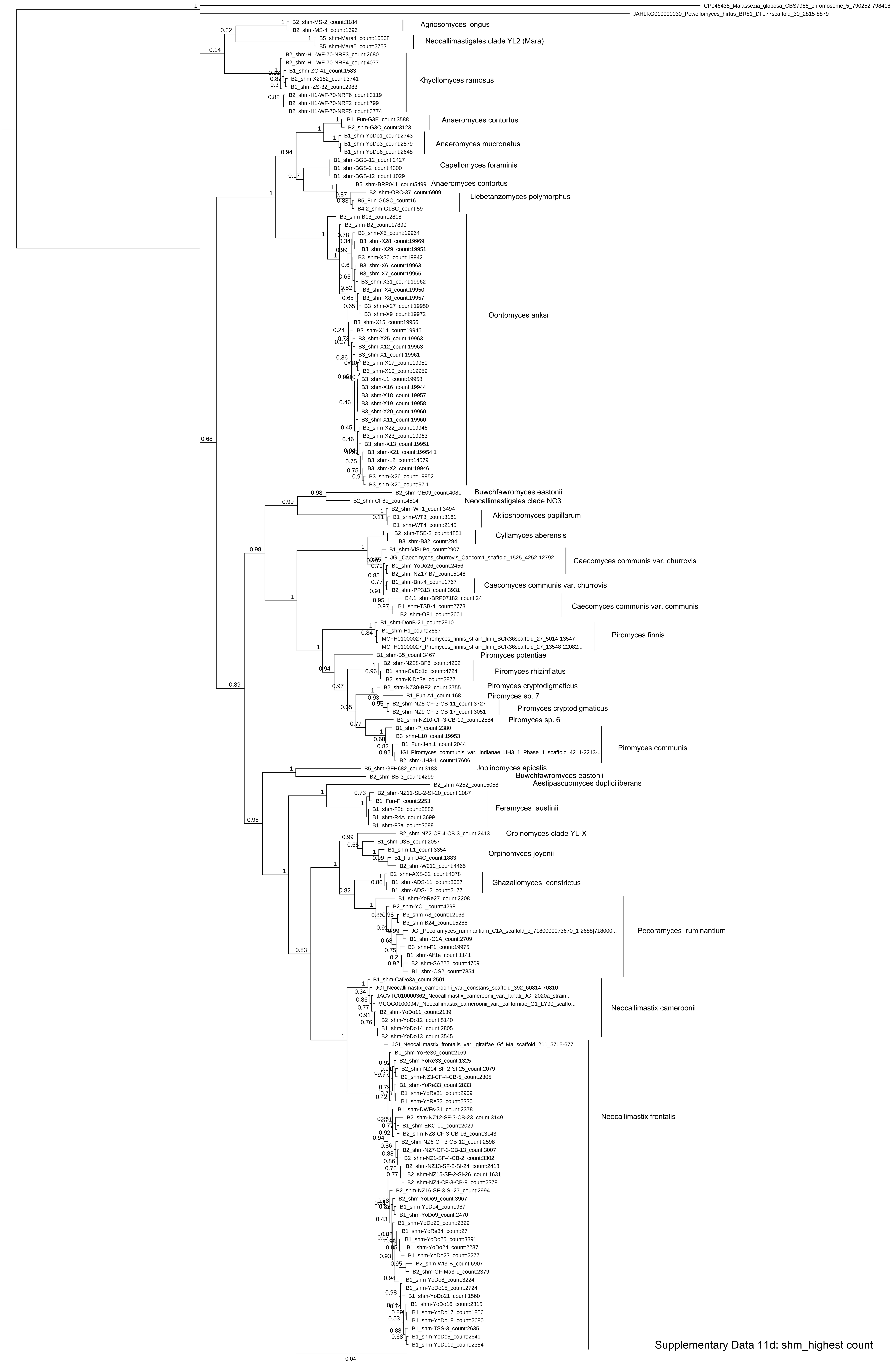

Supplementary Data 11d: shm_highest count

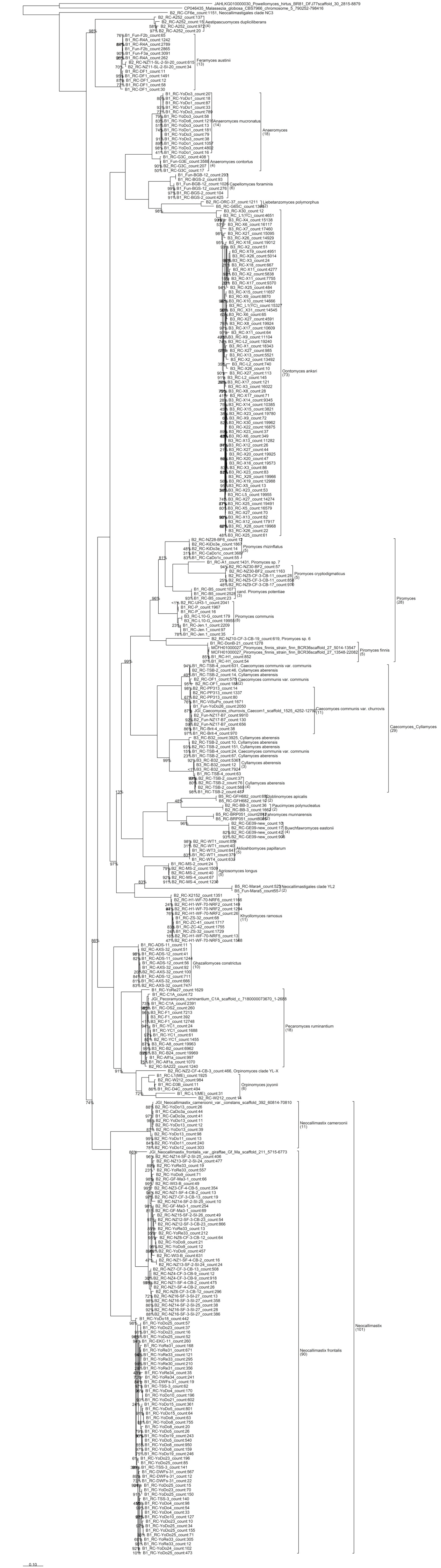

Supplementary Data 11e
RC all

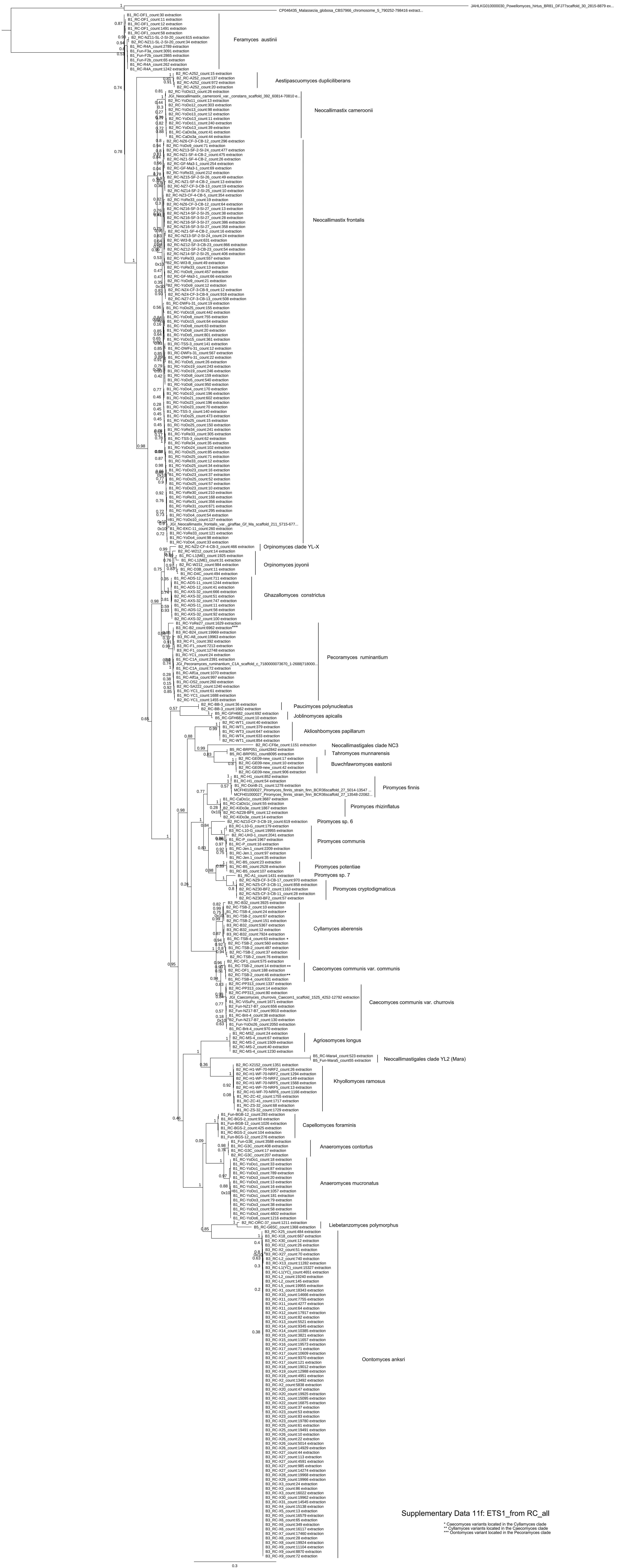

Supplementary Data 11f: ETS1_from RC_all

* Caecomyces variants located in the Cyllamyces clade
** Cyllamyces variants located in the Caecomyces clade
*** Oontomyces variant located in the Pecoramyces clade

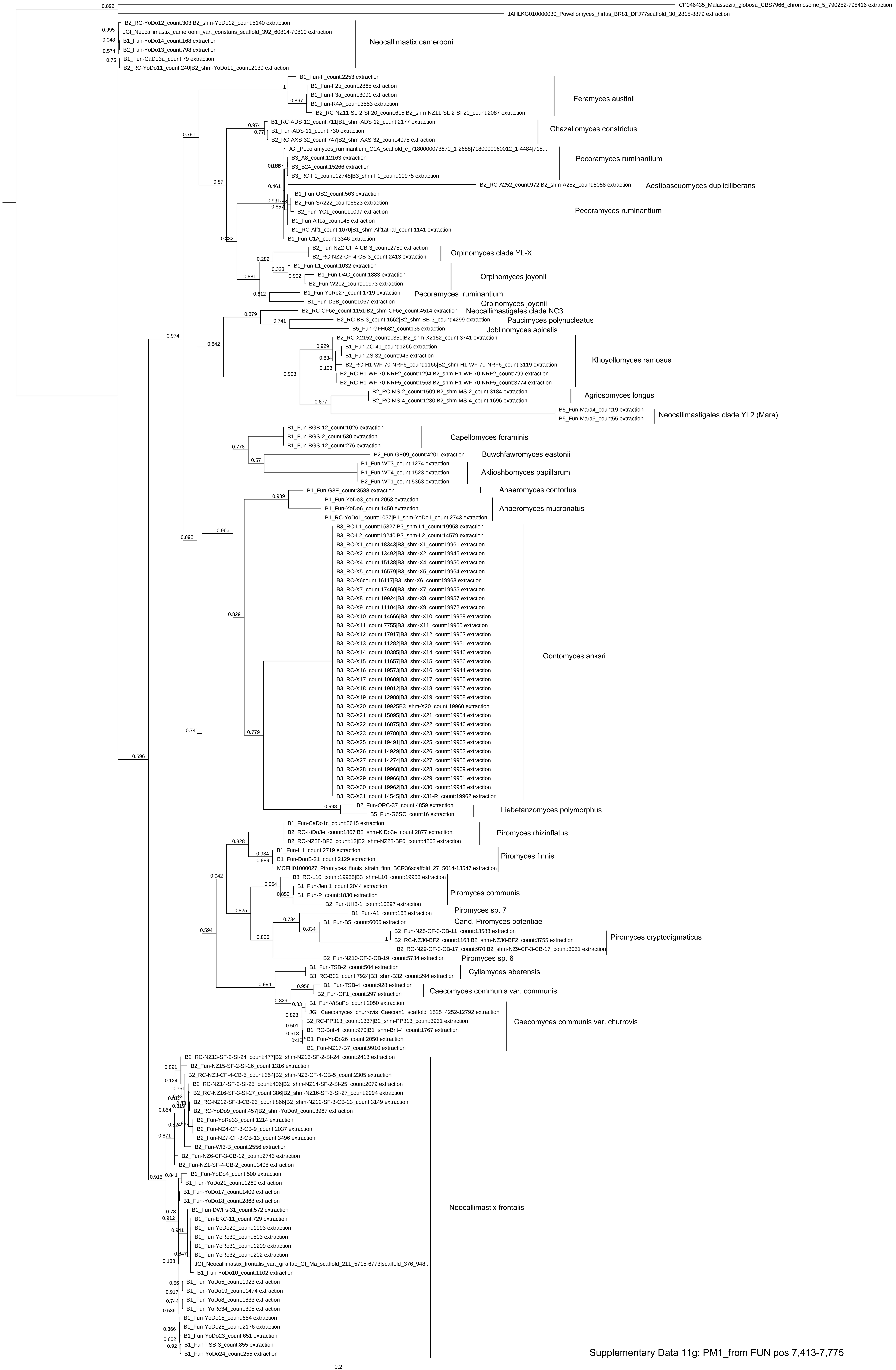

Supplementary Data 11g: PM1_from FUN pos 7,413-7,775

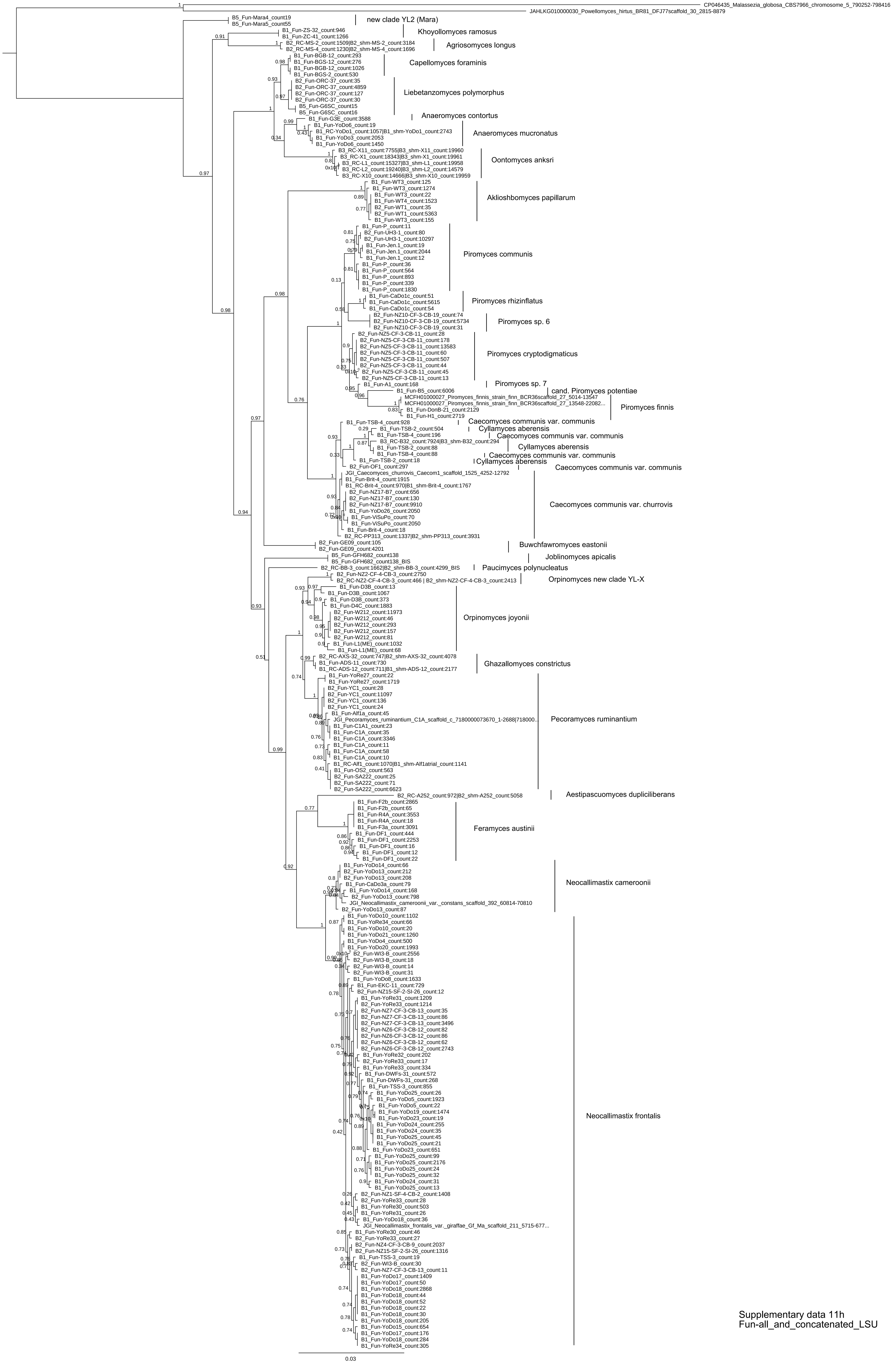

Supplementary data 11h
Fun-all_and_concatenated_LSU

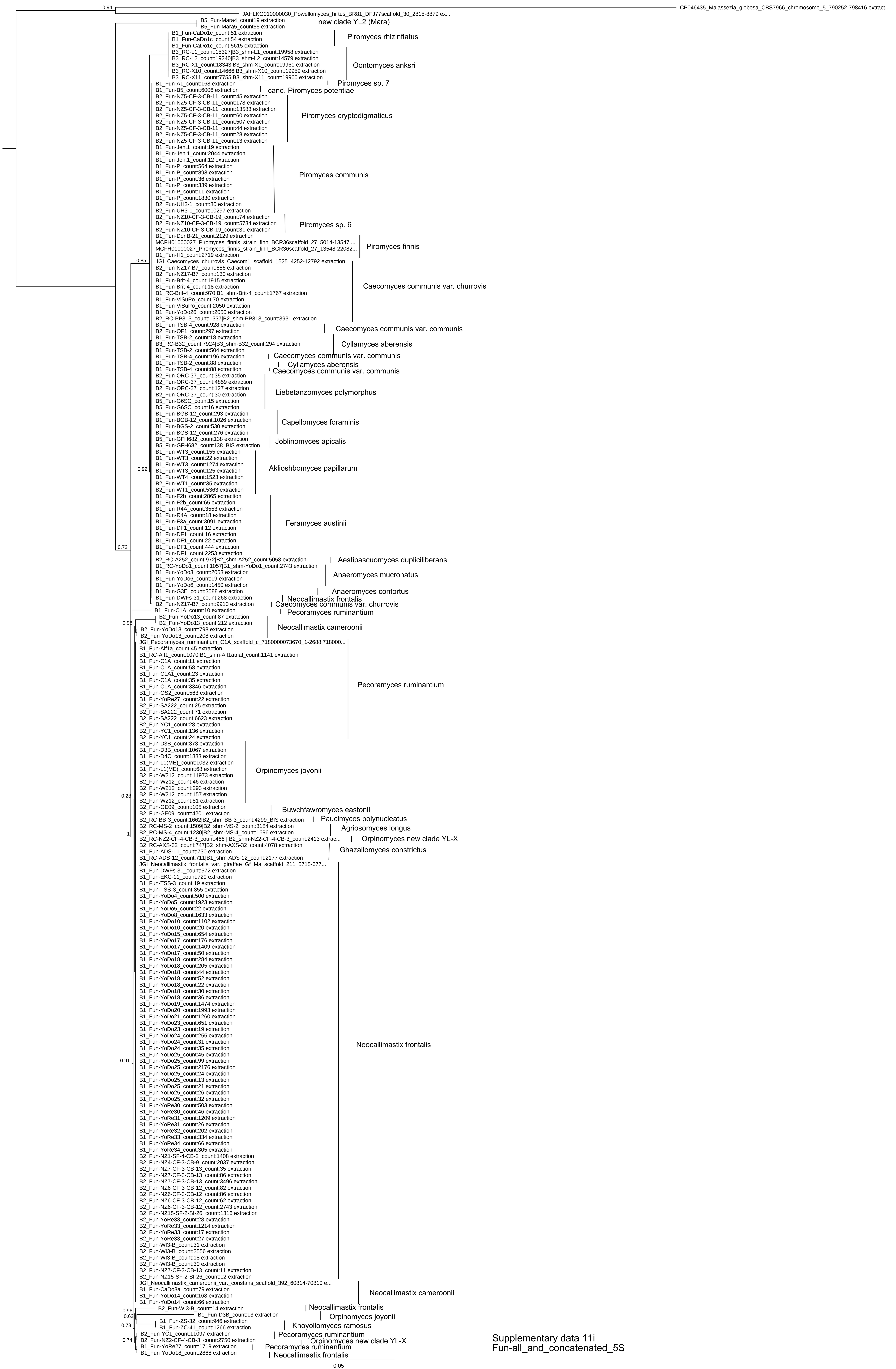

Supplementary data 11i
Fun-all_and_concatenated_5S

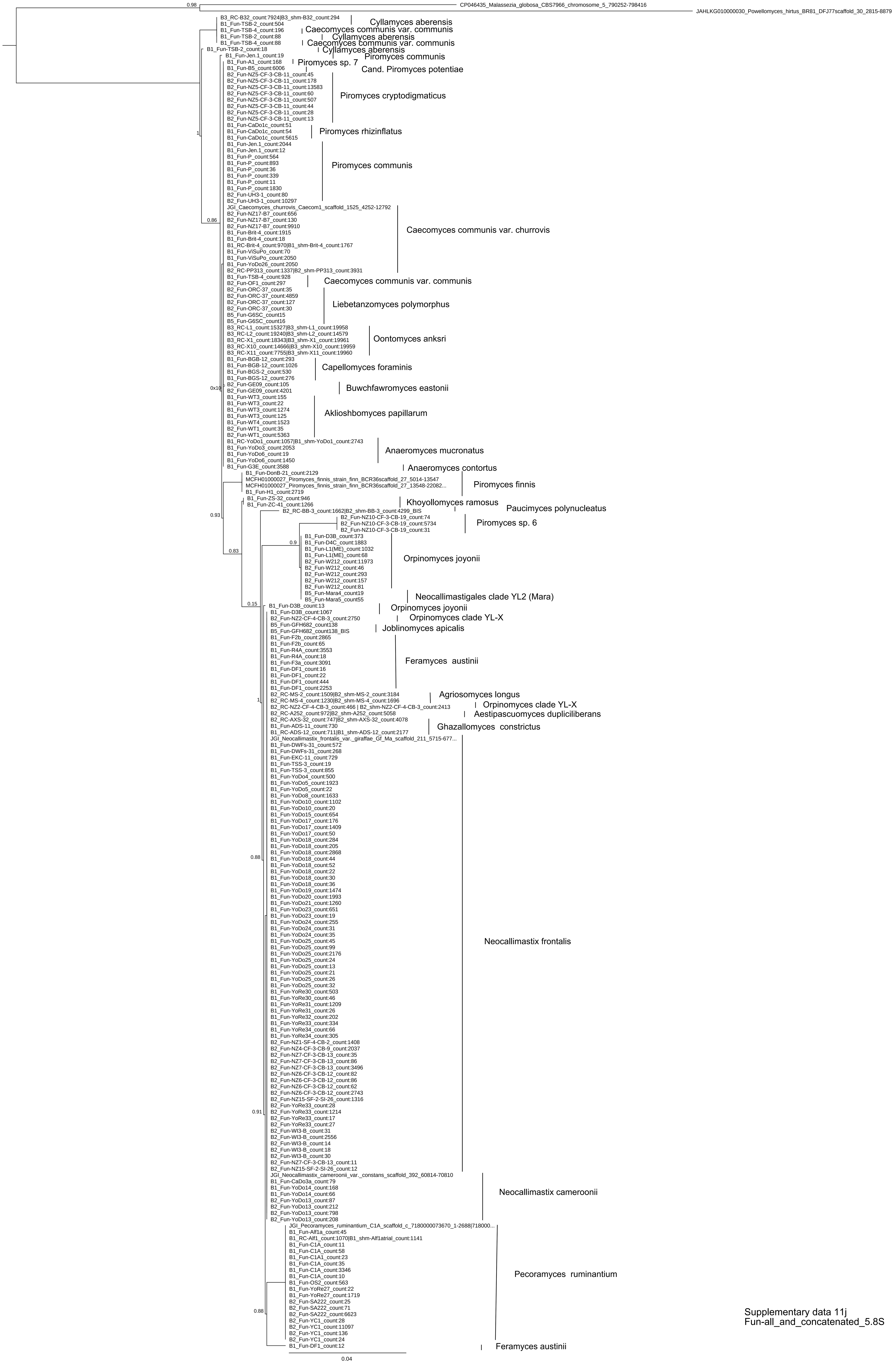

Supplementary data 11j
Fun-all_and_concatenated_5.8S

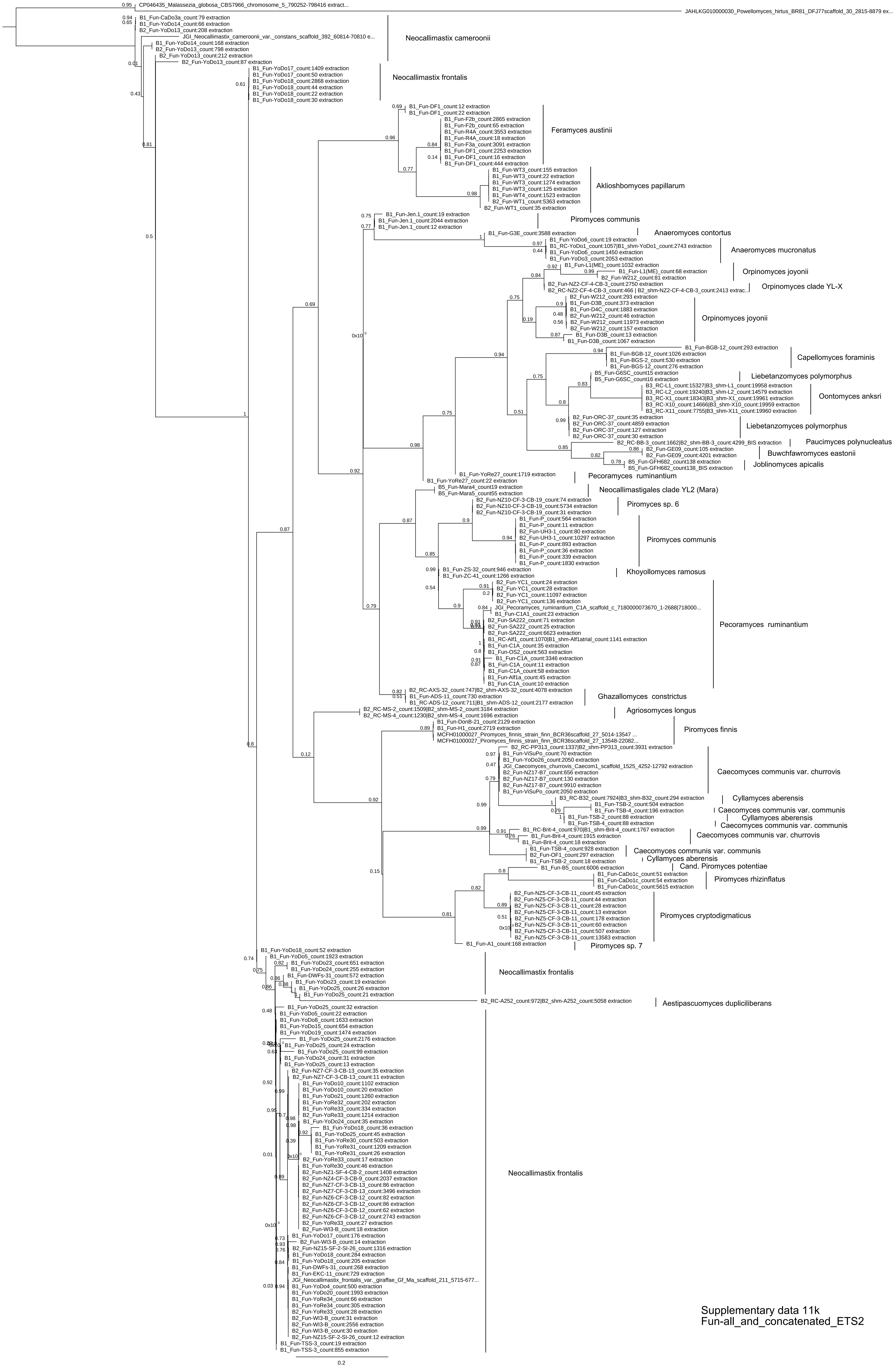

Supplementary data 11k
Fun-all_and_concatenated_ETS2

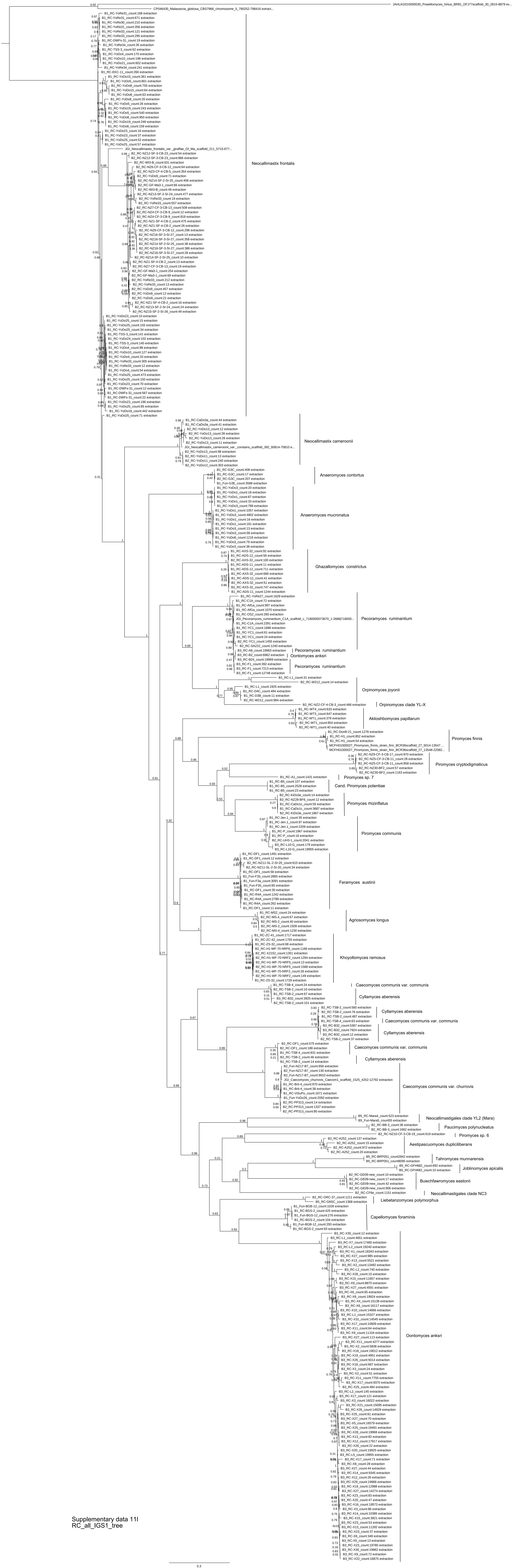

Supplementary data 11I
RC_all_IGS1_tree

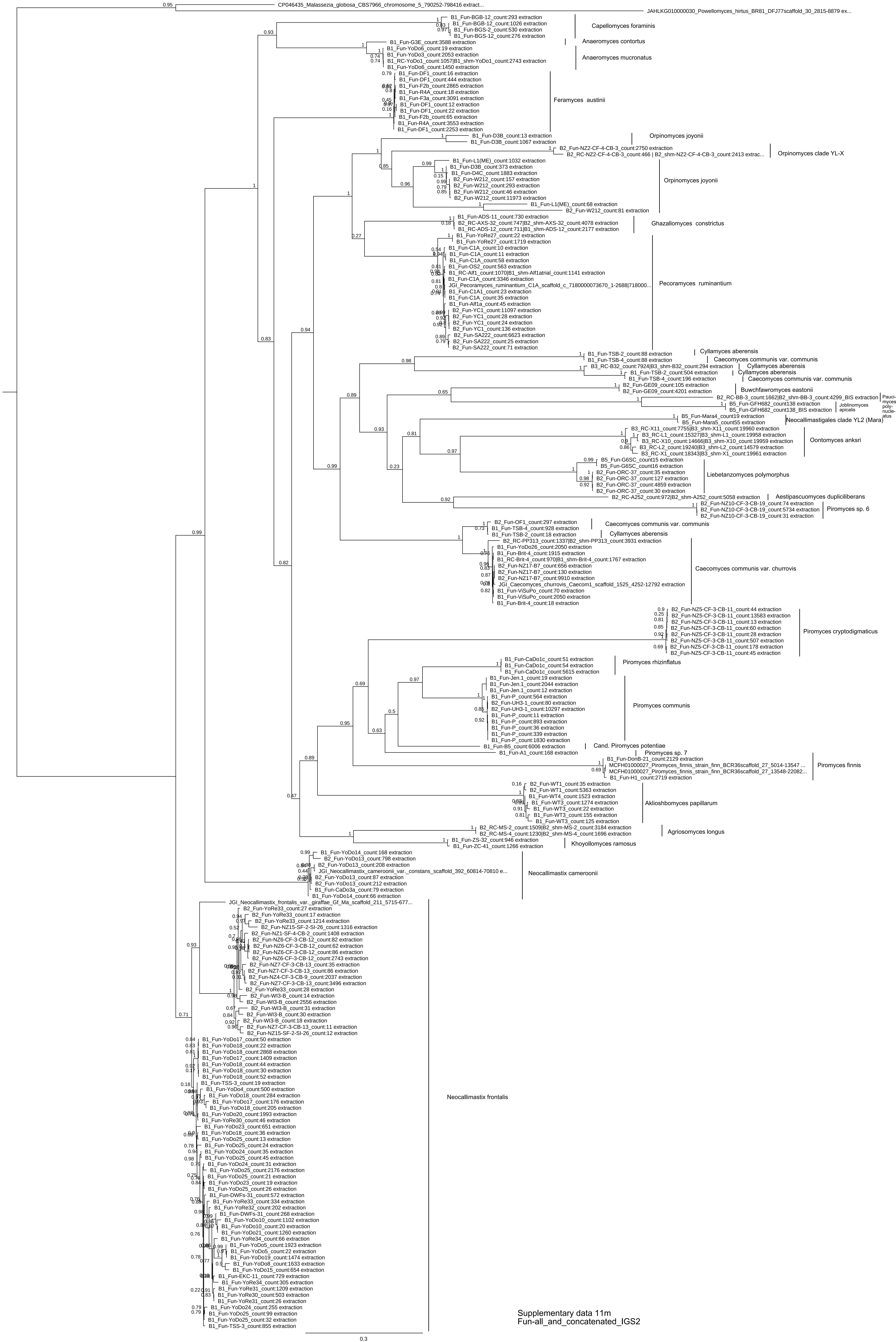

Supplementary data 11m
Fun-all_and_concatenated_IGS2

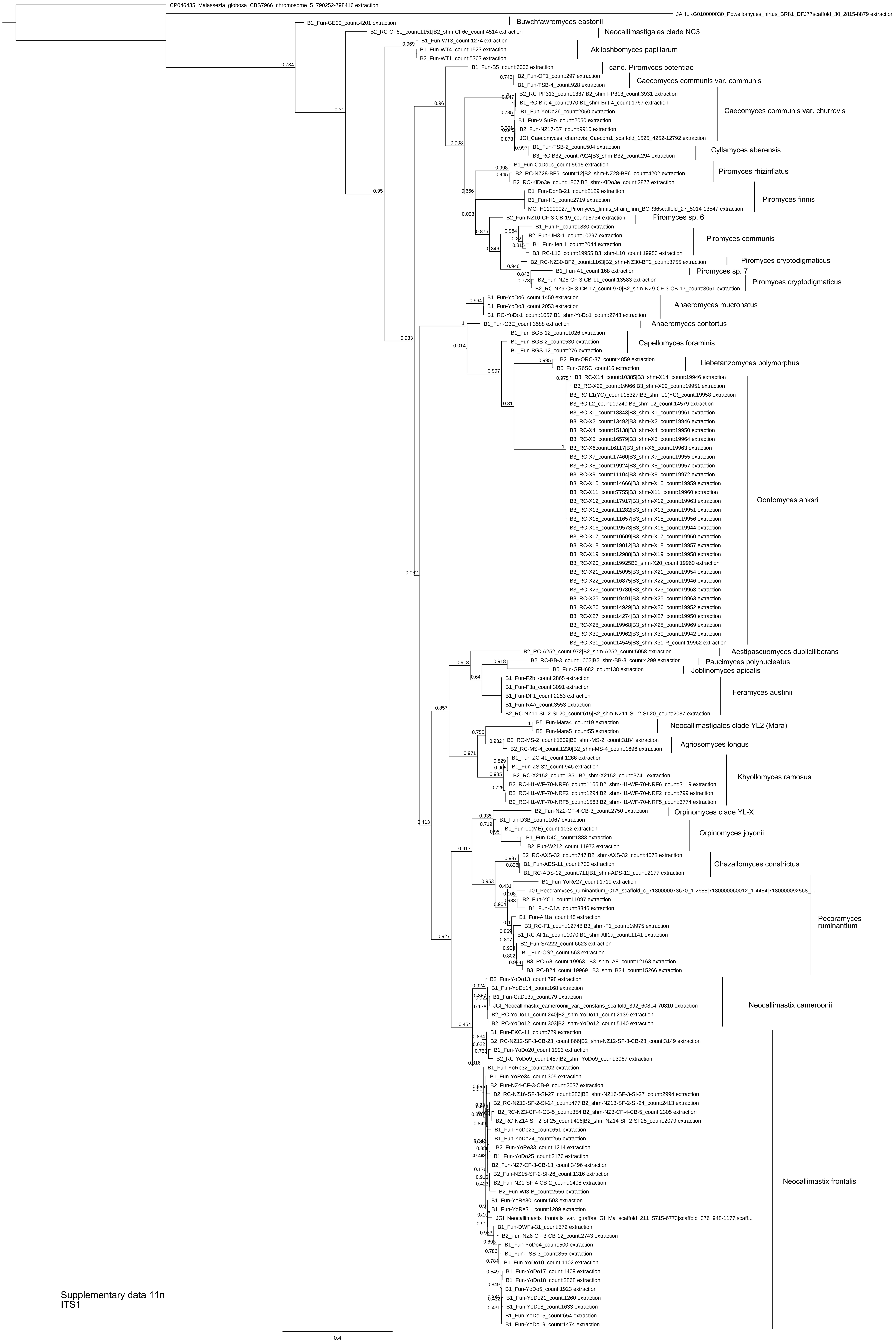

Supplementary data 11n
ITS1

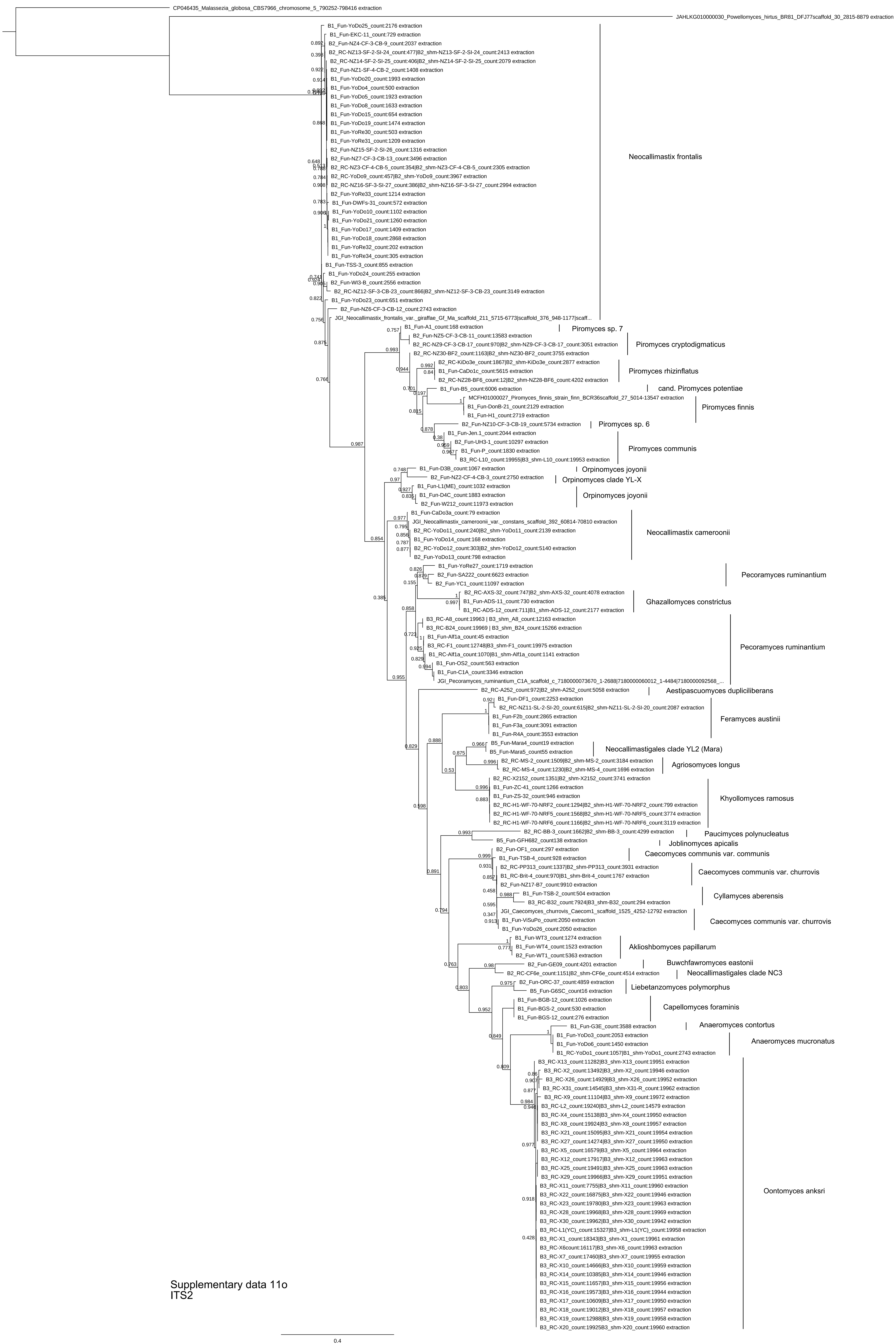

Supplementary data 11o
ITS2

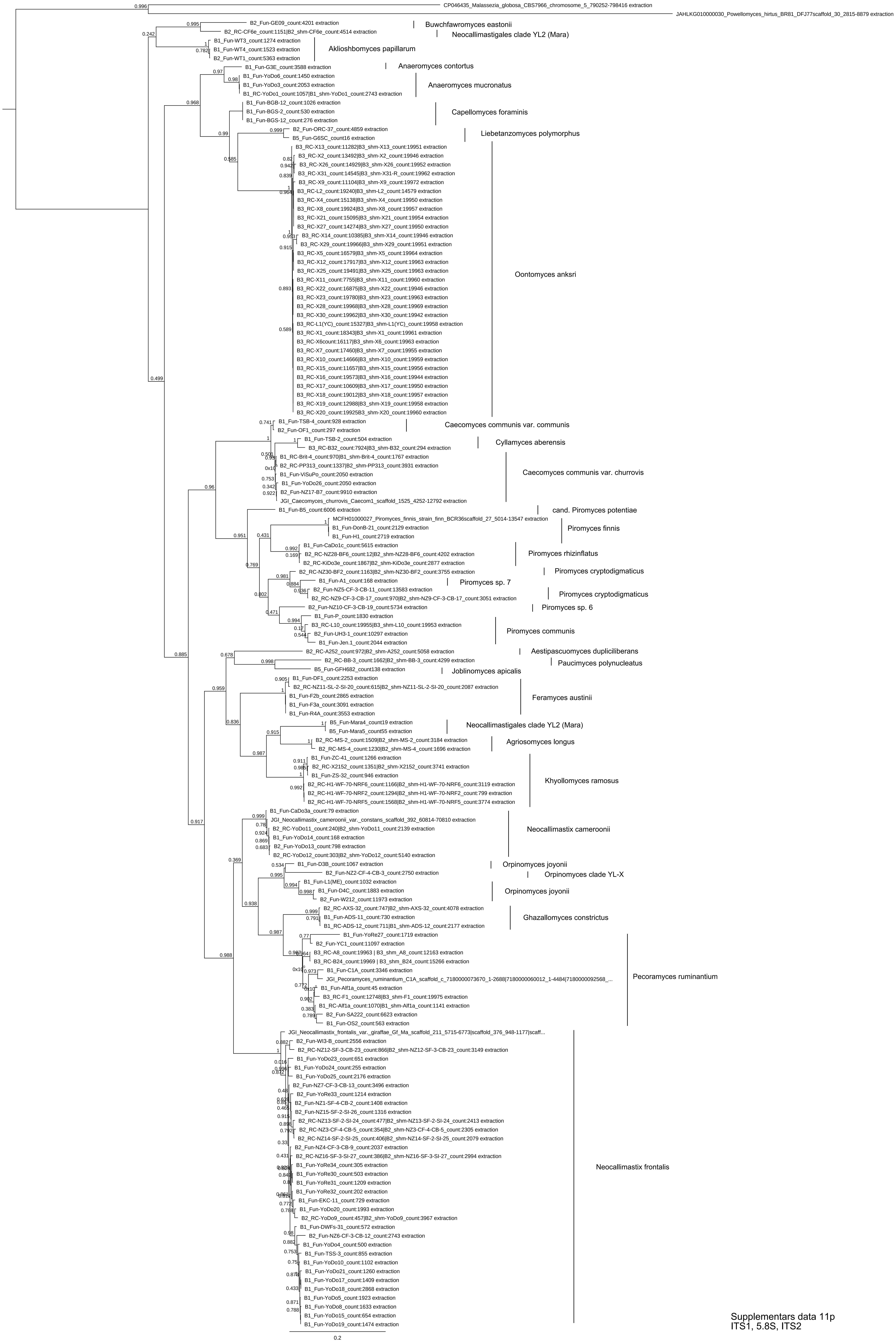

Supplementars data 11p
ITS1, 5.8S, ITS2

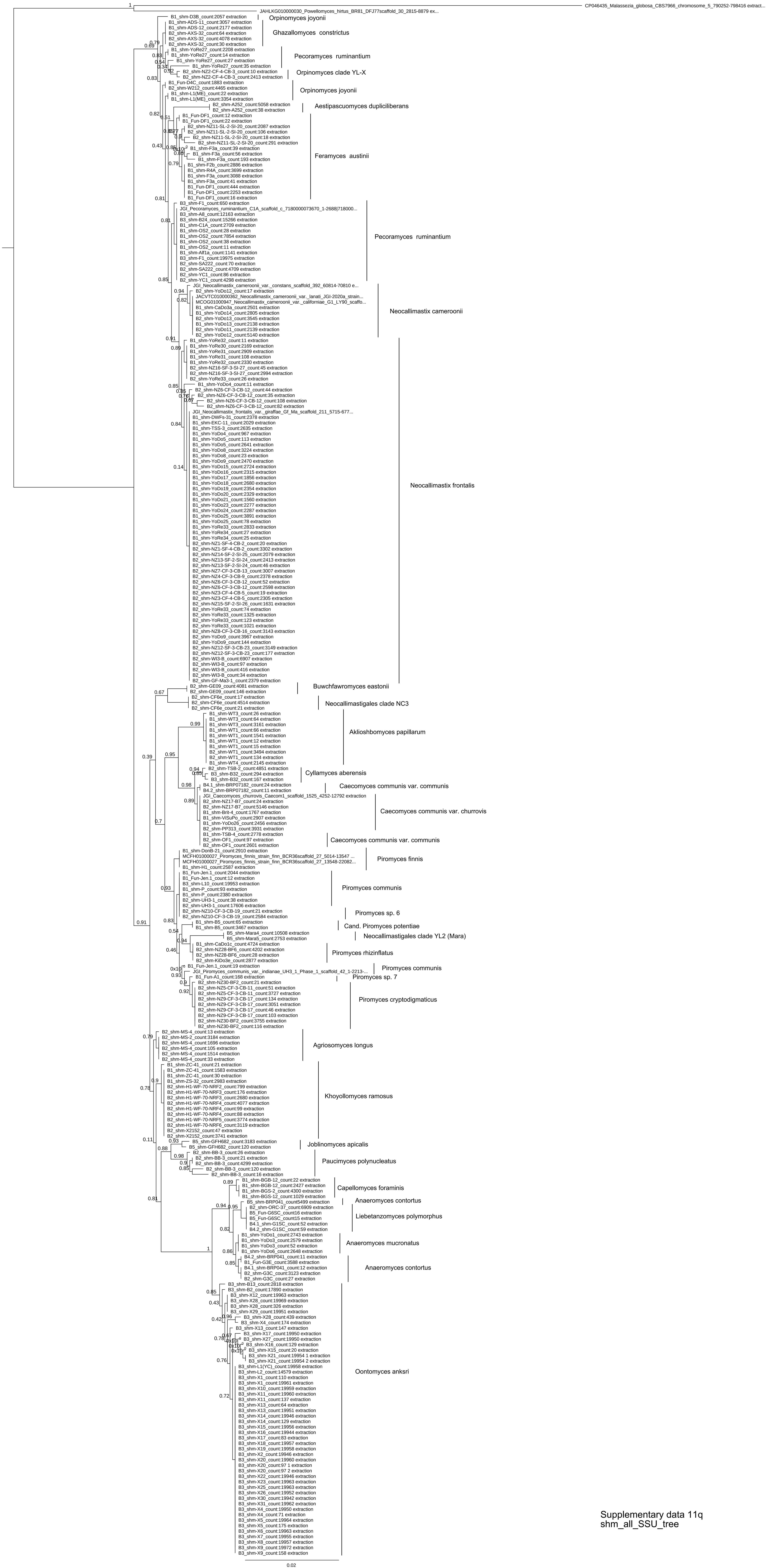

Supplementary data 11q
shm_all_SSU_tree

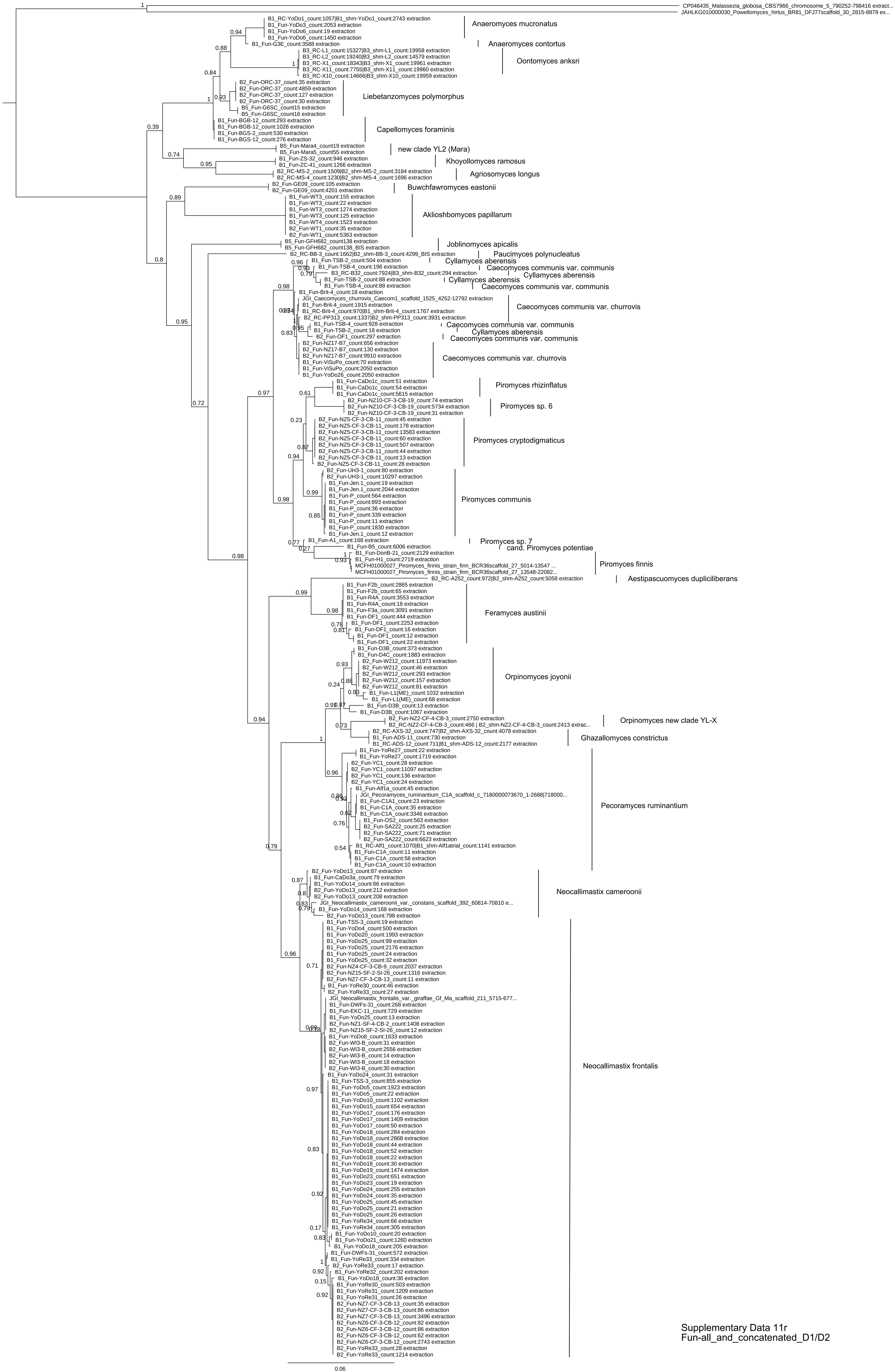

Supplementary Data 11r
Fun-all_and_concatenated_D1/D2

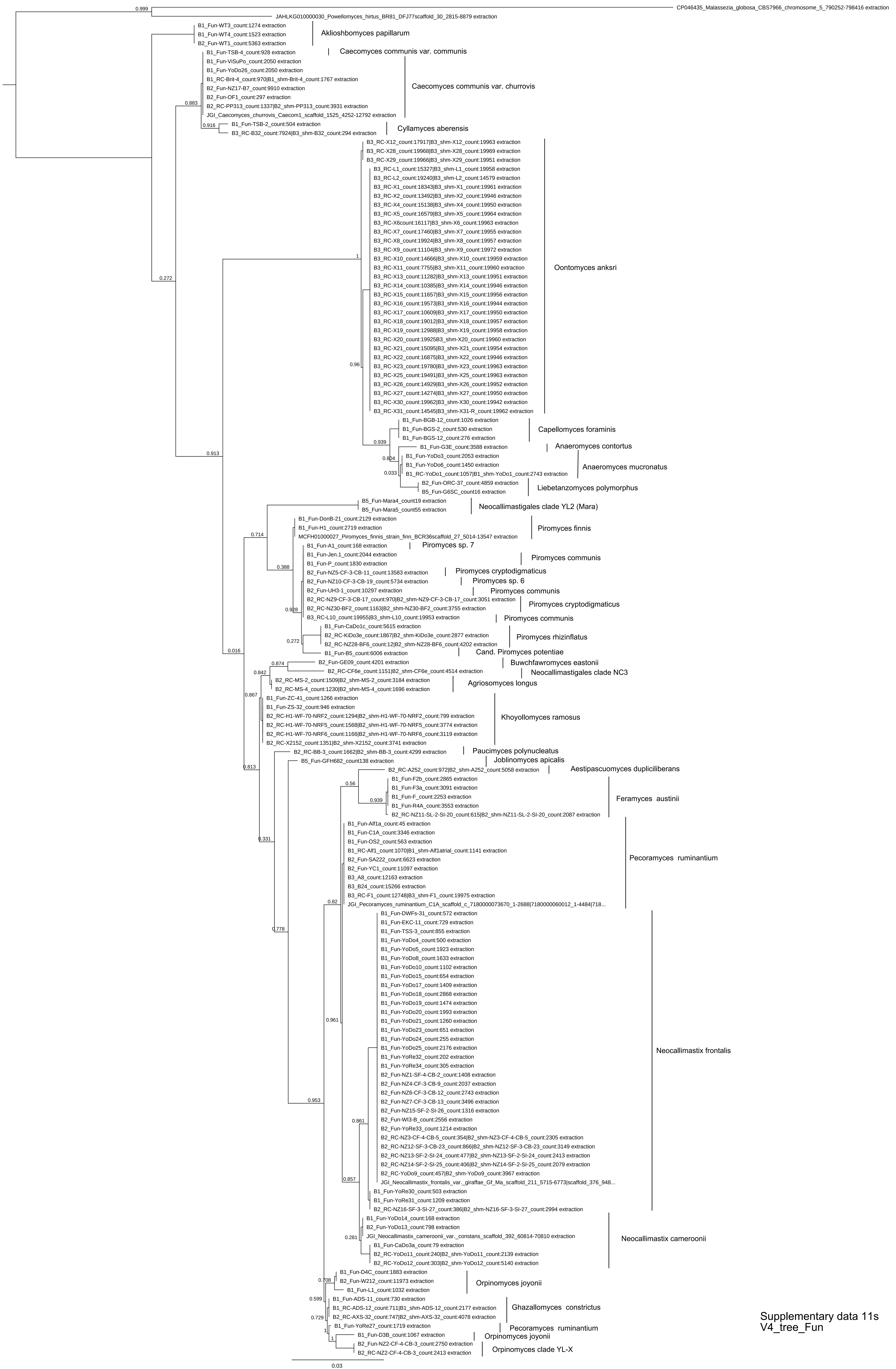

Supplementary data 11s
V4_tree_Fun

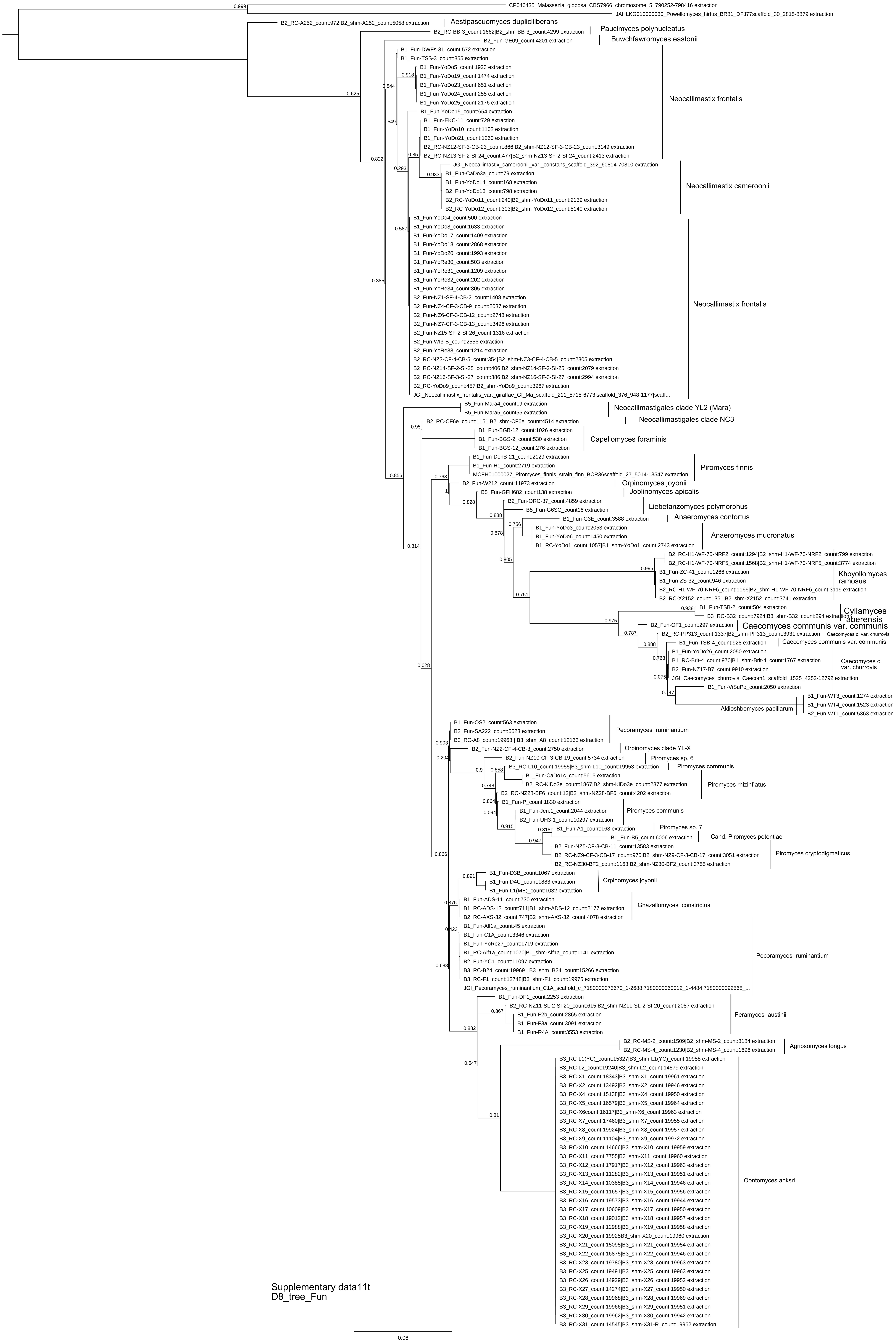

Supplementary data11t
D8_tree_Fun

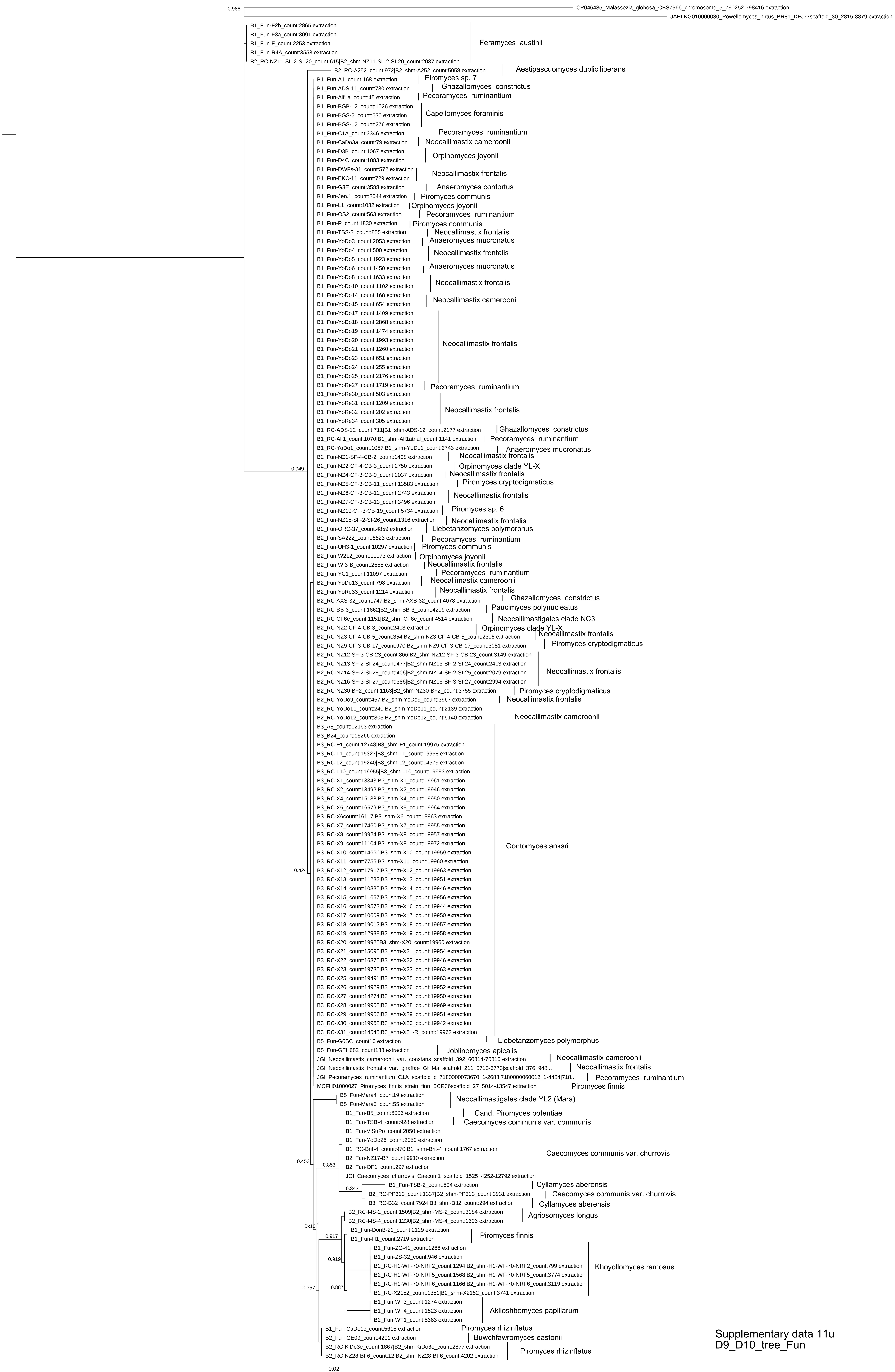

Supplementary data 11u
D9_D10_tree_Fun

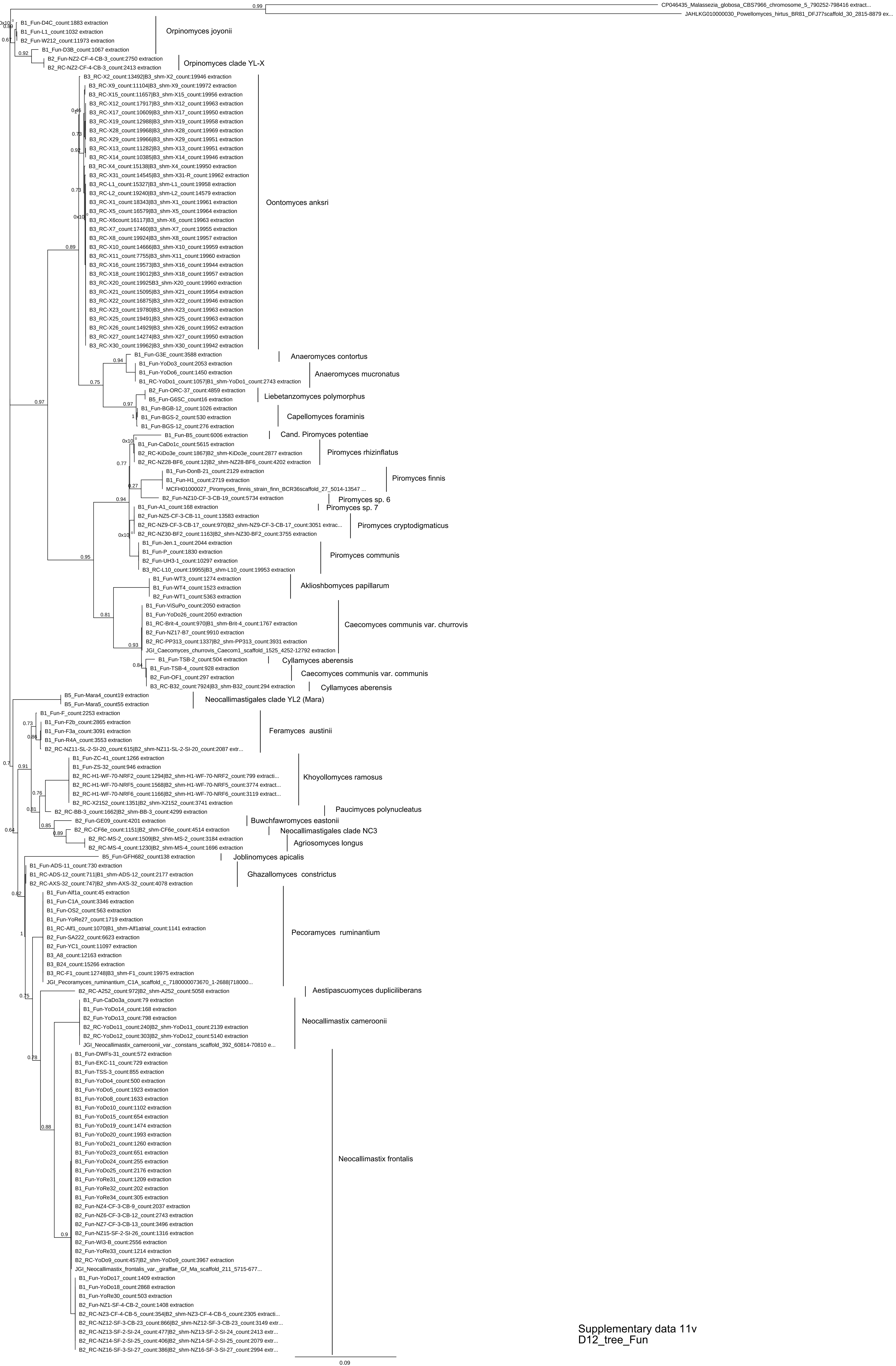

Supplementary data 11v
D12_tree_Fun
