# Supplementary material for: Full ribosomal operon sequencing of anaerobic gut fungi (phylum Neocallimastigomycota): insights on its markers and phylogenetic resolution
Source: IMA Fungus. 2026 Aug 26;17:e195921. doi: 10.3897/imafungus.17.195921 (PMC13539303; doi:10.3897/imafungus.17.195921)
Supplement: Supplementary file 1 [file imafungus-17-e195921-s001.zip › Supplementary_Data_1 to 14/Supplementary Data 3_Peak detection.pdf]

Supplementary Data 3: Peak detection

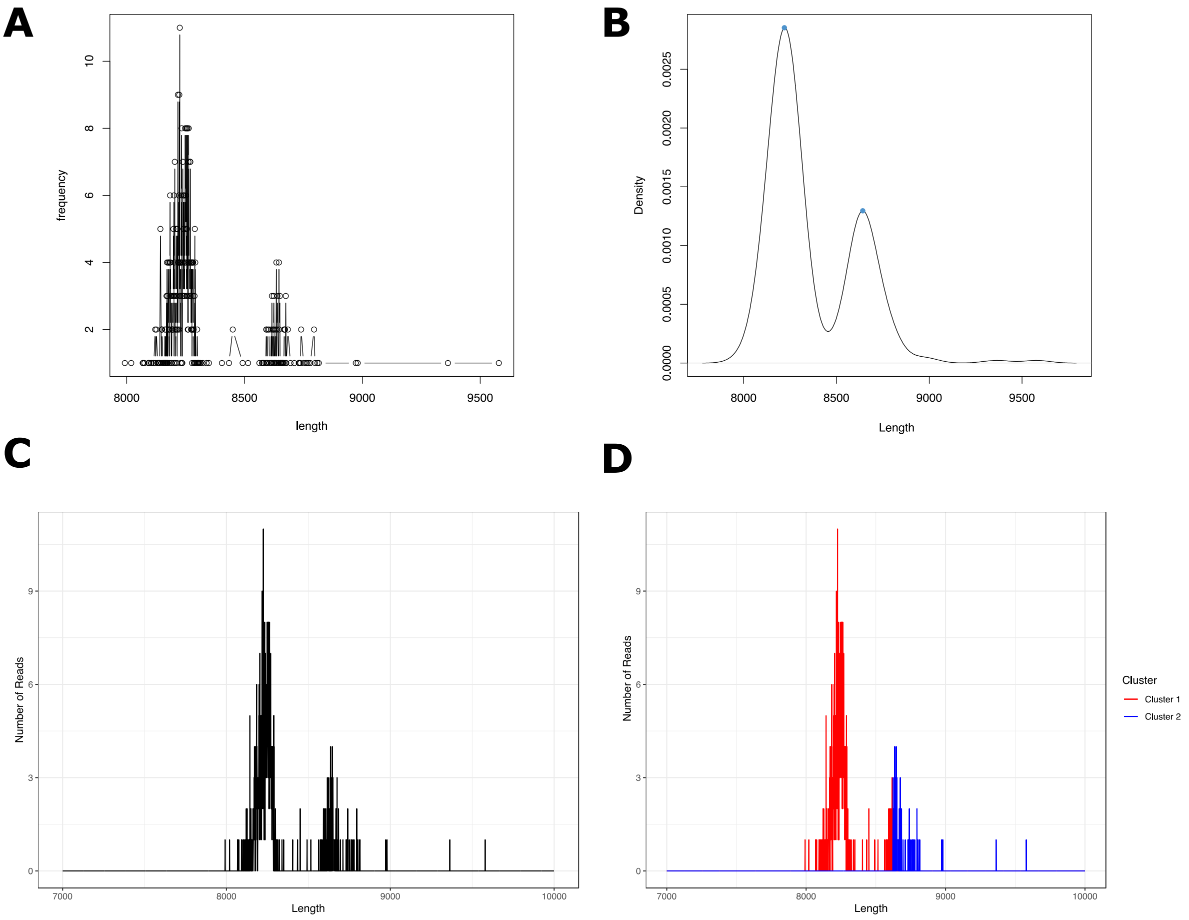

Supplementary Data 3: Peak detection from the read length distribution and comparison with the read length clustering approach. All sequences are from the sample Fun-Brit-4. A), read length distribution. B), density distribution based on the frequencies of each read length. C), read length distribution. D), two clusters are generated based on read length and are marked in red and blue.

As the length distributions are close to a normal distribution pattern, we used a kernel density estimation to smooth the data points (frequencies) and select the largest value within each curve (peak calling) (Supplementary Data 3. Graph B). Once the peaks were detected, the width of the peak was used to group the reads based on their lengths. Peaks with less than 10 reads were filtered out as there were not enough sequences to build a high-confidence consensus sequence.

The advantage of peak detection in comparison to the conventional clustering approach is that the grouping result matches the length distribution, whereas clustering is a distance-based approach which may have the result that the cut between clusters might not be located at the lowest point of the length distribution (Supplementary Data 3, Graph D).
