# Supplementary material for: Full ribosomal operon sequencing of anaerobic gut fungi (phylum Neocallimastigomycota): insights on its markers and phylogenetic resolution
Source: IMA Fungus. 2026 Aug 26;17:e195921. doi: 10.3897/imafungus.17.195921 (PMC13539303; doi:10.3897/imafungus.17.195921)
Supplement: Supplementary file 1 [file imafungus-17-e195921-s001.zip › Supplementary_Data_1 to 14/Supplementary Data 12_rrn primers map.pdf]

5'••• TCCTATTTTTTTGGAACTTTTTTTTCTCTTTGTCTAGGGTAGTTCCGTAGGGTTGGCAATAATGGATGGTTGATATGATGTAAGGTAGGTTGTCT 95

GACTTTGAATTTTTTTCGACTTCAAATTTTTTGGACATGTAAATTTTTTTTTGCATTATTGGACATTATTGGACATTATTGGACATTATTGGACA 190

TATTCGACATATTCGACATTTTGGACATCAAAATTTTTGGAATTATTTTTTTGAAATTAAACGTAGGGTTGCTTATTGGAATTTTTGGTCTTATT 285

TGGACTTTTTAGACTTTGACTATGAATTATTAATAAAATTAATTATGATTTATTATTTATTATTATTTATTAATAGATTAAATAAATATTATTAA 380

TAAATAATAAATTAATTATATTTATTTTATATATAGAGATTAATTATTATTTATTTATTATTTATAAGTAAATTTATTATTATTATTAATAGATT 475

ATTATTATTATTTTTTTATAAGTAAATTTATTATTATTTATTAATAGATTAATTATTATTTATTTATATTTATAAGTAAATTTATTATTATTTAT 570

TAGATTAATTATTATTTATTTATTATTTATTAAATTATTATTATTTATTAATAGGTTAATTATTATTTATTTATTATTTATAGTAATTTATTATT 665

ATTTATTAATAGATTAATTTTATTTATAAATAAATTTATTATTATTTATTATATTTATATATATTTTTATTTTTATTTTTATTATTTATAGTAAA 760

TTTATTTTTTTTTTTTTTTTTTTTTTTTTATTTTAAATCCTTTCAATCCATTACCCTTTATCTATTACTTACCAACCAATTTGGTCGGTTACGGCC 855

ATACACACTAGAAAACACCGGATCCCGTCCGATCTCCGAAGTTAAGCTAGTGATGGCGCAGTCAGTACTATGGTGGGGGACCACATGGGAATACT 950

GCGTGTCGTAATTTTTTTTTTTTTTTTTTATTTTTTTTAATAAAATAATGAACATTTATTATTAAATTGGACATGAGGGTTAGTTAATATCGAGTT 1045

TTTTTTAATATTTATGTCTTTGTATTATCATTTATTGAATAATTAATATAAAATATTATATATTTTATATATAAATCAATAAAAATCTTAACCCT 1140

TGGAATTAAATTTTTTAAATAAATTATATAAATTATTAAAAAATTATATATAATTATACCAAAATTATTATAAATTATAATAAAAAAAATAAAAA 1235

AATAATAAAAATAAATATAAAAATTAATTTTGCATAACCCTTACAATCACAAAAGTCCACTTTTAATTTTTTTTTTATAATAAATAATATAAAAA 1330

AAAAATAAAATTAAAAATAAAGGCCTTTTTATATATATAATAATTTTTTTTTTTTTATGAAAAAAATATTAAAATGTTATTCTAATTTTTTTAAAC 1425

ATTATTTCTTTTTATTTTTTATATTTTTACTTAATTTTTTAATGTAAATGTAAAATTGATCAATTTAAATTAACACTTAAATAAACATTTAATTA 1520

TTATAAATATTAACAAGAAATAAAAATAAATAAATAAATTTTTTTTTTTTTTTTTTTTTTTTTTATTTTTATTAATAATAAAATAAATATATAAATA 1615

AATTAACATTATTAAAAATAAAAAATACCCTTTATAATATTTTTTAGGTATAGAATACATTGAAATAATTAAATATAACTAAAAACAATTTTTTA 1710

AAAGTGGCCTTTAATAAAAATAATAAATAATAAAAAATAAATAATAAAAAAATAAATAATAAAAATATAAATAATAAAAAAATAAATAATAAAAA 1805

AATAAATAATAAAATATAAAATAAATAATAAAATAAATAATAGAAAATAAATAATAAGAAAAATAAATAATAGAAAAATAAATAATAGAAATAAT 1900

AAATAATAGAATAGTAAATAATAGAAAAATAAATAATAGAAATAATAAATAATAAATAATAAATAATAGAAATAATAAATATAAATAATAAATAA 1995

TAGAAATAATAAAATAAATAATAAATAATAAAAAAATAAATATAAATAATAAATAATAAAAAAATAAATAAAAATAATAATTATAAATATAAAAA 2090

ATAAAAATAATAGTTAATAATTAATAAAATAAAAAAATATAAATAATAAATATAATAAATATAAATAATAAAAATAAAAATAAAAATAAAAATAA 2185

AAATAAAAATAAAAATATAAATAAAAATAAAAATAATAATAATAATTAATAATTAATAAAAATAAAAATAAATAATAAATAATAATTATAATAAT 2280

AATATTATTTTTATTATTATTATTATTATTATTATTATTATTTTTATTATTATTTATTATTATTTATTATTTATTATTTATTTTTATTAATTAATAAA 2375

TTATTATTAATATTATTATTATTTAATTATATATATATCTTATATATATATTTTTTATATTTAATTTATATAAAAAAAAAAAAAAAAATTTTTTTTA 2470

ATTTTTTTTTACAAAATTAAATTTAATAGTTCCATAATTCTATTAATATTTTATTATTTTAAAAAAAAAAAAAAAAAATTTTTTTTTTGGCTTTA 2565

AAATTAAATTTTGATTAAAAATTTAAAAATTTAAATTTTTAAAAAAAACGCATATCCGAAATTTTATTTTATAATTAATTTAATATAAATCTATA 2660

ATTAAATTAAAAATTTAAATTTTTTGGACCAATCGATTTTTAAATAGAAAAATTTTTCTACTTTGACAAATAAAATTTGCTTTTGCTAAGTGGGT 2755

AGTCATTTTTTTTGGTTTTTTATCTATGTGATGAAAGAAAAAAAAAAATTAGAAATAATAAAATTTCTAATTTGGATTTTTTTTCATTGCAAGCCT 2850

TTTATTTTATAAAAGGTTTAAATTTTCTAGTGATATACTACGGTAAATTTGTTTGGGGTAACTATTTTAATAATAGCGAACGAAAGTTTTAAATC 2945

CCTATAACAAGTTATTGTAGTACTTGTGAAAACTCATAGTGGTACATTGAGTACGTATCTATGGTAGGTTAGTAACATGTGTATTCATAGGAAAT 3040

AGTCCTACAACATCTGGATAATTTTTCTTGTGAAATACTATATAATTATACTGCTTTGTAAAAAGCTAGGAAATTATAATAGAATTCACATTATA 3135

AATTTATTTTTATAAAAGTTTCATAATTCATTTTTGAATTAATGAAAATATTTTATAATAATAAATTTAGGAAAACTTTTGAAAGATGTGGCAGC 3230

TTAATGTAATCATAATAGTTATATGATTTAATTAATAGTAGATAAAGATTGCAATGAATTTTTTATTGTAATAGTTATTTACATGGTTAATTTTA 3325

TTTTATAATTATTATTGATGATATTAAATTTTGATATCATGTAATTTATCTTTTTTTTTAATAAACGTTTTATCGTTTATTATATAAGAAAAAAGAT 3420

NS1 (F)_18S
GTAGTCATATGCTTGTCTC

NS1sh_rRNA shm
CAGTAGTCATATGCTTGTC

AGAATAAAATTTTATTTTATTCTAACTTTTTTTTTAAAGATAGTTACCTGGTTGATCCTGCCAGTAGTCATATGCTTGTCTCAAAGATTAAGCC 3515

GTCATCAGTATACGAACAG
NS1rc_IGS RC

CATCAGTATACGAACAGAG
(F)_IGS

ATGCATGTCTAAGTATAAACAATTCTATACTGTGAAACTGCGAATGGCTCATTAAATCAGTTATAGTTTATTTGATAGTACCATTACTACTTGGA 3610

TAACCGTGGTAATTCTAGAGCTAATACATGCGTAAAAACCCGACTTCTGGAAGGGTTGTATTTATTAGATAAAAAACCAACCTGGGCAACCAGTT 3705

TTTTGGTGATTCATAATAACTTTTCGAATCGTATGACTTTACGTCGACGATGGTTCATTCAAATTTCTGCCCTATCAACTTTCGATGGTAGGATA 3800

GAGGCCTACCATGGTTTTAACGGGTAACGGAGAATTAGGGTTCGATTCCGGAGAGGGAGCCTGAGAAACGGCTACCACATCTAAGGAAGGCAGCA 3895

GGCCTCTCCCTCGGACT
309e_18S

GGCGCGCAAATTACCCAATCCTGACACAGGGAGGTAGTGACAATAAATAACGATACAGGGCTATTTTAGTCTTGTAATTGGAATGAGAACAATTT 3990

515F V4/V5
GTGCCAGCMGCCGCGGTAA
AAACCCCTTATCGAGGAACAATTGGAGGGCAAGTCTGGTGCCAGCAGCCGCGGTAATTCCAGCTCCAATAGCGTATATTAAAGTTGTTGCAGTTA 4085
CGTTCAGACCACGGTCGTCGG
NS2 (R)_18S
GTCGKCGGCGCCATTAWG
519_18S

AAAAGCTCGTAGTTGAATTTTGGACTTGACTGTACGATTCTACATCTTGTAGCTATTGTACTGGTCTTGTCTTTCCTTCTAGCTAACCTTTATGC 4180

ACTTCATTGTGTGTACTGGGGATCTAGGACTTTTACTTTGAAAAAATTAGAGTGTTTAAAGCAGGCTTATGCTTGAATACATTAGCATGGAATAA 4275

TAGAATAGGACTTTGGTTCTATTTTGTTGGTTTCTAGGACCGAAGTAATGATTAATAGGGATAGTTGGGGGCATTAGTATTTAATTGTCAGAGGT 4370
GTCTCCA
686e_18S

GAAATTCTTAGATTTATGAAAGACTAACTTCTGCGAAAGCATTTGCCAAGGATGTTTTCATTAATCAAGAACGAAAGTTAGGGGATCGAAGACGA 4465
CTTTAAGA
686e_18S

AF-SSU F_18S
CTAGGGATCGGACGACGTTT
TCAGATACCGTCGTAGTCTTAACCATAAACTATGCCGACTAGGGATCGGACGACGTTTAATTATTGACTCGTTCGGCACCTTATGAGAAATCAAA 4560

GTTTTTGGGTTCCGGGGGGAGTATGGTCGCAAGGCTGAAACTTAAAGGAATTGACGGAAGGGCACCACCAGGAGTGGAGCCTGCGGCTTAATTTG 4655
TTTGARTTTMYTTAACYGCC
926R V4/V5

ACTCAACACGGGGAAACTTACCAGGTCCAGACATAGTAAGGATTGACAGATTGAGAGCTCTTTCTTGATTCTATGGGTGGTGGTGCATGGCCGTT 4750

CTTAGTTGGTGGAGTGATTTGTCTGGTTAATTCCGATAACGAACGAGACCTTAACCTGCTAAATAGTTACGCGAATATTTTATTCGTGGTTTAAC 4845
TGGAATTGGACGATTTATCAATGCGCTTA
AF-SSU probe_18S

TTCTTAGAGGGACTATAGATTTTTAATCTATGGAAGTTTGAGGCAATAACAGGTCTGTGATGCCCTTAGATGTTCTGGGCCGCACGCGCGCTACA 4940

CTGATGAAGTCAACGAGTTTCATCCTTGATCGGAAGGTCCGGGTAATCTTTTGAAACTTCATCGTGCTGGGGATAGTCCCTTGCAATTTTTGGAC 5035
GTAGGAACTAGCCYTCCAGG
AF-SSU R_18S

GM1 (F)_ITS
TGTACACACCGCCCGTC
(F)_ITS1
TGTACACACCGCCCGTC
TTAAACGAGGAATTCCTAGTAAGCGTGAGTCATCAACTCGCGTTGATTACGTCCCTGCCCTTTGTACACACCGCCCGTCGCTACTACCGATTGAA 5130
TGTGTGGCGGGCA
1400_18S_no brackets

JB206 (F)_ITS (1)
GGAAGTAAA

JB206 (F)_ITS
GGAAGTAAA

ITS5 (F)
GGAAGTAAA

qRT-PCR-fungi (F)_ITS1
GAGGAAGTAAA

(F)_SSU and ITS1
GAGGAAGTAAA

ITS1F_ITS1
CTTGGTCATTTAGAGGAAGTAA

ITS1F (F) (1)
CTTGGTCATTTAGAGGAAGTAA

TGGCTTAGTGAATCCTTCGGATTGGCTATTTTTTTCTGGCAACAGAATTAAACTGCTAAAAAGTTGGGCAAACTTGGTCATTTAGAGGAAGTAAA 5225

JB206 (F)_ITS (1)
AGTCGTAACAAGG

ITS1F (F)
TCCGTAGGTGAACCTGCGG

JB206 (F)_ITS
AGTCGTAACAAGG

ITS 1 (F)_ITS1
TCCGTAGGTGAACCTGCGG

ITS5 (F)
AGTCGTAACAAGG

ITS 1 (F)
TCCGTAGGTGAACCTGCGG

qRT-PCR-fungi (F)_ITS1
AGTCGTAACAAGGTTTC

MN100 (F)_ITS1
TCCTACCCTTTGTG

(F)_SSU and ITS1
AGTCGTAACAAGGTTTC

(F)_ITS1after
TCCTACCCTTTGTG

AGTCGTAACAAGGTTTCCGTAGGTGAACCTGCGGAAGGATCATTAAAAAATATCGCTCGATTGAGAGTGATTTAATAATCATCCTACCCTTTGTG 5320

CATCCACTTGGACG
1511e_18S_no brackets

TTAGTAGGATGGGAAACAC
(R)_SSU and ITS1

TTAGTAGGATGGGAAACAC
qRT-PCR-fungi (R)_ITS1

MN100 (F)_ITS1
AATTTG

(F)_ITS1after
AATTTG

AATTTGTTGTTTGAAATAATTTTTTTTTGACGTGGAATAAAAAAAAAAATTATTTATGGTTTTGTCTATCCCTAAAATTGGTTTGTTTGTTGTAA 5415

TTAAAC
(R)_SSU and ITS1

TTAAAC
qRT-PCR-fungi (R)_ITS1

AAGACAAGTGAATCATTTAGGGAATAGGCTTTCCTAAATAATTGATTTTTTTTCCAATGTCTTAAAAGACCTTTTTTTAAAATTAAACTTTTGTA 5510

Neo QPCR (F)_5.8S
TTGACAATGGATCTCTTGGTTCTC

TTCATTTGTCTAAAATATTTTTTAAAATAATTTAAAAACAACTTTTGACAATGGATCTCTTGGTTCTCGCAACGATGAAGAACGCAGCAAAATGC 5605

TATTAWATTTTTGTTGAAAACTGTTA
ITS400Rw (R)

GCGTTGCTACTTCTTGCGTC
MNGM2 (R)_ITS1

ACCTAGAGAACCAAGAGC
Neo 5.8S (R)

GATAAGTAATGTGAATTGCAGAATACGTGAATCATCAAATCTTCGAACGCATATTGCACTTTTTTAGTTTACTAAAAAAGTATGTCTGTTTGAGT 5700

TTAGAAGCTTGCGTATAACGTG
Neo QPCR (R)_5.8S

TTAGAAGCTTGCGTATAACGTG
Neo QPCR (R)_ITS1

ATCAGTAAAATATTCTCATAAAAACATTTTTTGTTTTTATGGTTATGAGTGTTTTACAACGATAAACAAGTTGTTTCACTTTAAATTCATTTTAA 5795

TTTAAGACCATTTATGGTTTTAATAAAAAATTGGATAAAATTTTCCCGTAAAAAGGAATAAACTTTTCAGTTTTTTAATTATTACCAACAAACGG 5890

NL1 F D1/D2
GCATATCAATAAGCGGAGGAAAAG

TCCGATTTAAAAGGAAATTTTACTTAAATAACTTGATCTCAAATCAGATAAGAGTACCCGCTGAACTTAAGCATATCAATAAGCGGAGGAAAAGA 5985

GTCTATTCTCATGGGCGACTTG
EminITS4 (R)

CGTATAGTTATTCGCCTCCT
ITS 4 (R)

AF-LSU (F)
GCTCAAAYTTGAAATCTTMAAG

AACTAACAAGGATTCCCTTAGTAACGGCGAGTGAAGCGGGAAGAGCTCAAATTTGAAATCTTCAAGGTTCTACCTTGACGAATTGTAGTTTAAAG 6080

AAGTGTTTTCTGTTAGATGAGTAGCAAAAGTTCCTTGGAATGGGACATCATAGAGGGTGAGAATCCCGTATTTGGTTATTCATTCTAGCTTTGTG 6175

ATACACTTTCAAAGAGTCGGATTGTTTGGGAATGCAGTCCAAAATGGGTGGTAAATTTCATCTAAAGCTAAATATTGGCGAGAGACCGATAGCGA 6270

EnvS F D2
GCGTTTRRCACCASTGTTGT

ACAAGTACCGTGAGGGAAAGATGAAAAGAACTTTGAAAAGAGAGTTAAACAGTACGTGAAATTGTCAAAAGGGAAGCGTTTGACACCAGTGTTGT 6365

EnvS F D2
T

TTTTCCGAAAATCAATTACGAATGGCTTGATTTTGTGTTGTATTGACCTTATCAGCTTTCTGCACTTTTTTCGAGTCTTTCTTAATGCACTTTTC 6460

TTACGTGAAAAR
AF-LSU (R)

GGTTAACAAGTCAACATCAATTTCTTTTGTTGTAAAAGAGTATTCGGAAGGTGGCTTTCTTTTCGGAGAAAGTGTTTATAGCCCTATACTTCTAC 6555

YMAATTGTTC
AF-LSU (R)

AATCGGAGAGATTGAGGTCTGCAGCGTAGATCCTTCGGGGTTAGATACACTTGTGCTAAGTTGTAGCTTTTCATAGACAACTTGTTGACTATGTT 6650

TAGTCATGCGGCTTACTCGCTTGTGCTATTTTACCTACGCTTAGGATGTTGACAAAATGGTTTTAAACGACCCGTCTTGAAACACGGACCAAGGA 6745

ATGGATGYGAATCCTACAACTG
EnvS R D2

GGCAGAACTTTGTGCCTGG
NL4 R D1/D2

GTCTAACAAATATGCGAGTATTTGAGTGGTAAACTCATATGCGTAATGAAAGTGATTTTGGTGGGATTTTCGATGCACCATCAACCGGTCAGGAT 6840

TTTTTATGAGCGATCTGAGTCTAAGCATATTTGTTGGGACCCGAAAGATGGTGAACTATGCCTGAATAGGGTGAAGTCAGAGGAAACTCTGATGG 6935

AGGCTCGTAGCGGTTCTGACGTGCAAATCGATCGTCAAATTTGGGTATAGGGGCGAAAGACTAATCGAACCATCTAGTAGCTGGTTCCCTCCGAA 7030

GTTTCCCTCAGGATAGCAGAAACTCAATCAGTTTTATGAGGTAAAGCGAATGATTAGAGGTCTTAGGACTGAAACAGTCTTAACCTATTCTCAAA 7125

CTTTAAATATGTAAGAAGCTTAAGTTACTTAACTTTTTTGAACTTGGGCATTTGAATGAGAGTTTCTAGTGGGCCATTTTTGGTAAGCAGAACTG 7220

GCGATGCGGGATGAACCGATCGTGGGGTTAAAGTGCCGGAATACACGCTCATTTAGACACCACAAAAGGTGTTGGTTCATCTAGACAGCAGGACG 7315

GTGGCCATGGAAGTCGGAATCCGCTAAGGAGTGTGTAACAACTCACCTGCCGAATGAACTAGCCCTGAAAATGGATGGCGCTCAAGCGTGTTACT 7410

CATACCCCACCGTTAGGGTAAAAGCGAAGCTCTAACGAGTAGGAGGGCGTGGAGGTTTGTGAAGAAGCCTAGGATGTGAATCCGGGTGAAACAGC 7505

CTCTAGTGCAGATCTTGGTGGTAGTAGCAAATATTCAAATGAGAACTTTGAAGACTGAAGTGGAGAAAGGTTCCATGTGAACAGCAGTTGGACAT 7600

GGGTAAGTCGATCCTAAGAGATAGGGAAGCTCCGTTTTAAAGTGAACGATTTTTCGTTCCACCTTATCAAAAGGGAATCCGGTTAATATTCCGGA 7695

ACTAGGCTATGGATATTTATTACGGTAACGTAAATGAACTAGGAGACACCAGTAGGAATCCTGGGAAGAGTTATCTTTTCTTTTTAACAATCTAA 7790

TCACCCTGAAATCAGATTAACTGGAGAAAGGGTAGTATGGTTGGCAAAGCATTCCTCTTTTGGAATGTCCGGTGCGTTCCTAACGGTCCTTGAAA 7885

ATCCTGGGGAAATAATAATTTTCATACCTAGTCGTACTCATAACCGCAGCAGGTCTCCAAGGTTAACAGCCTCTAGTTGATAGAACAATGTAGAT 7980

GA20 F D8
GTAACTTCGGGAWAAGGATTGGCT

AAGGGAAGTCGGCAAAATAGATCCGTAACTTCGGGATAAGGATTGGCTCTAAGGGTTGGGTATATTGGGCCTTTGTTCTGCAGCTTTGAGACCGA 8075

CTTGAGGACTTCTGGCCTTCACGGGCTGGAGGACTTCTTGTTGGATTCGAGGTGAACGAGCTCTGGCATGTCGGTTTCGGCCGGCGTCCGGTATA 8170

RCA95rc_IGS RC
CTGACTGTCTAATTAAAACATAG

F-rOP-F_FUN
CTGACTGTCTAATTAAAACAT

CAATTAACAACCAACTTAGAACTGGTACGGACAAGGGGAATCTGACTGTCTAATTAAAACATAGCATTGCGATGGCCAGAAAGTGGTGTTGACGC 8265

ATGCCTGTTCCCCTTAGACT
F-rOP-R_FUN

CCCTTAGRYTGACADATTAATTT
RM5 R D8

GACTGACAGATTAATTTTGTATC
RCA95m_rRNA shm

AATGTGATTTCTGCCCAGTGCTCTGAATGTCAAAGTGAAGAAATTCAACCAAGCGCGGGTAAACGGCGGGAGTAACTATGACTCTCTTAAGGTAG 8360

GLD8_421F
ACAGCCAAGGG

CCAAATGCCTCGTCATCTAATTAGTGACGCGCATGAATGGATTAACGAGATTCCCACTGTCCCTATCTACTATCTAGCGAAACCACAGCCAAGGG 8455

GLD8_421F
AACGGGCTT

D9 F
AAGACCCTGTTGAGYTTGACTC

AACGGGCTTGGCATAATCAGCGGGGAAAGAAGACCCTGTTGAGCTTGACTCTAGTTTGACATTGTGAAAGAACTTAGAGGGTGTAGCATAAGTGG 8550

GAGCTTCGGCGATCTTGAAATACCACTACCTTTATTGTTTTTTTACTTATTCAATAATGTGGAATTGGACTTCACGGTCCACTTTCTAGCGTTAA 8645

GGTTCTTTACGAACTGATCCAGTTTGAAGACATTGTCAGGTGGGGAGTTTGGCTGGGGCGGCACATCTGTTAAAAAATAACGCAGGTGTCCTAAG 8740

CCCTCAAACCGACCCCGCC
D10 R

TCAAACCGACCCCGCCGTGT
GLD8_677R

GGGAACTCATGGAGAACAGAAATCTCCAGTAGAACAAAAGGGTAAAAGTTCCCTTGATTTTGATTTTCAGTGTGAATACAAACCATGAAAGTGTG 8835

GCCTATCGATCCTTTAGATCCTGGAAATTTCAGGCTAGAGGTGTCAGAAAAGTTACCACAGGGATAACTGGCTTGTGGCAGCCAAGCGTTCATAG 8930

CGACGTTGCTTTTTGATCCTTCGATGTCGGCTCTTCCTATCATTAAGAAGCAGAATTCTTAAAGTGTTGGATTGTTCACCCACTAATAGGGAACG 9025

AGCTACAGCCGAGAAGGATAG
RB

TGAGCTGGGTTTAGACCGTCGTGAGACAGGTTAGTTTTCCCTACTGATGAAGTGTTGTTGCAATAGTAATTCAATTTAGTACGAGAGGAACCGTT 9120

GATTCGCATAATTGGTATTTGCGGTTAGTTGAAAAGCTAGTGCCGCGAAGCTACCATGCGTAGGATTATGGCTGAACGCCTCTAAGTCAGAATCC 9215

TGCGGAGATTCAGTCTTA
(R)_IGS

ATGCTAAAACGCGACGATTTTTACCCTCATTATATTTACGCGATTACAAATAGAGTTCGCTTCGGCGTTCTCCGGAATCATACAAGAGGACCCCG 9310

GTTCAGGCGTGGAAGTATGTCCTGTTCTCGTCCTTGAATGATAACTTCAAATATTAATGAGGATAAATCCTTTGCAGACGACTTAAATTTAGAAC 9405

CAGGTATTGTAAGCAGTAGAGTAGCCTTGTTGCTACGATCTGCTGAGATTAAGCCTTAGTTCTTTGATTTGT ••• 3'
9477
