# Supplementary material for: Full ribosomal operon sequencing of anaerobic gut fungi (phylum Neocallimastigomycota): insights on its markers and phylogenetic resolution
Source: IMA Fungus. 2026 Aug 26;17:e195921. doi: 10.3897/imafungus.17.195921 (PMC13539303; doi:10.3897/imafungus.17.195921)
Supplement: Supplementary file 1 [file imafungus-17-e195921-s001.zip › Supplementary_Data_1 to 14/Supplementary Data 7_Summary AGF morphology and rrn.pdf]

Supplementary Data 7: Summary of anaerobic gut fungal morphology and characteristics of the ribosomal operon

Genus	Species	Strains	Isolates	Families (Hanafy et al. 2023)	monocentric or polycentric	bulbous or rhizoidal	monoflagellated or polyflagellated	5S sense-oriented or inverted	max. consensus variants in any isolate			TATA box
									FUN	shm	rc	
Anaeromyces	*contortus*	BRP041, G3C, G3E	3	*Anaeromycetaceae*	polycentric	rhizoidal	monoflagellate	sense-oriented	1	3	3	both flanks
Anaeromyces	*mucronatus*	YoDo1, YoDo3, YoDo6	3	*Anaeromycetaceae*	polycentric	rhizoidal	monoflagellate	sense-oriented	2	2	7	both flanks
Capellomyces	*foraminis*	BGB12, BGS12, BGS2	3	*Anaeromycetaceae*	monocentric	rhizoidal	monoflagellate	sense-oriented	2	2	3	upstream
Liebetanzomyces	*polymorphus*	G6SC, ORC37	2	*Anaeromycetaceae*	monocentric	rhizoidal	monoflagellate	sense-oriented	4	2	1	upstream
Oontomyces	*anksri*	B2, L1(YC), L2, L5, X1, X19, X23, X29, etc.	35	*Anaeromycetaceae*	monocentric	rhizoidal	monoflagellate	inverted	-	3	6	downstream*
Caecomyces	*communis* var. *churrovis*	Brit4, NZ17-B7, PP313, ViSuPo, YoDo26	5	*Caecomycetaceae*	monocentric	bulbose	monoflagellate	sense-oriented	3	2	3	downstream
Caecomyces	*communis* var. *communis*	BRP07182, OF1, TSB4	3	*Caecomycetaceae*	monocentric	bulbose	monoflagellate	sense-oriented	3	2	3	downstream
Cyllamyces	*aberensis*	B32, TSB2	2	*Caecomycetaceae*	polycentric	bulbose	monoflagellate	sense-oriented	3	4	9	downstream
Aestipascuomyces	*dupliciliberans*	A252	1	*Neocallimastigaceae*	monocentric	rhizoidal	polyflagellate	sense-oriented	-	2	4	downstream*
Feramyces	*austinii*	DF1, F2b, F3a, R4A, NZ11-SL-2-SI-20	5	*Neocallimastigaceae*	monocentric	rhizoidal	polyflagellate	sense-oriented	5	5	5	downstream
Ghazallomyces	*constrictus*	ADS11, ADS12, AXS32	3	*Neocallimastigaceae*	monocentric	rhizoidal	polyflagellate	sense-oriented	1	3	5	both flanks
Neocallimastix	*cameroonii*	CaDo3a, YoDo11, YoDo12, YoDo13, etc.	5	*Neocallimastigaceae*	monocentric	rhizoidal	polyflagellate	sense-oriented	4	2	5	both flanks
Neocallimastix	*frontalis*	DWFs31, EKC11, GFMa3.1, TSS3, YoRe34,	36	*Neocallimastigaceae*	monocentric	rhizoidal	polyflagellate	sense-oriented	8	6	9	both flanks
Orpinomyces	*joyonii*	D3B, D4C, L1(ME), W212	4	*Neocallimastigaceae*	polycentric	rhizoidal	polyflagellate	inverted and sense-oriented	5	2	2	downstream or both flanks
Orpinomyces	new species-clade YL-X	NZ2-CF-4-CB-3	1	*Neocallimastigaceae*	polycentric	rhizoidal	polyflagellate	inverted	1	2	1	downstream*
Pecoramyces	*ruminantium*	OS2, YC1, A8, C1A, SA222, YoRe27, etc.	9	*Neocallimastigaceae*	monocentric	rhizoidal	mono- (bi-, tetra-) flagellate	sense-oriented	6	4	4	downstream
Agriosomyces	*longus*	MS2, MS4	2	not resolved	monocentric	rhizoidal	monoflagellate	sense-oriented	-	5	2	downstream*
Joblinomyces	*apicalis*	GFH682	1	not resolved	monocentric	rhizoidal	monoflagellate	sense-oriented	1	2	2	both flanks
Khoyollomyces	*ramosus*	H1WF70NRF6, X2152, ZC41, ZS32, etc.	9	not resolved	monocentric	rhizoidal	monoflagellate	sense-oriented	1	3	3	both flanks
Neocallimastigales	new genus-clade YL2 (Mara)	Mara4, Mara5	2	not resolved	uncultured	uncultured	uncultured	inverted	1	1	1	downstream
Paucimyces	*polynucleatus*	BB3	1	not resolved	polycentric	rhizoidal	monoflagellate	sense-oriented	-	5	2	downstream
Aklioshbomyces	*papillarum*	WT1, WT3, WT4	3	not resolved	monocentric	rhizoidal	mono- (tri-) flagellate	sense-oriented	4	6	3	downstream
Buwchfawromyces	*eastonii*	GE09	1	not resolved	monocentric	rhizoidal	monoflagellate	inverted	2	2	4	both flanks
NC3	new genus-clade NC3	CF6e	1	not resolved	uncultured	uncultured	uncultured	inverted	-	3	1	downstream*
Piromyces	*communis*	Jen.1, L10, P, UH3.1	4	*Piromycetaceae*	monocentric	rhizoidal	mono- (bi-, tetra-) flagellate	inverted	6	2	3	downstream
Piromyces	*cryptodigmaticus*	NZ30-BF2, NZ5-CF-3-CB-11, NZ9-CF-3-CB-	3	*Piromycetaceae*	monocentric	rhizoidal	mono- (bi-, tetra-) flagellate	inverted	8	4	2	downstream*
Piromyces	*finnis*	DonB21, H1	2	*Piromycetaceae*	monocentric	rhizoidal	mono- (bi-, tetra-) flagellate	inverted	1	1	2	downstream
Piromyces	*rhizinflatus*	CaDo1c, KiDo3e, NZ28-BF6	3	*Piromycetaceae*	monocentric	rhizoidal	mono- (bi-, tetra-) flagellate	inverted	3	2	2	downstream
Piromyces	sp. 6**	NZ10-CF-3-CB-19	1	*Piromycetaceae*	monocentric	rhizoidal	mono- (bi-, tetra-) flagellate	inverted	3	2	1	downstream*
Piromyces	sp. 7**	A1	1	*Piromycetaceae*	monocentric	rhizoidal	mono- (bi-, tetra-) flagellate	inverted	1	-	1	downstream
Cand. *Piromyces*	*potentiae*	B5	1	*Piromycetaceae*	monocentric	rhizoidal	mono- (bi-, tetra-) flagellate	inverted	1	2	3	downstream*
Tahromyces	*munnarensis*	BRP051	1	not resolved	monocentric	rhizoidal	monoflagellate	inverted	-	-	2	downstream*
**Total**			156									

Sources for morphological traits of each genus: Callaghan et al. (2015), Chang and Park (2020), Stabel et al. (2020), Hanafy et al. (2022), and Hanafy et al. (2023).

*TATA mainly found downstream, other locations in upstream were not complete.
