# Supplementary material for: Full ribosomal operon sequencing of anaerobic gut fungi (phylum Neocallimastigomycota): insights on its markers and phylogenetic resolution
Source: IMA Fungus. 2026 Aug 26;17:e195921. doi: 10.3897/imafungus.17.195921 (PMC13539303; doi:10.3897/imafungus.17.195921)

Supplementary Data 9: Alignment sketch

Example of the whole ribosomal operon alignment where the three amplicons (FUN: whole ribosomal operon or tande repeat (TR), rc: IGS, and shm: rRNA) are aligned together. A color is designed for each nucleotide (A: green; T: red; G: purple; C: blue). The boxes represent regions with high identity in the alignment. Note: this alignment is for demonstrative purposes, no trees were generated from this alignment.

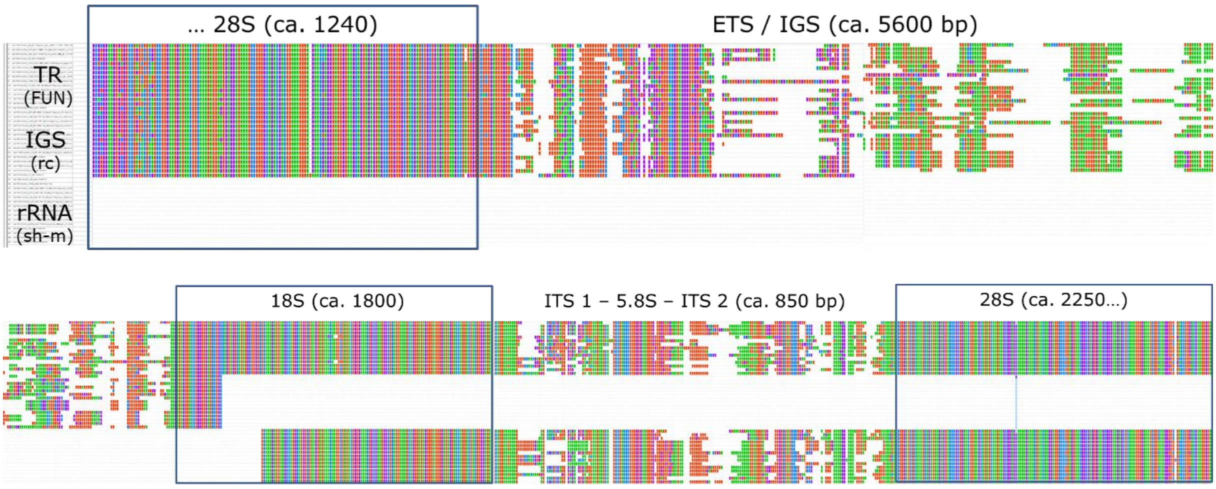

Supplement: Supplementary file 1 [file imafungus-17-e195921-s001.zip › Supplementary_Data_1 to 14/Supplementary Data 9_Alignment sketch.pdf]
